# Conjugation Lock-In Reinforced Sulfur-Heteropolycyclic Covalent Organic Frameworks with Asymmetric Electron Distribution for Photocatalytic Aerobic Oxidation Reactions

**DOI:** 10.1021/jacs.5c21545

**Published:** 2026-03-09

**Authors:** Jiani Yang, Shihuan Gao, Zhenyang Zhao, Xiaohui Xu, Siyuan Liu, Min Xu, Weichao Xue, Fan Dong, Shuang Li, Arne Thomas, Chong Cheng

**Affiliations:** † College of Polymer Science and Engineering, State Key Laboratory of Advanced Polymer Materials, 12530 Sichuan University, Chengdu 610065, China; ‡ Department of Ultrasound, Frontiers Science Center for Disease-Related Molecular Network, West China Hospital, Sichuan University, Chengdu 610041, China; § Research Center for Carbon-Neutral Environmental & Energy Technology, Institute of Fundamental and Frontier Sciences, 12599 University of Electronic Science and Technology of China, Chengdu 611731, China; ∥ College of Chemistry, Sichuan University, Chengdu 610065, China; ⊥ Functional Materials, Department of Chemistry, Technische Universität Berlin, Hardenbergstraße 40, Berlin 10623, Germany; # Department of Endodontics, State Key Laboratory of Oral Diseases, National Center for Stomatology, West China Hospital of Stomatology, Sichuan University, Chengdu 610041, China

## Abstract

The development of robust heterogeneous photocatalysts capable of operating under harsh chemical conditions remains a critical yet challenging goal in materials science. Here, we present a postcyclization strategy based on conjugation lock-in reinforcement to construct sulfur-heteropolycyclic covalent organic frameworks (COFs) with asymmetric electron distribution for superior photocatalytic reactions, which is achieved through a consecutive thionation–cyclization–oxidation transformation of *N*-acylhydrazone precursors using Lawesson’s reagent. Systematic characterization reveals that sulfur incorporation endows the framework with enhanced chemical stability, localized asymmetric electron density, rapid charge carrier migration, efficient exciton dissociation, extended π-conjugation, and a well-defined donor–acceptor architecture, collectively leading to significantly improved photocatalytic activity and durability compared to the original hydrazone-linked COF. Consequently, the modified COF exhibits a H_2_O_2_ evolution rate of 5270 μmol g^–1^ h^–1^ in pure water, representing a 3-fold enhancement over the precursor COF (1878 μmol g^–1^ h^–1^) and surpassing most reported organic and inorganic competitors. Moreover, it achieves complete conversion of benzylamine within 1 h under mild blue light-emitting diode irradiation, demonstrating high efficiency in aerobic oxidation catalysis. These findings establish the lock-in reinforcement strategy coupled with electronic structure modulation as a versatile route to designing durable and highly active photocatalysts for demanding synthetic and energy-conversion applications.

## Graphical abstract

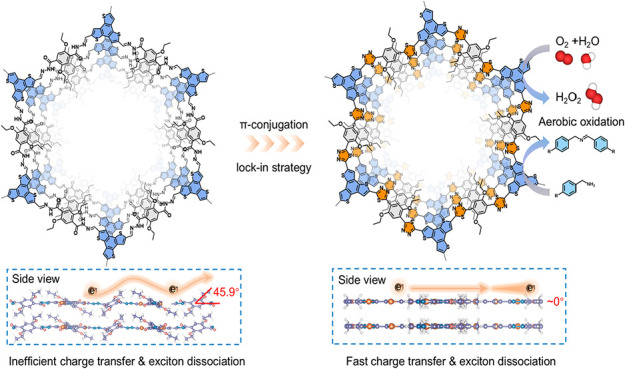

## Introduction

Photocatalytic aerobic oxidation aligns with the principles of green chemistry and has been extensively employed in the oxidative transformation of sulfides, alcohols, secondary amines, benzyl halides, and related substrates.^1–4^ In such processes, reactive oxygen species (ROS) generated catalytically upon light irradiation often mediate efficient and environmentally benign reaction pathways, rendering photocatalysis a promising strategy for aerobic oxidation.^5–7^ Considerable research effort has thus been devoted to developing diverse photocatalytic systems, including transition-metal-based composites,^8–12^ polyoxometalates,^13,14^ and coordination complexes.^15–19^ Among emerging candidates, polyaromatic covalent organic frameworks (COFs), a class of crystalline conjugated polymers constructed from lightweight organic building blocks linked by covalent bonds,^20–26^ have garnered increasing interest owing to their tunable electronic band structures,^27–30^ high density of accessible catalytic sites, and exceptional photochemical stability. These attributes position COFs as attractive platforms for photocatalytic aerobic transformations.^31–33^

The construction of high-performance and robust COFs for photocatalytic applications critically depends on the design of highly conjugated architectures. Such architectures can be achieved either through the direct synthesis of COFs featuring intrinsically conjugated linkages (e.g., sp^2^ C=C or C≡C linkages) or via postsynthetic cyclization strategies.^34–39^ However, the direct synthesis of these highly conjugated COFs remains challenging. For example, the formation of sp^2^-carbon-linked COFs is constrained by the limited reversibility of C=C bond formation and the stringent conditions required for crystallization, which together restrict the structural diversity of accessible building blocks.^40–42^ In this context, cyclization strategies have emerged as a promising alternative for developing high-efficiency photocatalytic COFs. To date, considerable efforts have focused on the cyclization of imine-linked COFs.^43–47^ Notable examples include the Povarov reaction-based postsynthetic modification reported by Tan and co-workers to generate quinoline-linked COFs,^48^ as well as the synthesis of a 12 + 3-connected three-dimensional imidazole-linked COF with **aea** topology by Negishi and colleagues.^49^

Nevertheless, a growing body of recent work has demonstrated that imine-linked COFs often exhibit inferior photocatalytic performance compared to their hydrazone-linked counterparts.^38,50,51^ The hydrazone linkage is a unique bonding mode classified as a type of Schiff-base derivative, which inherits the intrinsic functionalities of conventional Schiff-base linkages while endowing COFs with novel physicochemical properties through the rational selection of structural building units.^52^ Despite these advantages, the intrinsic reversibility and strong polarization of hydrazone bonds can compromise framework stability and disrupt π-electron delocalization along the conjugated backbone.^34,53–55^ Thus, to realize COF architectures that simultaneously possess excellent optoelectronic properties and robust chemical stability, it is essential to systematically investigate the cyclization behavior of hydrazone-linked COFs and, in particular, to elucidate their underlying photocatalytic mechanisms. However, such investigations remain limited, and studies exploring their application in photosynthesis, such as aerobic oxidation reactions, are virtually absent. This gap underscores the need for further exploration of cyclized hydrazone-linked COFs as a platform for advanced photocatalytic transformations.

Building on prior conceptual advances,^56,57^ we utilized a conjugation lock-in reinforcement strategy coupled with asymmetric electron distribution to synthesize a thiadiazole-based sulfur-heteropolycyclic COF (TDA-BTT-COF) for superior photocatalytic reactions. This new COF photocatalyst was constructed through sequential thionation, cyclization, and oxidation of N-acylhydrazone precursors using Lawesson’s reagent as the thionating agent (Figure 1a,b). Our research is driven by four primary purposes: first, sulfur incorporation into thiadiazole rings induces localized asymmetric electron density (Figure 1c,d); second, the thiadiazole architecture enhances intramolecular polarity, reinforces donor–acceptor character, and promotes rapid exciton dissociation (Figure 1e,f); third, the locked-in coplanar conformation facilitates efficient charge transport along an extended π-conjugated plane (Figure 1g); and fourth, heterocyclic and heteropolycyclic stabilization significantly improves chemical robustness, thereby elevating intrinsic photocatalytic performance. Compared to the hydrazone-linked precursor (HZ-BTT-COF), TDA-BTT-COF exhibits superior stability under harsh conditions, extended in-plane π-conjugation, and enhanced optoelectronic properties. These structural advantages translate to a photosynthetic H_2_O_2_ production rate of 5270 μmol g^–1^ h^–1^, which is three times greater than that of the hydrazone-linked COF (1878 μmol g^–1^ h^–1^) and surpasses many leading organic and inorganic competitors. Moreover, TDA-BTT-COF achieves complete benzylamine conversion within 1 h under low-power blue irradiation, demonstrating exceptional activity in photocatalytic aerobic oxidation. This work establishes lock-in reinforcement combined with electronic structure modulation as a generalizable approach to designing high-performance heterogeneous photocatalysts for demanding synthetic applications.

**Figure 1:**
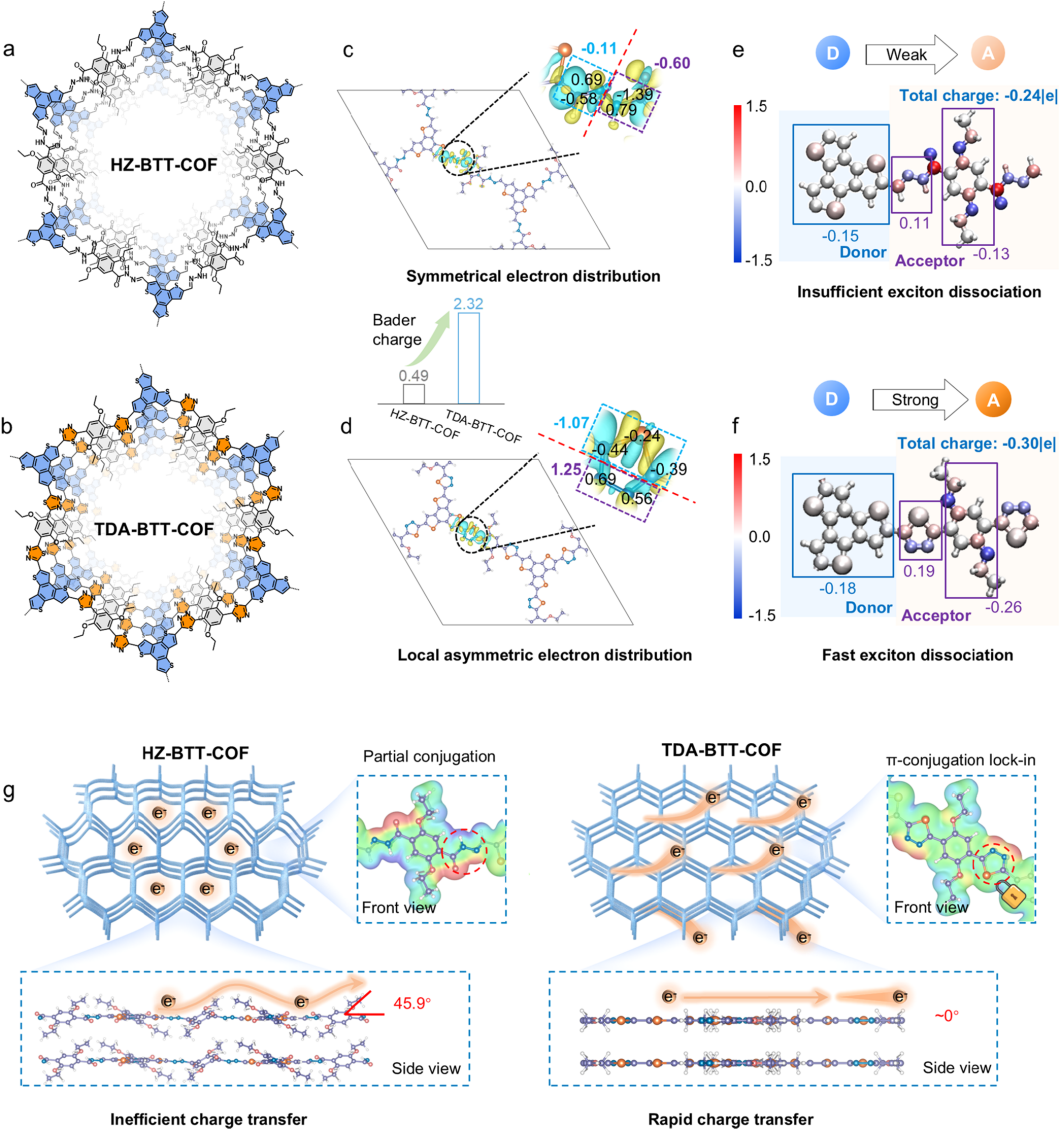
Structure design and advantage of sulfur-heteropolycyclic COFs via postcyclization reaction. The chemical structures of (a) HZ-BTT-COF and (b) TDA-BTT-COF. The charge density differences and Bader analysis of (c) HZ-BTT-COF and (d) TDA-BTT-COF (yellow and cyan represent charge accumulation and depletion, respectively, with a cutoff value of 0.003 e·bohr^–3^ for the density-difference isosurface). Atom colors: C, purple; N, blue; O, pink; S, orange; H, white. The Bader charge coloring distribution of (e) HZ-BTT-COF and (f) TDA-BTT-COF. (g) The illustration of the front and side view structures and charge transfer pathway comparisons of HZ-BTT-COF and TDA-BTT-COF. Atom colors: C, purple; N, blue; O, pink; S, orange; H, white.

## Results and Discussion

### Synthesis and Characterization of COFs

The synthesis began with the preparation of HZ-BTT-COF through solvothermal condensation of 2,5-diethoxyterephthalohydrazide and benzo­[1,2-b:3,4-b′:5,6-b″]­trithiophene-2,5,8-tricarbaldehyde (BTT) under optimized conditions (180 °C, 72 h), yielding a crystalline yellow powder. This intermediate framework was subsequently converted into the target sulfur-heteropolycyclic TDA-BTT-COF via a postsynthetic cyclization strategy using Lawesson’s reagent in toluene. The transformation proceeds through sequential thionation, cyclization, and oxidation steps, effectively converting hydrazone linkages into fully conjugated thiadiazole units while preserving crystallinity. A model reaction between benzaldehyde and benzoyl hydrazine was initially conducted, and the successful formation of a model compound (synthetic details in the Supporting Information) confirmed the viability of this strategy to form robust COF linkages (Figures S1–S2). This approach provides a versatile route to heteroaromatic COFs with tailored electronic properties and enhanced structural stability.

The crystalline structures of HZ-BTT-COF and TDA-BTT-COF were characterized by powder X-ray diffraction (PXRD), with both materials exhibiting sharp diffraction peaks indicative of high crystallinity. Pawley refinement of the PXRD profiles confirmed that each framework adopts a hexagonal P6/M space group with AA stacking, achieving excellent agreement between experimental and simulated patterns (Figure 2a,d). For TDA-BTT-COF, the refined unit cell parameters were *a* = *b* = 37.04 Å and *c* = 3.41 Å, with agreement factors of *R*_wp_ = 3.45% and *R*_p_ = 2.46%, indicating a high-quality structural model. A larger interlayer stacking distance “c” from 3.39 Å in HZ-BTT-COF to 3.41 Å in TDA-BTT-COF was observed, attributable to the introduction of sulfur into the layers and the strengthening of the π-conjugation effect between layers. (Figures S3–S8 and Tables S1, S2).

**Figure 2:**
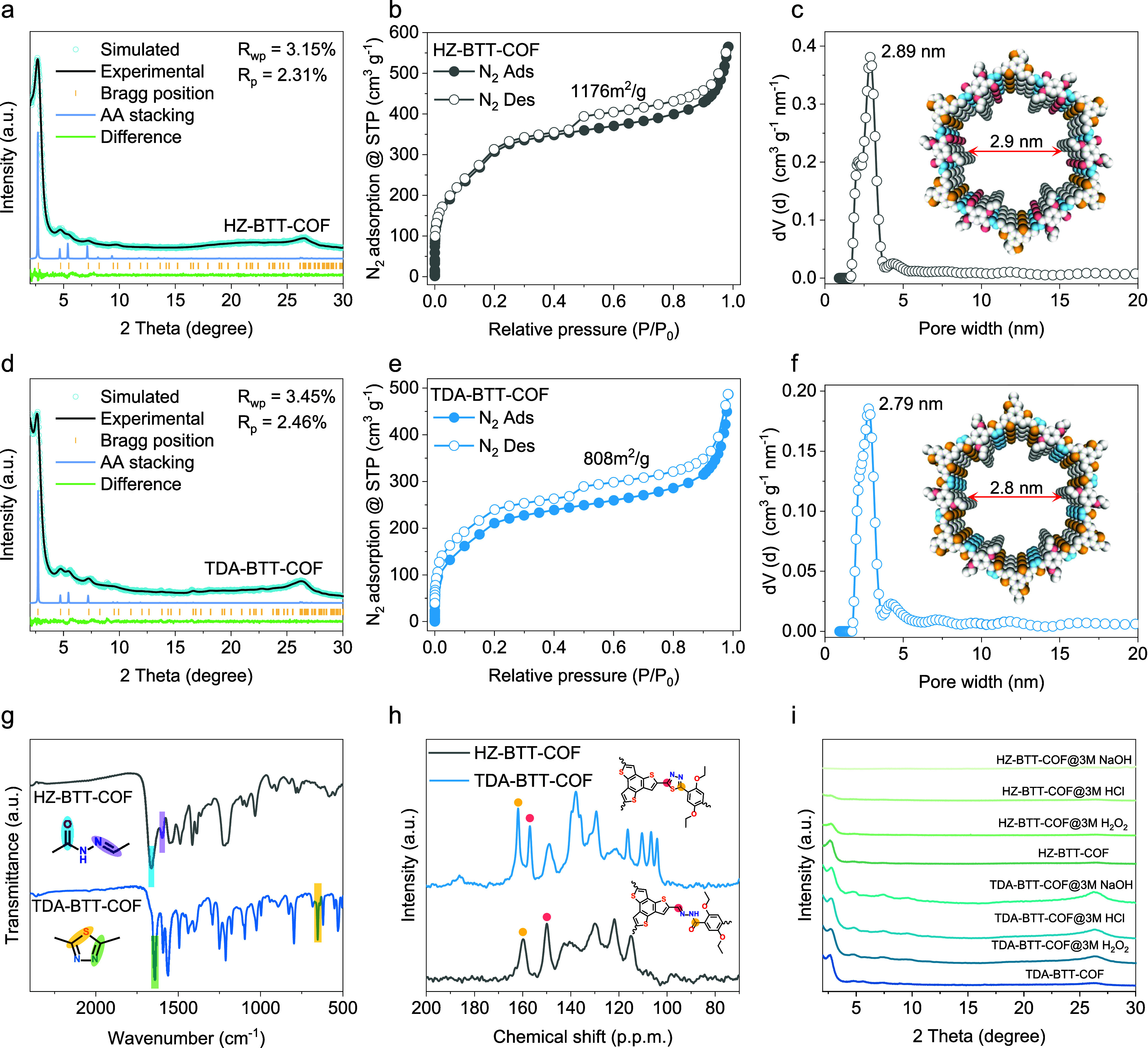
Chemical structure properties of photocatalytic COFs. (a) Pawley refinement of the experimental PXRD patterns of HZ-BTT-COF. Experimental (black line), Pawley fitted (blue line), simulated (blue dot), and difference (green line) are displayed. Bragg positions are marked by orange bars. (b) N_2_ adsorption–desorption isotherm of HZ-BTT-COF. (c) The pore size distributions of HZ-BTT-COF. (d) Pawley refinement of the experimental PXRD patterns of TDA-BTT-COF. (e) N_2_ adsorption–desorption isotherm of TDA-BTT-COF. (f) The pore size distributions of TDA-BTT-COF. (g) FTIR spectra of HZ-BTT-COF and TDA-BTT-COF. (h) ^13^C ssNMR demonstrates the conversion of the imine linkage to the corresponding thiadiazole. (i) Chemical stability test of HZ-BTT-COF and TDA-BTT-COF after 12 h of treatment with 3 M HCl, 3 M NaOH, and 3 M H_2_O_2_.

The porous properties of HZ-BTT-COF and TDA-BTT-COF were assessed using nitrogen sorption measurements at 77 K (Figure 2b,e). The Brunauer–Emmett–Teller (BET) surface area was determined to be 1176 m^2^ g ^–1^ for HZ-BTT-COF and 808 m^2^ g ^–1^ for TDA-BTT-COF. Nonlocal density functional theory (NLDFT) pore size distribution profiles indicated uniform mesopores centered at approximately 2.89 and 2.79 nm for HZ-BTT-COF and TDA-BTT-COF, respectively, in good agreement with the theoretical pore sizes calculated from the generated model structures (Figure 2c,f). Scanning and transmission electron microscopy (SEM/TEM) revealed that both materials maintain a similar rod-like morphology (Figures S9 and S10). Elemental mapping via energy-dispersive X-ray spectroscopy (EDS) further confirmed the homogeneous distribution of carbon, nitrogen, oxygen, and sulfur throughout the COF architecture, verifying successful sulfur incorporation into the framework (Figures S11–S12). Together, these structural analyses affirm the integrity and compositional uniformity of TDA-BTT-COF, providing a foundation for its subsequent photocatalytic evaluation.

The successful formation of hydrazone-linked HZ-BTT-COF was initially verified by Fourier-transform infrared (FTIR) spectroscopy, with characteristic stretching vibrations observed at 1670 cm^–1^ (C=O) and 1610 cm^–1^ (N=CH), confirming the presence of the hydrazone linkage (Figure 2g). Conversion to TDA-BTT-COF was evidenced by the disappearance of these hydrazone signals and the emergence of new absorptions at 1641 cm^–1^ (N=C) and 653 cm^–1^ (C–S), consistent with thiadiazole ring formation. Solid-state ^13^C nuclear magnetic resonance (ssNMR) spectroscopy further corroborated the structural transformation (Figure 2h). In the spectrum of HZ-BTT-COF, resonances at 159.7 and 149.8 ppm were assigned to the carbonyl carbon and the hydrazone-linked methine carbon, respectively. Following postcyclization, TDA-BTT-COF exhibited two new signals at 161.9 and 156.8 ppm, attributable to carbon atoms within the thiadiazole ring, confirming the conversion of hydrazone units into the heteroaromatic system.

X-ray photoelectron spectroscopy (XPS) analysis provided further evidence for the formation of thiadiazole linkages in TDA-BTT-COF (Figures S13–S14). A key advantage of these thiadiazole-based frameworks is their superior chemical stability relative to hydrazone-linked analogs. To evaluate this property, we exposed activated TDA-BTT-COF samples to aggressive aqueous media, such as 3 M HCl (pH < 0), 3 M NaOH (pH > 14), and 3 M H_2_O_2_, for 12 h. Post-treatment PXRD patterns revealed that TDA-BTT-COF retained its crystallinity under all conditions, with diffraction peaks remaining as sharp as those of the pristine material. By contrast, HZ-BTT-COF maintained slight crystallinity only in H_2_O_2_, undergoing structural degradation in acidic and basic environments (Figure 2i). To further demonstrate the enhanced stability of the COF modified via the conjugation lock-in strategy, we synthesized imine-linked (im-BTT-COF) and C=C-linked (sp^2^-BTT-COF) COFs with analogous structures. Likewise, after soaking these COFs under various harsh conditions, their crystal structures were characterized by PXRD measurements. The results revealed that the im-BTT-COF exhibited complete loss of crystallinity in acidic and alkaline solutions, with only weak crystalline peaks observed in the H_2_O_2_ solution. In contrast, the sp^2^-BTT-COF, which inherently suffers from high synthetic difficulty and low initial crystallinity, showed an almost complete loss of crystallinity following the soaking treatment (Figure S15). Moreover, the thermogravimetric analysis experiment shows that TDA-BTT-COF exhibits better thermal stability up to ∼460 °C than HZ-BTT-COF (Figure S16). These results confirm not only the successful synthesis of heteroaromatic TDA-BTT-COF but also its exceptional stability under chemically challenging conditions, a crucial characteristic for photocatalytic applications in demanding settings.

### Photocatalytic H_2_O_2_ Production

Following successful structural confirmation of HZ-BTT-COF and TDA-BTT-COF, we evaluated their optical properties and electronic band structures. UV–visible diffuse reflectance spectroscopy (DRS) showed broad visible-light absorption for both materials, with TDA-BTT-COF exhibiting extended absorption between 550–600 nm due to enhanced π-conjugation through thiadiazole linkages (Figure 3a). This optical behavior corresponded to the yellow and orange appearance of HZ-BTT-COF and TDA-BTT-COF, respectively. Tauc plot analysis derived from Kubelka–Munk-transformed DRS data yielded optical band gaps (*E*_g_) of 2.51 eV for HZ-BTT-COF and 2.31 eV for TDA-BTT-COF. Mott–Schottky measurements gave flat-band potentials of −1.03 V and −0.69 V versus NHE (normal hydrogen electrode) for HZ-BTT-COF and TDA-BTT-COF, respectively (Figure S17). Combining these results, we constructed energy band diagrams using the relationship *E*_CB_ = *E*_VB_ – *E*_g_ (Figure 3b), placing the conduction band (CB) edges at −0.83 V and −0.49 V, and valence band (VB) edges at 1.68 and 1.82 V versus NHE for HZ-BTT-COF and TDA-BTT-COF. The CB positions of both COFs are sufficiently negative to drive the 2e^–^ oxygen reduction reaction (ORR) for H_2_O_2_ production (−0.33 V for indirect and 0.68 V for direct ORR at pH 0, vs *NHE*), and this trend can also be seen through the CV test (Figure S18). Moreover, the VB of TDA-BTT-COF exceeds the potential required for the two-electron water oxidation reaction (WOR) (1.76 V vs *NHE*), enabling H_2_O_2_ generation via both ORR and WOR pathways, whereas HZ-BTT-COF lacks this oxidative route.

**Figure 3:**
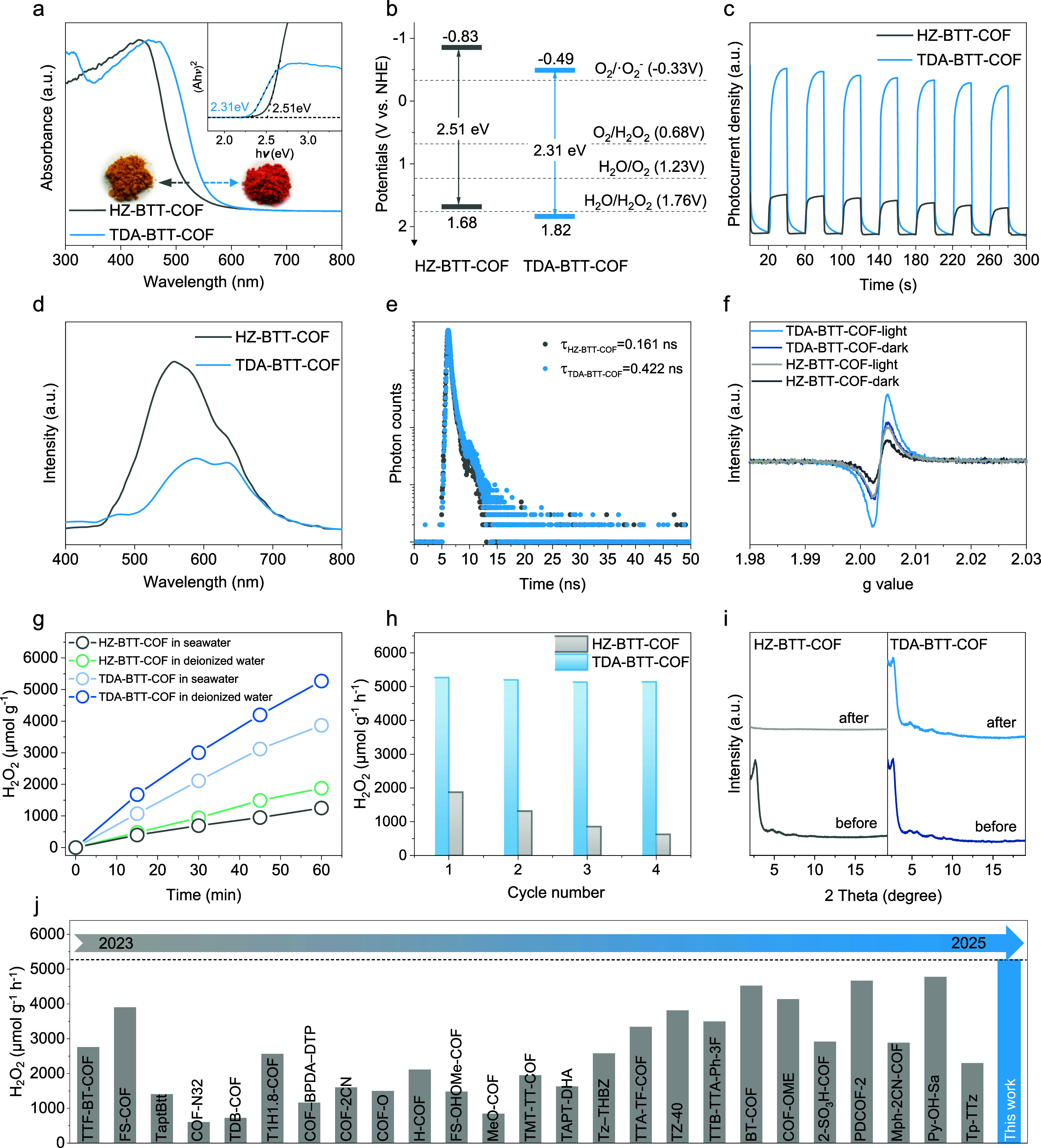
Photophysical characterization and photocatalytic H_2_O_2_ generation performance. (a) Solid-state UV–vis diffuse reflectance spectra and Tauc plot for band gaps calculation. (b) Band-structure diagrams of HZ-BTT-COF and TDA-BTT-COF. (c) Photocurrent responses of HZ-BTT-COF and TDA-BTT-COF. (d) Steady-state photoluminescence (PL) measurements of HZ-BTT-COF and TDA-BTT-COF. (e) Time-resolved fluorescence spectroscopy of HZ-BTT-COF and TDA-BTT-COF excited at 370 nm, and the emission wavelength is 580 nm. (f) EPR spectra of HZ-BTT-COF and TDA-BTT-COF in the dark and under visible-light illumination. (g) Photocatalytic activity of HZ-BTT-COF and TDA-BTT-COF for H_2_O_2_ production (5 mg of COFs in 25 mL of deionized water and seawater at 25 °C and 300 W Xe lamp, λ > 420 nm). (h) Photocatalytic activity of HZ-BTT-COF and TDA-BTT-COF for H_2_O_2_ production (5 mg of COFs in 25 mL of deionized water at 25 °C and 300 W Xe lamp, λ > 420 nm). (i) PXRD patterns of HZ-BTT-COF and TDA-BTT-COF before and after four cycles (COFs were regenerated by washing with acetone and MeOH). (j) A comparison of the H_2_O_2_ production performance of TDA-BTT-COF with previously reported photocatalysts.

The separation and transport dynamics of photogenerated charge carriers in HZ-BTT-COF and TDA-BTT-COF were investigated using transient photocurrent measurements. Both materials exhibited prompt and reproducible photocurrent generation under chopped illumination, with TDA-BTT-COF yielding a consistently higher photocurrent density than HZ-BTT-COF, reflecting more efficient charge separation and migration (Figure 3c). Electrochemical impedance spectroscopy (EIS) further supported this observation, as TDA-BTT-COF displayed a smaller semicircle in the Nyquist plot, indicating lower charge-transfer resistance (Figure S19). Steady-state photoluminescence (PL) spectra showed markedly reduced emission intensity for TDA-BTT-COF relative to HZ-BTT-COF, consistent with suppressed radiative recombination (Figure 3d). Time-resolved fluorescence decay profiles, obtained under 370 nm excitation, yielded average carrier lifetimes of 0.161 ns for HZ-BTT-COF and 0.422 ns for TDA-BTT-COF, with the extended lifetime in the latter confirming slower charge recombination (Figure 3e).

Femtosecond-transient absorption (fs-TA) spectroscopy was then conducted on both COFs to investigate the exciton dynamics in the femtosecond-to-picosecond time domain. As depicted in Figure S20, the negative feature at ca. 530 nm can be assigned to the ground-state bleaching (GSB), reflecting the inverse ground-state diffuse reflectance spectrum. The broad positive absorption band (>600 nm) belongs to the excited state absorption (ESA) (S1 → Sn). However, only positive ESA signals are present in the transient absorption spectra of HZ-BTT-COF, and no distinct GSB response is observed throughout the detection range. Meanwhile, the kinetic curve at 630 nm verifies that HZ-BTT-COF undergoes ultrafast recombination immediately upon photoexcitation without any intermediate dynamic process. These results demonstrate that TDA-BTT-COF exhibits efficient charge separation, a high free-carrier yield, and excellent carrier migration. In contrast, HZ-BTT-COF exhibits a photophysical behavior dominated by exciton recombination, with extremely low charge separation efficiency and severely restricted generation of free charge carriers. Upon photoexcitation, the establishment of the ESA signal for TDA-BTT-COF required 1 ps, while HZ-BTT-COF required only 0.7 ps, further confirming that the locked-in molecular architecture inhibits the rapid recombination of photoexcited electron–hole pairs. Electron paramagnetic resonance (EPR) spectroscopy revealed a stronger signal at *g* = 2.0083 for TDA-BTT-COF under both dark and illuminated conditions,^58^ indicative of enhanced delocalization of unpaired electrons within the π-conjugated framework (Figure 3f). These findings indicate that the thiadiazole-based heteroaromatic COF structure promotes extended π-delocalization and strengthens donor–acceptor interactions, leading to improved charge separation, prolonged carrier lifetime, and superior photocatalytic activity.

Photocatalytic H_2_O_2_ production was evaluated under ambient conditions (25 °C) in deionized water and natural seawater, with continuous O_2_ purging and no sacrificial agents. Reactions were irradiated using a xenon lamp (λ > 420 nm), and H_2_O_2_ yields were determined by iodometric titration (see Supporting Methods and Figure S21). Both COFs exhibited light-dependent H_2_O_2_ generation, with TDA-BTT-COF achieving a production rate of 5270 μmol g^–1^ h^–1^ in oxygenated deionized water, thus significantly outperforming HZ-BTT-COF (1878 μmol g^–1^ h^–1^) (Figure 3g). A comparative analysis of the H_2_O_2_ production performance of TDA-BTT-COF with various state-of-the-art photocatalysts reported in recent years is presented in Figure 3j. TDA-BTT-COF presents superior photocatalytic activities in terms of production rates, surpassing the majority of H_2_O_2_-generating photocatalysts documented to date (Supporting Table 3). More importantly, with analogous monomer structures but different bonding modes, the TDA-BTT-COF exhibits superior photocatalytic performance to the im-BTT-COF and sp^2^-BTT-COF (Figure S22). This result highlights the critical role of thiadiazole linkages in enhancing photocatalytic activity. The practicability of direct photosynthesis of H_2_O_2_ using the TDA-BTT-COF photocatalyst was further explored. TDA-BTT-COF can achieve efficient photosynthesis of H_2_O_2_ directly, even from more readily available water sources such as seawater, rainwater, tap water, saline water, and river water, obviating the need for sacrificial reagents—an important feature for scalable, cost-effective photosynthetic applications (Figure S23).

The photocatalytic versatility of TDA-BTT-COF was further demonstrated under varied reaction conditions. In the presence of different sacrificial agents, benzyl alcohol (BnOH) afforded the highest H_2_O_2_ production rate (12,500 μmol g^–1^ h^–1^), attributable to a biphasic water–BnOH system that suppresses H_2_O_2_ decomposition by confining the COF catalyst within the organic phase while allowing rapid diffusion of the product into the aqueous phase (Figure S24). The apparent quantum yield (AQY) of TDA-BTT-COF reached 9.5% at 450 nm in oxygen-saturated deionized water (Figure S25). Moreover, the material achieved a solar chemical conversion efficiency of 0.27%, exceeding the typical photosynthetic efficiency of natural plants (∼0.10%).^59^ These results demonstrate the exceptional photocatalytic performance of TDA-BTT-COF and its potential for applications in artificial photosynthesis and the environment, where efficient H_2_O_2_ generation is crucial.

The steady-state concentration of H_2_O_2_ reflects a dynamic balance between its formation and decomposition over the catalyst surface. Both HZ-BTT-COF and TDA-BTT-COF maintained over 95% of generated H_2_O_2_ under continuous illumination for 1 h (Figure S26). A key advantage of heterogeneous catalysts is their recyclability, which we evaluated over four consecutive photocatalytic cycles (Figure 3h). HZ-BTT-COF showed a sharp decline in H_2_O_2_ production after the first cycle, accompanied by partial loss of crystallinity, as indicated by attenuated PXRD peaks. In contrast, TDA-BTT-COF showed no significant activity loss over four cycles and retained its original crystalline structure (Figure 3i). FTIR spectroscopy further confirmed that the chemical composition of TDA-BTT-COF remained unchanged after repeated use (Figure S27). Meanwhile, after testing in seawater, the TDA-BTT-COF still maintained good chemical and structural stability (Figure S28). These results highlight the structural robustness and excellent recyclability of TDA-BTT-COF, reinforcing its potential as a durable photocatalyst for sustainable H_2_O_2_ production.

### Photocatalytic Mechanisms and Pathways

To elucidate the photocatalytic mechanism underlying H_2_O_2_ production in this study, we conducted a series of experiments, including active intermediates capture, EPR, rotating disk electrode (RDE), in situ diffuse reflectance infrared Fourier transform spectroscopy (DRIFTS), and isotope-labeling measurements. When the holes were trapped in the presence of CH_3_OH and O_2_ (Figure 4a), the H_2_O_2_ production for TDA-BTT-COF showed a downward trend, while this for HZ-BTT-COF showed an upward trend (Figure S29). This phenomenon indicates that holes generated from HZ-BTT-COF may not be directly involved in the photocatalytic production of H_2_O_2_. When O_2_ was replaced by N_2_ in the reaction system, the yield of H_2_O_2_ decreased significantly for the two COFs. Compared with the N_2_-only condition, the yield of H_2_O_2_ increased in TDA-BTT-COF when the electron-trapping agent (AgNO_3_) was added in the presence of N_2_. However, the H_2_O_2_ concentration was almost undetectable for HZ-BTT-COF under the same conditions. This result implies that a 4e^–^ WOR may have occurred in HZ-BTT-COF (2H_2_O + 4h^+^ → O_2_ + 4H^+^), while a 2e^–^ WOR may have occurred in TDA-BTT-COF (2H_2_O + 2h^+^ → H_2_O_2_ + 2H^+^).

**Figure 4:**
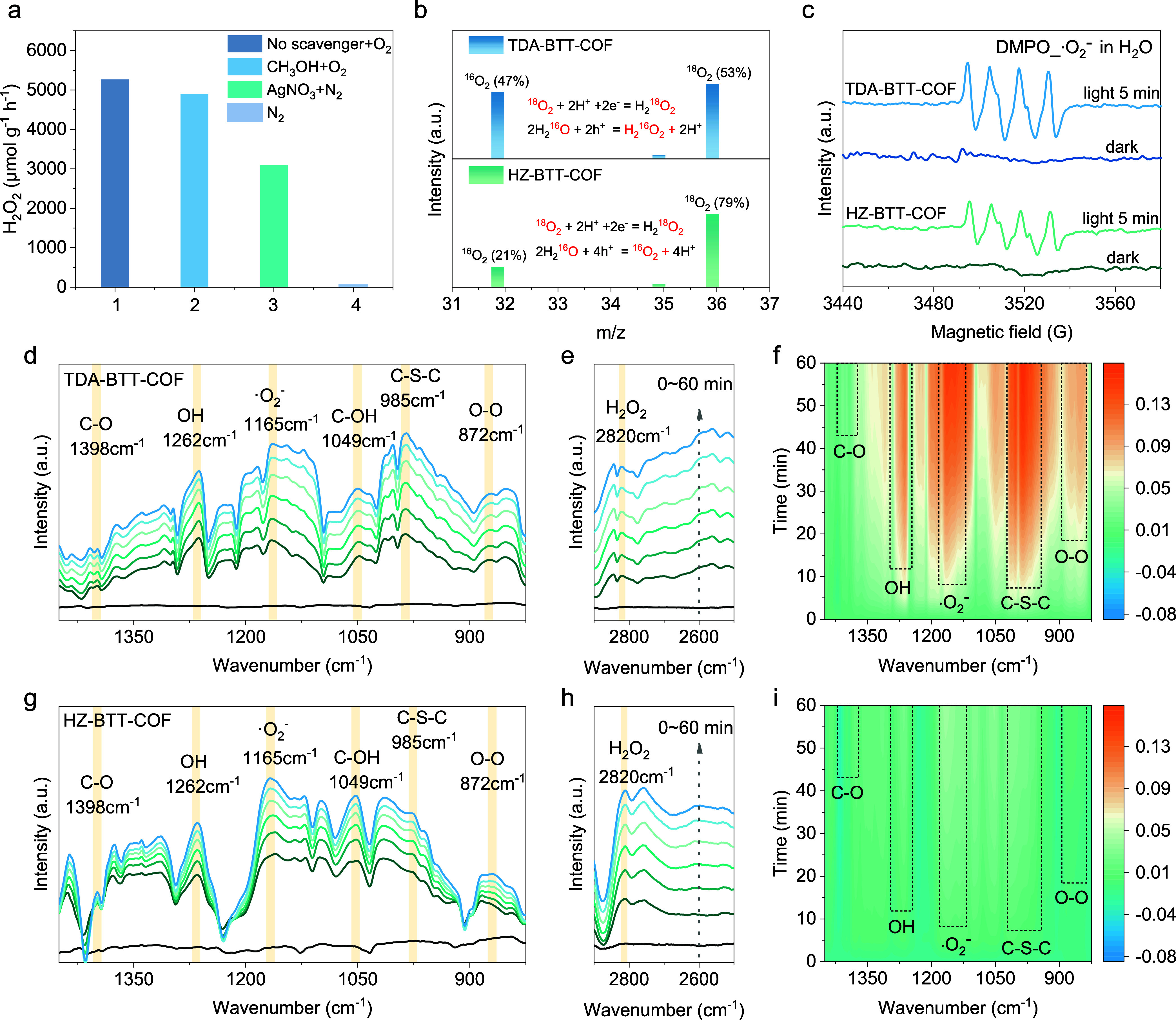
Reaction pathways and mechanisms of H_2_O_2_ photosynthesis. (a) Photocatalytic H_2_O_2_ production for TDA-BTT-COF in CH_3_OH (10% v/v, as the hole trapping agent), H_2_O_2_ produced in N_2_ and AgNO_3_ (0.01 M). Conditions: water (25 mL), catalyst (5 mg), 300 W Xe lamp, λ > 420 nm. (b) ^18^O_2_ isotope experiment to explore the source of H_2_O_2_. (c) EPR signals of the reaction solution under dark and visible light illumination in the presence of DMPO as the spin-trapping reagents. (d–f) In-situ DRIFT spectra of TDA-BTT-COF. (g–i) In-situ DRIFT spectra of HZ-BTT-COF.

In addition, ^18^O_2_ isotope experiments have been carried out to verify the ORR and WOR processes (Figure 4b). The COFs were irradiated in H_2_^16^O and ^18^O_2_ gas for 4 h. After removing unreacted gas with Ar gas, MnO_2_ was added to the reaction system to decompose H_2_O_2_ and release O_2_. The escaped gas was analyzed by gas chromatography–mass spectrometry (GC-MS). The decomposition products of photogenerated H_2_O_2_ through the MnO_2_ reaction are close to 1:1 for ^18^O_2_ and ^16^O_2_, which shows that the H_2_O_2_ photosynthesis process in TDA-BTT-COF experiences both the 2e^–^ ORR and 2e^–^ WOR pathways. However, a much higher ratio of ^18^O_2_ and ^16^O_2_ for HZ-BTT-COF was observed, indicating that H_2_^18^O_2_ was the dominant product that came from the reduction of ^18^O_2_ and confirming the 4e^–^ WOR process in HZ-BTT-COF.

EPR analysis and RDE tests were then employed to provide further insight into the ORR reaction pathways. EPR measurements were performed in methanol using 5,5-dimethyl-1-pyrroline N-oxide (DMPO) as a free-radical spin-trap agent. As depicted in Figure 4c, upon visible light irradiation, the EPR spectra of both TDA-BTT-COF and HZ-BTT-COF reaction systems exhibited characteristic signals indicative of ^•^O_2_^–^. In contrast, no signals were detected in the absence of light, confirming the formation of ^•^O_2_^–^ within the photocatalytic system. However, the presence of hydroxyl radicals (^•^OH) and singlet oxygen (^1^O_2_) was not detected in the EPR measurements, which rules out the possibility of the 1e^–^ WOR process and the participation of ^1^O_2_ during the H_2_O_2_ photosynthesis in TDA-BTT-COF (Figure S30). Besides, we measured linear sweep voltammetry (LSV) curves using RDE for HZ-BTT-COF and TDA-BTT-COF at various rotation speeds (100–900 rpm), as displayed in Figure S31. By plotting the curves with the Koutecky–Levich method, the average electron transfer numbers of HZ-BTT-COF and TDA-BTT-COF were calculated to be 1.87 and 1.23, respectively (Figure S32). These results suggest that HZ-BTT-COF reduced O_2_ mainly via the direct 2e^–^ pathway (O_2_ + 2e^–^ + 2H^+^ → H_2_O_2_), while the stepwise 1e^–^ pathway contributes more to the photosynthesis of H_2_O_2_ in the TDA-BTT-COF based photocatalytic systems (O_2_ + e^–^ → ^•^O_2_^–^, ^•^O_2_^–^ + 2H^+^ + e^–^ → H_2_O_2_).

To further support the formation of reactive intermediates as well as the photocatalytic pathway, we performed in situ DRIFTS tests on HZ-BTT-COF and TDA-BTT-COF (Figure 4d–i) under an oxygen and steam environment. Noticeably, the peak at 2820 cm^–1^ corresponding to O–H bending of H_2_O_2_ (Figure 4e,h) gradually increased with photoirradiation time, suggesting the formation of H_2_O_2_. Peaks originating from C–S–C stretching BTT unit (985 cm^–1^) also varied in intensity with photoirradiation time, suggesting their involvement as active sites in the reaction pathway. Peaks corresponding to C–O (1398 cm^–1^), ^•^O_2_^–^ (1165 cm^–1^), and O–O (872 cm^–1^) also increased in intensity (Figure 4d,g), signifying the adsorption of O_2_ and the occurrence of the two-step single-electron pathway in the photocatalytic system based on COFs. Finally, signals belonging to C–OH (1049 cm^–1^) and OH (1262 cm^–1^) are observed in the in situ DRIFT spectra, which suggests that H_2_O adsorbed onto the surfaces of COF can be dissociated into *OH. In addition, as shown in Figure 4f,i, the peak change in HZ-BTT-COF is less pronounced than that in TDA-BTT-COF under the same conditions, further explaining why TDA-BTT-COF exhibits better photocatalytic performance. The insights gained from these experiments provide a robust foundation for further optimizing the photocatalytic performance of COFs in H_2_O_2_ generation and other related applications.

We comprehensively investigated the underlying mechanism of differences in photosynthetic ORR and WOR activities between the two COFs using density functional theory (DFT) calculations. The charge density differences of HZ-BTT-COF and TDA-BTT-COF are illustrated in Figure 1. Specifically, introducing the S atom altered the charge state of adjacent C/H moieties, leading to the local asymmetric electron distribution of TDA-BTT-COF. Furthermore, surficial electrostatic potential was measured by Kelvin probe force microscopy (KPFM), and the value of TDA-BTT-COF was found to be 104 mV, much higher than that of HZ-BTT-COF (54 mV) (Figure S33), which demonstrates that the built-in electric field is enhanced by the asymmetric electron distribution in TDA-BTT-COF. Moreover, the total charge numbers of different components in HZ-BTT-COF and TDA-BTT-COF were calculated, −0.24 |*e*| for HZ-BTT-COF and −0.30 |*e*| for TDA-BTT-COF, respectively, to evaluate their intramolecular polarities. Such enhanced intramolecular polarity boosted the electron donor–acceptor effect and facilitated the exciton dissociation in TDA-BTT-COF. As shown in Figure 5a, the Electron Localization Function (ELF) calculation exhibited a great distinction between HZ-BTT-COF and TDA-BTT-COF, where they showed only one and two electron channels along the covalent frameworks, respectively, indicating that TDA-BTT-COF exhibits more effective electron transfer than HZ-BTT-COF. Between them, the single electron channel on HZ-BTT-COF is BTT → N–N → benzene, but, contrarily, the two channels on TDA-BTT-COF are BTT → 1,2,4–thiadazole (C–N–N-C, C–S–C) → benzene, that is, TDA-BTT-COF possesses more efficient migration of electrons and holes.

**Figure 5:**
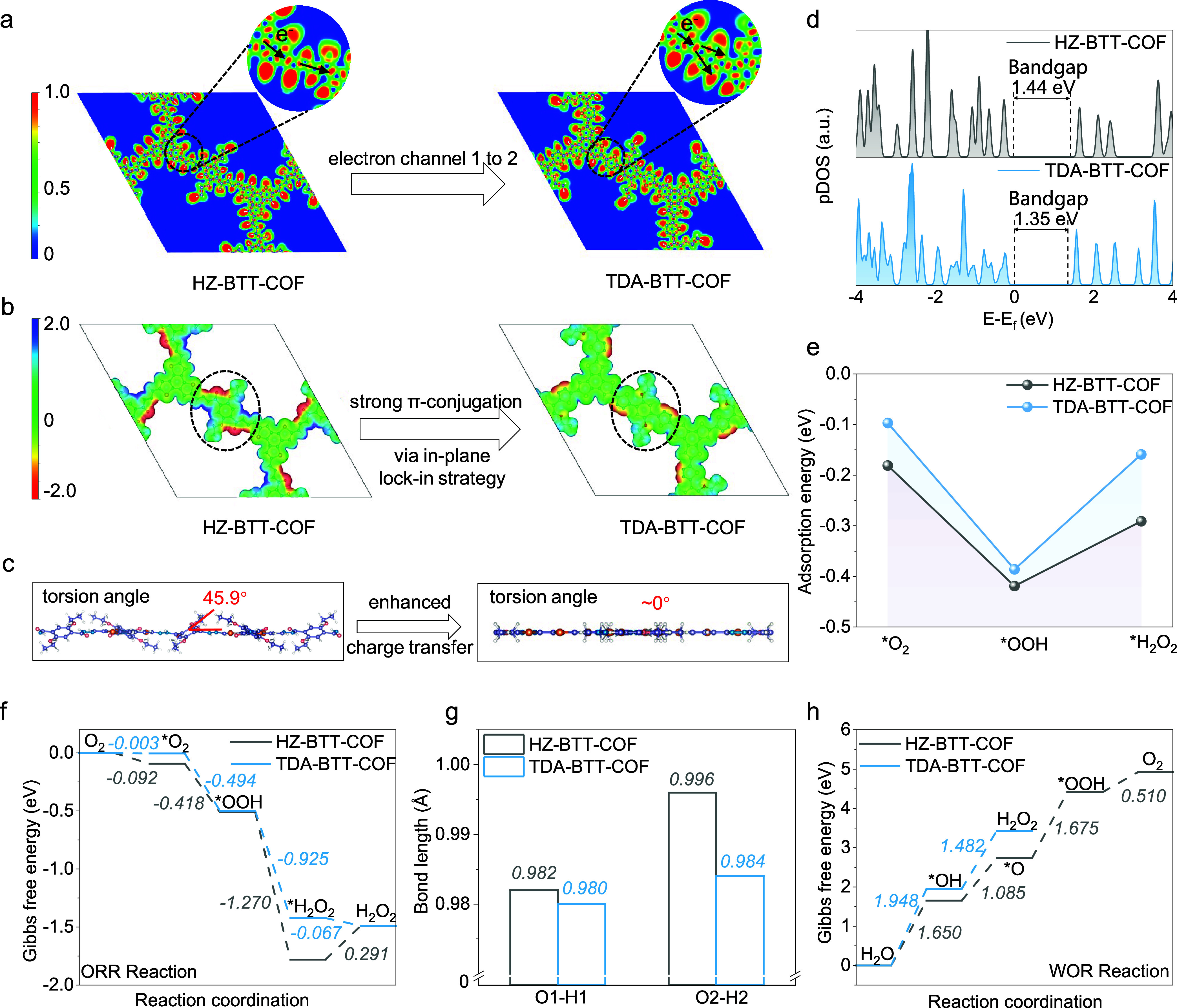
DFT calculations for investigating the H_2_O_2_ production mechanism. (a) Calculated Electron Localization Function (ELF) diagrams of HZ-BTT-COF and TDA-BTT-COF. (b) Electrostatic potential (ESP) analysis of HZ-BTT-COF and TDA-BTT-COF, ESP mapped surface charge density with the isosurface of 0.007 e·bohr^–3^. The color scale bar is shown at the left, while the corresponding ESP values (units of eV) range from −2.0 to 2.0. (c) The side view structures of HZ-BTT-COF and TDA-BTT-COF. (d) The calculated pDOS of HZ-BTT-COF (gray) and TDA-BTT-COF (blue). (e) The adsorption energy of intermediates during the ORR process for HZ-BTT-COF and TDA-BTT-COF. (f) The Gibbs free energy diagrams of HZ-BTT-COF and TDA-BTT-COF for H_2_O_2_ photogeneration by ORR. (g) The bond length of H_2_O_2_ adsorbed on HZ-BTT-COF and TDA-BTT-COF. (h) Gibbs free energy diagrams of HZ-BTT-COF and TDA-BTT-COF for H_2_O_2_ photogeneration by four-electron WOR process and two-electron WOR process, respectively.

To further explore the effect of cyclization, the electrostatic potential (ESP) of two COFs was calculated (Figure 5b), suggesting that the most distinguished part circled between them is their conjugation structure. Cyclization makes a stronger conjugation in TDA-BTT-COF. The side view structures of TDA-BTT-COF showed that the torsion angle in the basal plane is almost 0° after applying the π-conjugation lock-in strategy (Figure 5c). Based on this, projected density of state (pDOS) was conducted, revealing the bandgap of HZ-BTT-COF and TDA-BTT-COF as 1.44 and 1.35 eV (Figure 5d). Further, we used the Heyd-Scuseria-Ernzerhof (HSE06) hybrid functional to correct the band gap values obtained from pDOS calculations (Figure S34). HZ-BTT-COF has a band gap of 2.4 eV, and TDA-BTT-COF has a band gap of 2.3 eV, which are in close agreement with our experimental values of 2.51 and 2.31 eV, and are consistent with previous results. The discrepancy with the experimental values is attributed to the use of the Perdew–Burke–Ernzerhof (PBE) functional. Meanwhile, band structure calculations based on the PBE functional were performed for both HZ-BTT-COF and TDA-BTT-COF, and both materials exhibit direct band gap characteristics, which are consistent with the experimental results (Figure S35). Moreover, the spatial distribution of the conduction band minimum (CBM) and valence band maximum (VBM) reveals that both COFs exhibit a degree of charge separation, which reduces photoexcited electron–hole recombination, extends the lifetime of charge carriers, and ultimately improves photosynthetic efficiency (Figure S36). Notably, cyclization results in a more conjugated framework, thereby improving the intrinsic activity of TDA-BTT-COF.

There are two different reaction routes for H_2_O_2_ production: ORR and WOR reaction. To further understand the mechanism of photosynthetic H_2_O_2_ formation, we first calculated the optimized structures and adsorption energy (*E*_a_) of all the intermediates during ORR reaction on HZ-BTT-COF and TDA-BTT-COF (Figures 5e, S37 and 38). For the 2e^–^ ORR reaction, TDA-BTT-COF demonstrated a lower energy barrier for the third reduction step to produce *H_2_O_2_ and a much smaller Gibbs free energy (Δ*G*) for the rate determined step (RDS) H_2_O_2_ desorption than HZ-BTT-COF with the Δ*G* −0.067 eV for TDA-BTT-COF and 0.291 eV for HZ-BTT-COF (Figure 5f). Moreover, the ORR reaction of TDA-BTT-COF is spontaneous in the whole process and there is no significantly different ΔG in the first two steps *O_2_ and *OOH formation between TDA-BTT-COF and HZ-BTT-COF, which is −0.003 eV and −0.494 eV for TDA-BTT-COF and −0.092 eV and −0.418 eV for HZ-BTT-COF. Meanwhile, we calculated the changes in H_2_O_2_ bond lengths upon adsorption on both COFs. The results show that adsorbed H_2_O_2_ on HZ-BTT-COF exhibited a longer O–H bond length, indicating a stronger interaction between COF and H_2_O_2_ (Figure 5g). Combining with the adsorption energy and structures of all intermediates for ORR reaction, we found that TDA-BTT-COF possesses a weaker affinity for H_2_O_2_ and higher activity toward oxygen reduction than HZ-BTT-COF, and both of them have almost the same adsorbability of O_2_, because of the local asymmetric electron distribution leading to a weaker interaction with H_2_O_2_ after cyclization. Notably, throughout the process, adsorption of intermediates severely disrupts the conjugated structure of HZ-BTT-COF rather than TDA-BTT-COF, indicating that TDA-BTT-COF has the potential to transfer charges faster than HZ-BTT-COF. Furthermore, cyclization induces a transformation from C=O to C=S, significantly reducing the local polarity and electronegativity of the corresponding area, which in turn lowers the *E*_a_ of TDA-BTT-COF toward all intermediates.

Regarding to the WOR reaction, as shown in Figures 5h and S39–S41, the calculated ΔG during WOR reaction suggested HZ-BTT-COF had a high energy barrier of 1.780 eV in the second reaction step (*OH → H_2_O_2_) but rather lower energy barrier of further reaction *OH into *O (1.085 eV) and *OOH (0.510 eV) to produce O_2_, facilitating a 4e^–^ WOR instead of 2e^–^ WOR reaction.^60^ The bending structure of HZ-BTT-COF hinders charge migration and separation, leading to two outcomes: photoinduced holes becoming trapped within the bulk of the framework and undergoing rapid recombination, or accumulating on the surface, where they excessively oxidize water to produce O_2_. In addition, TDA-BTT-COF exhibits a Δ*G* for *OH at a thermodynamically favorable position, promoting efficient H_2_O_2_ formation. Nevertheless, excessively strong *OH bonding may facilitate further oxidation, thereby reducing the production efficiency (Figure S42). Therefore, TDA-BTT-COF possesses the potential to drive absorbed *OH efficiently to be combined to produce H_2_O_2_.^61^

From the above experimental and theoretical results, it could be proposed that HZ-BTT-COF undergoes a 2e^–^ ORR and 4e^–^ WOR to generate H_2_O_2_ with low photocatalytic activity and efficiency, whereas the overall H_2_O_2_ evolution over TDA-BTT-COF is via a 2e^–^ ORR and 2e^–^ WOR simultaneously. In conclusion, the local asymmetric electron distribution of TDA-BTT-COF enables a weaker affinity for H_2_O_2_ and higher activity toward oxygen reduction, which lowers the energy barrier of the ORR reaction and facilitates the progress of the reaction. Moreover, the effective D–A structure and the locked-in coplanar structure of TDA-BTT-COF enable rapid exciton dissociation along its extended π-conjugated domain, facilitating the generation of abundant free charge carriers. The photogenerated electrons follow a sequential two-electron pathway, with ^•^O_2_^–^ as intermediates, ultimately leading to H_2_O_2_ formation. Meanwhile, the corresponding holes become extensively delocalized and preferentially migrate along the surface of the 1,2,4-thiadiazole production. Together, these complementary redox processes constitute a complete catalytic cycle for the overall photosynthesis of H_2_O_2_, enabling efficient photocatalytic production.

### Photocatalytic Oxidative Coupling of Benzylamines

The photocatalytic performance of HZ-BTT-COF and TDA-BTT-COF was furthermore systematically evaluated for the aerobic oxidation of benzylamine in acetonitrile under mild conditions. Using benzylamine and its derivatives as model substrates, TDA-BTT-COF achieved complete conversion to *N*-benzylbenzaldimine within 1 h, whereas HZ-BTT-COF reached 72% conversion over the same period (Figure 6a). This significant enhancement in activity proves the beneficial role of thiadiazole incorporation in promoting photocatalytic efficiency. Kinetic analysis reveals that the oxidation reactions mediated by both COFs follow zero-order kinetics, indicating that the reaction rate is independent of substrate concentration under the conditions studied. In addition, we performed DFT calculations to study the adsorption structure and adsorption energies of benzylamine at five different sites on TDA-BTT-COF. We can clearly observe that the adsorption energy at the thiophene moiety (site 5) and the side-chain ether groups (site 2) is stronger than at other sites (Figure S43).

**Figure 6:**
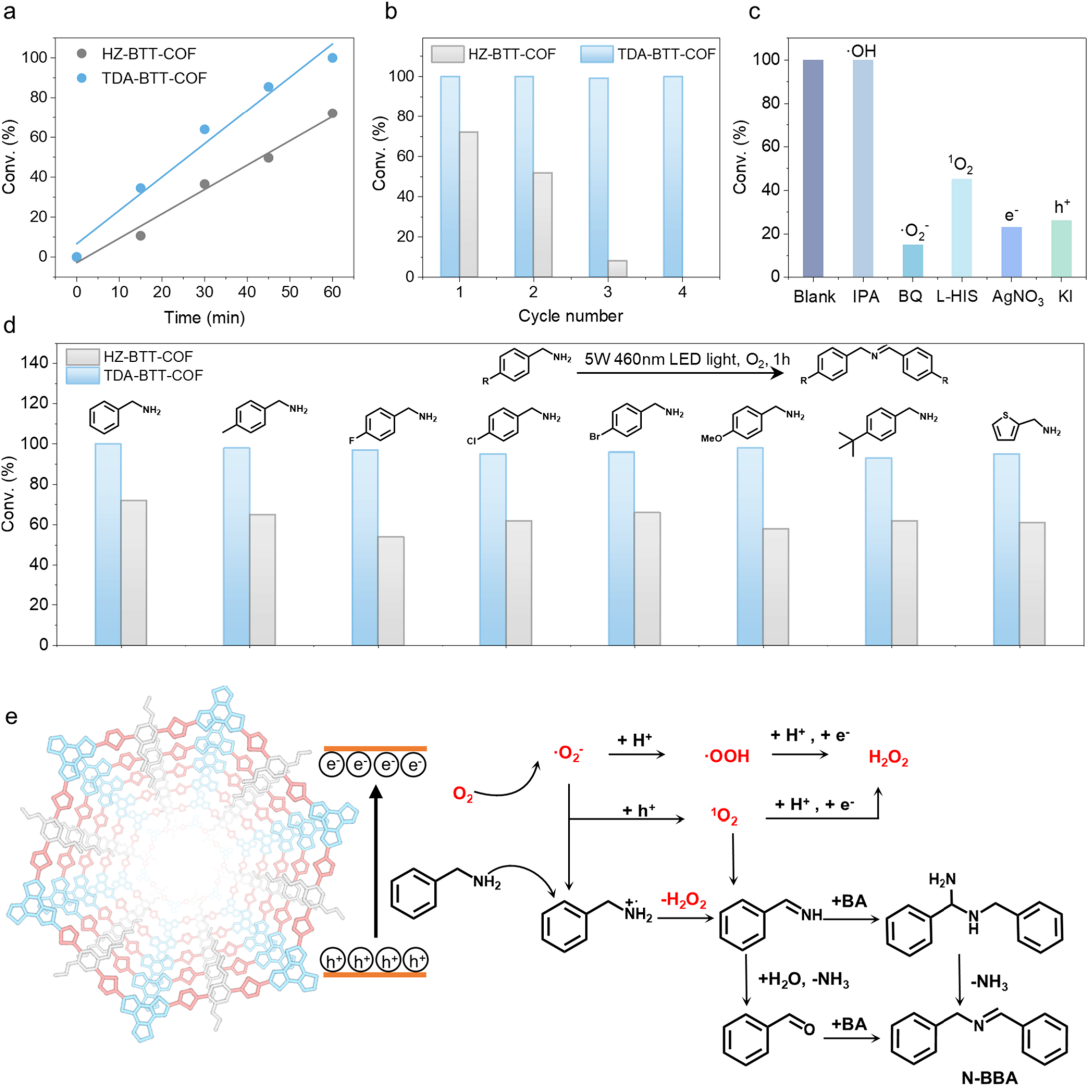
Photocatalytic performance toward the photocatalytic oxidative coupling of benzylamines. (a) Benzylamine conversion over time for HZ-BTT-COF and TDA-BTT-COF. (b) Reusability of HZ-BTT-COF and TDA-BTT-COF. Reaction conditions: photocatalyst (8 mg), benzylamine (0.2 mmol), blue LEDs (460 nm, 5 W), CH_3_CN (2 mL), O_2_ (1 atm), and 1 h. (c) Control experiments for photocatalytic oxidative coupling of benzylamine by TDA-BTT-COF under normal conditions or with different scavengers. (d) Photocatalytic performances of HZ-BTT-COF and TDA-BTT-COF in the coupling of benzylamines. (e) Proposed coupling reaction mechanism of benzylamine (BA: benzylamine, N-BBA: *N*-benzylbenzaldimine).

The recyclability of TDA-BTT-COF in the benzylamine coupling reaction mirrored the stability trends observed in previous tests: the material retained high photocatalytic activity across four consecutive cycles, whereas HZ-BTT-COF suffered substantial deactivation (Figure 6b). PXRD of the recovered TDA-BTT-COF confirmed preservation of its crystalline structure after repeated use (Figure S44), and FTIR spectroscopy revealed no detectable chemical degradation, with postreaction spectra matching those of the as-synthesized material (Figure S45). These findings affirm the robust stability of the thiadiazole-linked framework and validate its suitability as a recyclable photocatalyst even under demanding reaction conditions.

We further investigated the photocatalytic performance of HZ-BTT-COF and TDA-BTT-COF by extending the substrate scope to various substituted benzylamines. Under standardized reaction conditions, TDA-BTT-COF consistently afforded higher product yields than its hydrazone-linked counterpart across all substrates tested (Figures 6d, S46–S53 and Table S4), indicating that the thiadiazole linkage enhances not only structural robustness but also photocatalytic efficiency. Control experiments demonstrated that the reaction requires the simultaneous presence of blue LED illumination, molecular oxygen, and TDA-BTT-COF, as the omission of any component resulted in minimal product formation (Table S5).

A series of ROS quenching experiments was performed to evaluate the predominant ROS intermediates in the oxidation process (Figure 6c). The addition of isopropanol leads to a negligible decline in yield, indicating that ^•^OH is not involved in this photocatalytic process. When l-histidine (l-HIS) or p-benzoquinone (BQ) are added as ^1^O_2_ and ^•^O_2_^–^ scavengers, sharp declines in conversion from 100% to 45% and 15% are observed. Therefore, ^1^O_2_ and ^•^O_2_^–^ are key radicals in this reaction. The addition of AgNO_3_ alongside O_2_ as an acceptor of photogenerated electrons (e^–^) inhibits the formation of the desired product. The utilization of KI as a hole (h^+^) scavenger leads to an *N*-benzylbenzaldimine conversion of just 18%. Thus, the e^–^ and h^+^ also play significant roles in this selective coupling reaction. To verify the generation of the above-mentioned ROS, EPR spectroscopy in CH_3_CN of TDA-BTT-COF was performed. As shown in Figure S54, under an oxygen atmosphere, there is no apparent signal under dark conditions. After 5 min of illumination, the ^•^O_2_^–^ signals appeared in the system with the presence of DMPO, indicating that TDA-BTT-COF could rapidly activate oxygen molecules to superoxide radicals under light irradiation. ^1^O_2_ was also detected in the presence of 2,2,6,6-Tetramethyl-4-piperidone (TEMP). However, no singlet oxygen was detected in HZ-BTT-COF, which may be the main reason the efficiency of benzylamine coupling catalyzed by HZ-BTT-COF is lower than that catalyzed by TDA-BTT-COF. According to the obtained results, the reaction mechanism for the photocatalytic coupling of benzylamine is shown in Figure 6e.

Furthermore, the proposed lock-in reinforcement combined with the asymmetric electron distribution strategy is a universal modification strategy. Here, two additional types of COF were prepared to demonstrate that the sulfur addition reaction can also convert the hydrazone linkages to thiadiazole linkages in other COFs, thereby enhancing their photocatalytic performances. For instance, hydrazone-linked triazine COF (HZ-TTA-COF) and hydrazone-linked triformylbenzene COF (HZ-TFB-COF) were synthesized via solvothermal reactions, followed by the preparation of TDA-TTA-COF and TDA-TFB-COF (thiadiazole-linked triazine COF and thiadiazole-linked triformylbenzene COF) under similar conditions as those of TDA-BTT-COF (Figure 7a and see Supporting Information). PXRD, FTIR, and ^13^C ssNMR analyses confirmed the successful conversion of hydrazone to thiadiazole linkages (Figures S55–58 and 7b,c). Both TDA-TTA-COF and TDA-TFB-COF showed good performance in photocatalytic H_2_O_2_ production and photocatalytic performance toward the oxidative coupling of benzylamines, even though lower values were obtained compared to TDA-BTT-COF (in the order TDA-BTT-COF > TDA-TTA-COF > TDA-TFB-COF) (Figure 7d,e). This result highlights the substantial improvement in photocatalytic performance achieved through the formation of thiadiazole bonds.

**Figure 7:**
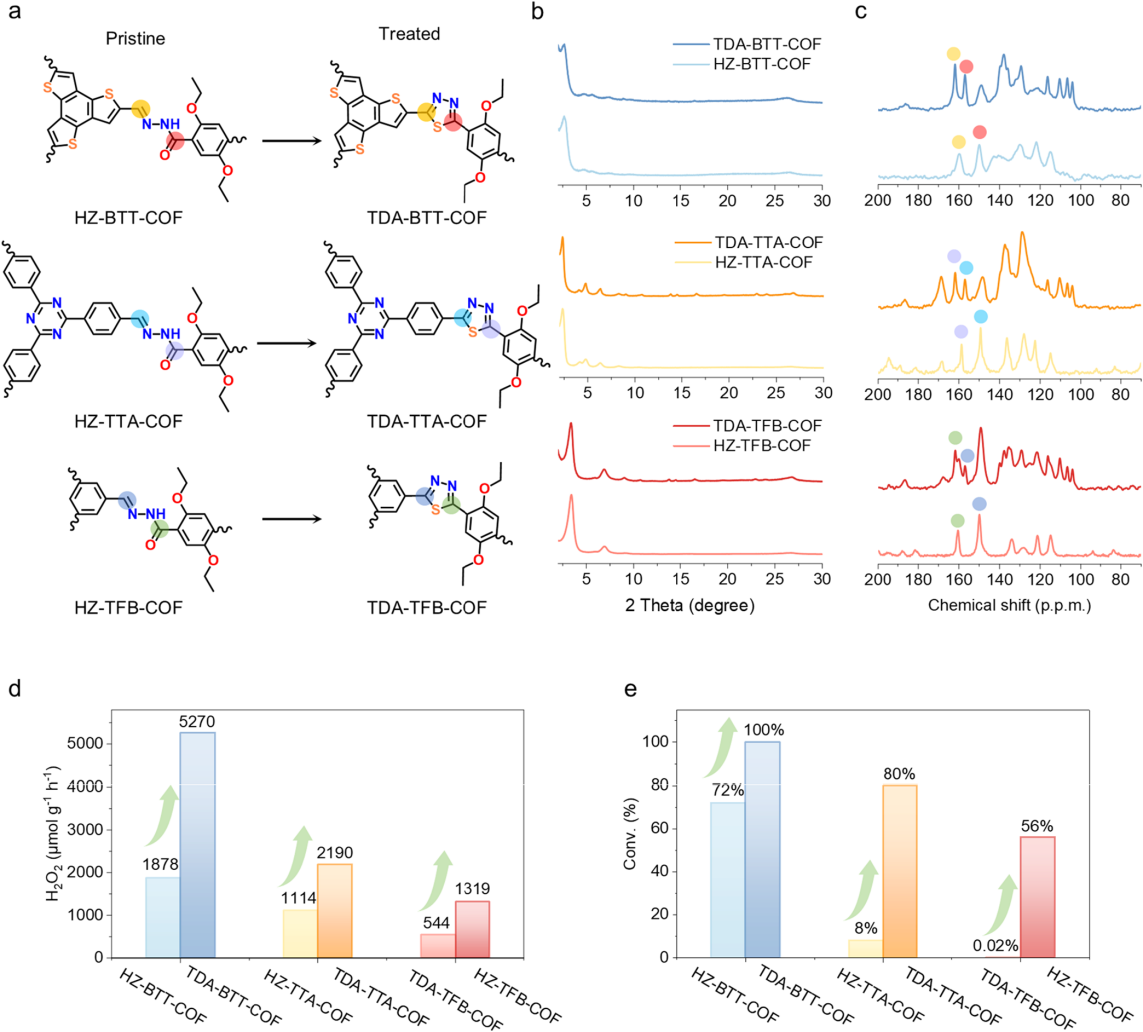
The universality of the proposed lock-in reinforcement strategy for enhancing other COF-based photocatalysts. (a) Scheme of the preparation of TDA-COFs via linkage conversion from hydrazone to thiadiazole. (b) PXRD pattern of six COFs. (c) ^13^C ssNMR of six COFs. (d) Photocatalytic activity of six COFs for H_2_O_2_ production. (e) Benzylamine conversion for six different COFs.

## Conclusions

In this work, we present a lock-in reinforcement strategy integrated with asymmetric electron distribution to construct thiadiazole-based sulfur-heteropolycyclic COFs through a postcyclization synthetic route. The resulting TDA-BTT-COF demonstrates exceptional performance in photocatalytic H_2_O_2_ generation and aerobic benzylamine oxidation, attributable to a synergistic combination of structural and electronic advantages. The incorporation of sulfur heteroatoms into thiadiazole units establishes localized asymmetric electron density, while the heteroaromatic architecture enhances intramolecular polarity, strengthens donor–acceptor interactions, and promotes efficient exciton dissociation. Moreover, the locked-in coplanar conformation enables rapid charge-carrier migration along an extended π-conjugated pathway, and the robust heterocyclic and heteropolycyclic framework confers remarkable chemical stability, thereby collectively elevating the intrinsic photocatalytic activity beyond that of conventional COF designs.

These structural merits translate into outstanding performance, with TDA-BTT-COF achieving a photosynthetic H_2_O_2_ production rate of 5270 μmol g^–1^ h^–1^ in pure water, representing a 3-fold enhancement over the hydrazone-linked precursor and surpassing most reported organic and inorganic competitors. The material also exhibits complete benzylamine conversion within 1 h under mild blue LED irradiation, highlighting its efficacy in oxidative coupling transformations. Beyond these specific reactions, the methodology established here provides a general platform for designing stable, highly conjugated organic frameworks with tailored optoelectronic properties. The ability to precisely engineer charge distribution, conjugation length, and catalytic stability through rational heteroatom integration opens new avenues for developing advanced materials for photocatalytic and electrocatalytic applications, as well as for organic electronics, sensing, and sustainable chemical synthesis. This work thus bridges molecular design, structural control, and functional performance, offering a scalable pathway to sophisticated organic materials for energy conversion and green chemical technologies.

## Supplementary Material



## Notes

Experimental details, including materials, characterizations, synthesis of photocatalyst, theoretical calculations, and H_2_O_2_ detection method (PDF)
