# Supplementary material for: Conjugation Lock-In Reinforced Sulfur-Heteropolycyclic Covalent Organic Frameworks with Asymmetric Electron Distribution for Photocatalytic Aerobic Oxidation Reactions
Source: J Am Chem Soc. 2026 Mar 9;148(10):10945–59. doi: 10.1021/jacs.5c21545 (PMC13003510; doi:10.1021/jacs.5c21545)
Supplement: Supplementary file 1 [file ja5c21545_si_001.pdf]

Supporting Information for

Conjugation Lock-In Reinforced Sulfur-Heteropolycyclic Covalent Organic Frameworks with Asymmetric Electron Distribution for Photocatalytic Aerobic Oxidation Reactions

Jiani Yang,[1,§] Shihuan Gao,[1,§] Zhenyang Zhao,[1] Xiaohui Xu,[2] Siyuan Liu,[3] Min Xu,[4] Weichao Xue,[4] Fan Dong,[3] Shuang Li,[1,*] Arne Thomas,[5,*] and Chong Cheng[1,6,*]

AUTHOR ADDRESS.

[1] College of Polymer Science and Engineering, State Key Laboratory of Advanced Polymer Materials, Sichuan University, Chengdu, 610065, China

[2] Department of Ultrasound, Frontiers Science Center for Disease-Related Molecular Network, West China Hospital, Sichuan University, Chengdu, 610041, China

[3] Research Center for Carbon-Neutral Environmental & Energy Technology, Institute of Fundamental and Frontier Sciences, University of Electronic Science and Technology of China, Chengdu, 611731, China

[4] College of Chemistry, Sichuan University, Chengdu, 610065, China

[5] Functional Materials, Department of Chemistry, Technische Universität Berlin, Hardenbergstraße 40, Berlin, 10623, Germany

[6] Department of Endodontics, State Key Laboratory of Oral Diseases, National Center for Stomatology, West China Hospital of Stomatology, Sichuan University, Chengdu, 610041, China

[§] These authors contributed equally to this work.

This PDF file includes:

Experimental Section

Figs. S1 to S47

Tables S1 to S5

References (1 to 24)

Experimental Section

Materials and Reagents

All solvents and reagents obtained from commercial sources were used without further purification. Anhydrous dioxane (99%, AR), mesitylene (99%, AR), acetic acid (>99.0%), 1,2-dichlorobenzene (o-DCB), 1,3-dimethyl-2-imidazolidinone (DMI), 1,8-Diazabicyclo[5.4.0]undec-7-ene(DBU), anhydrous tetrahydrofuran (THF), anhydrous acetone, Lawesson's reagent, 4-(dimethylamino)pyridine, N, N-dimethylformamide(DMF), benzyl alcohol (BA), t-butyl alcohol (TBA), methanol (MeOH, AR), and ethanol (EtOH, AR) were obtained from Aladdin. Benzo[1,2-b:3,4-b':5,6-b'']trithiophene-2,5,8-tricarbaldehyde (BTT), 1,3,5-triformylbenzene (TFB), 1,3,5-tris(4-formyl-phenyl)triazine (TTA), and 2,5-diethoxyterephthalohydrazide (DETH) were purchased from Jilin Chinese Academy of Sciences-Yanshen Technology Co., Ltd.

Structural Characterization

Scanning electron microscopy (SEM) was performed with HITACHI Regulus 8220. ^{13}C solid-state NMR (cross-polarization magic-angle spinning (CP/MAS)) spectra were carried out on a Bruker Avance III 500 MHz spectrometer operating at 100.6 MHz. A Nicolet iS50 spectrophotometer (Nicol, US) was used for Fourier transform infrared (FTIR) spectroscopy. Transmission electron microscopy (TEM), high-resolution TEM (HRTEM), and elemental mapping were performed using a Talos F200x TEM microscope (FEI Ltd., USA) operated at 200 kV and analyzed using GMS-free analysis. Powder X-ray diffraction (PXRD) was performed on a DX-2700BH multipurpose X-ray diffractometer (XRD, Haoyuan Instrument, Liaoning, China) using Cu radiation at 40 kV over a 2θ range of 2-30°. The N_2 adsorption/desorption isotherms are obtained using a Quantachrome instrument and analyzed using the Brunauer-Emmett-Teller (BET) method to assess surface area and pore-size distribution, respectively. X-ray photoelectron spectroscopy (XPS) spectra were measured using the K-Alpha™ + X-ray Photoelectron Spectrometer System (Thermo Scientific), equipped with a Hemispherical 180° dual-focus analyzer and a 128-channel detector. Thermogravimetric analysis was performed using a Mettler TGA/DSC 3+ thermogravimetric analyzer. The pH value is determined by a PHS-2F pH meter from INESA Scientific Instrument Co., Ltd. The photoluminescence (PL) tests were recorded on a Fluorolog-3 spectrophotometer at emission scan mode. The electron paramagnetic resonance EPR spectra were recorded on a Bruker EMXplus spectrometer. The products of the aerobic oxidation reaction were detected and analyzed using a gas chromatography-mass spectrometer (GC–MS,

Clarus 690, PerkinElmer) and a gas chromatograph (GC-2010 Pro, Shimadzu).

Synthesis and Characterization of COFs

The synthesis of HZ-BTT-COF: The HZ-BTT-COF was synthesized according to the literature methods. Benzo[1,2-b:3,4-b':5,6-b'']trithiophene-2,5,8-tricarbaldehyde (20.0 mg, 0.06 mmol) and 2,5-diethoxyterephthalohydrazide (25.7 mg, 0.09 mmol) were added to a Pyrex tube. After 1,2-dichlorobenzene (1.3 mL), 1,3-dimethyl-2-imidazolidinone (0.86 mL), and 6 M aqueous acetic acid (0.125 mL) were added, the resultant mixture was ultrasonicated for 10 min, and then degassed through three freeze-pump-thaw cycles, sealed under vacuum. After warming to room temperature, the sealed tube was heated to 180 °C and left undisturbed for 3 days. The solid was isolated by filtration and washed with anhydrous tetrahydrofuran and anhydrous acetone. HZ-BTT-COF was obtained after drying under vacuum at 60 °C for 12 h (41.2 mg, 97%).

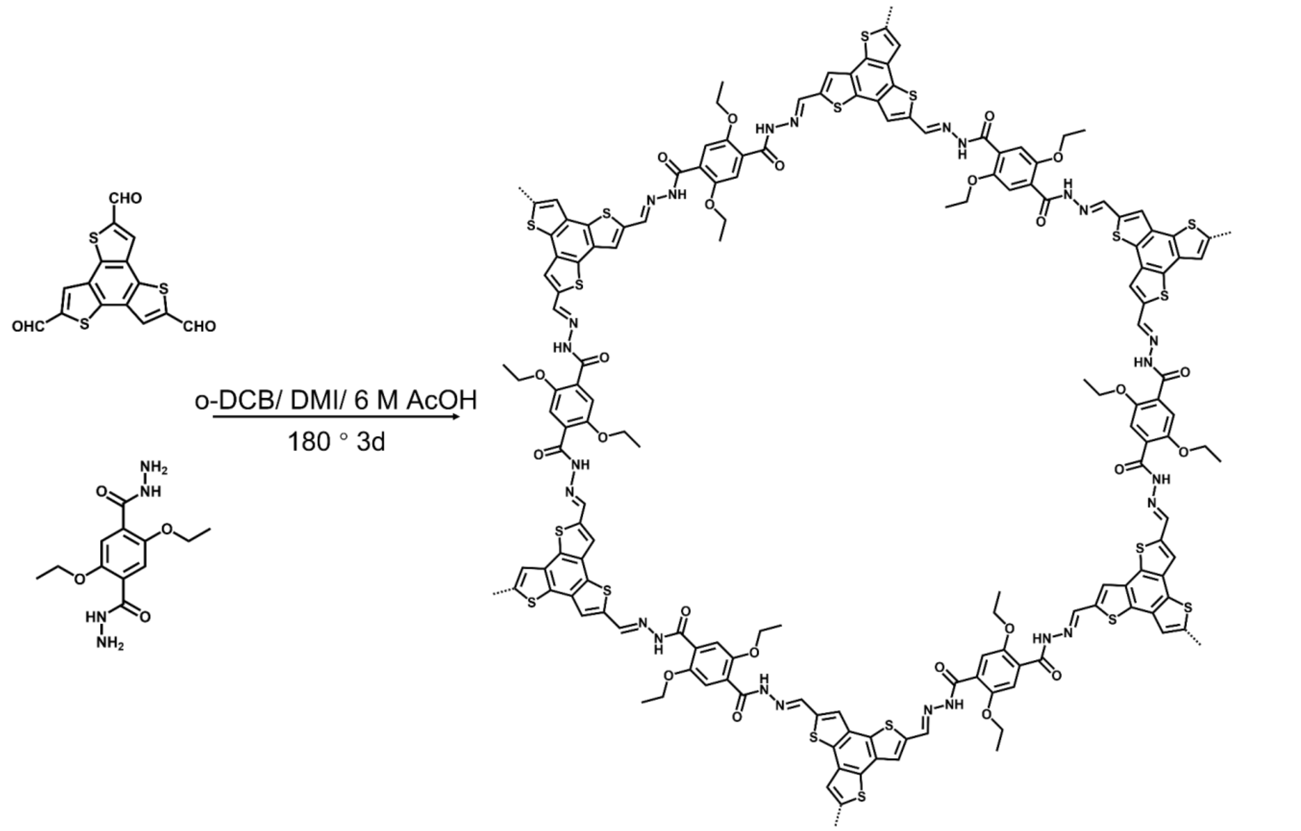

Scheme S1. Solvothermal synthesis of HZ-BTT-COF.

The synthesis of HZ-TTA-COF: The HZ-TTA-COF was synthesized according to the literature methods.

1,3,5-tris-(4-formyl-phenyl)triazine (TTA) (39.3 mg, 0.1 mmol) and 2,5-diethoxyterephthalohydrazide (42.5 mg, 0.15 mmol) were added to a Pyrex tube. After mesitylene (3 mL), anhydrous dioxane (1 mL), and 6 M aqueous acetic acid (0.4 mL) were added, the resultant mixture was ultrasonicated for 10 min, and then degassed through three freeze-pump-thaw cycles, sealed under vacuum. After warming to room temperature, the sealed tube was heated at 120 °C and left undisturbed for 3 days. The solid was isolated by filtration and washed with anhydrous tetrahydrofuran and anhydrous acetone. HZ-TTA-COF was obtained after drying under vacuum at 60 °C for 12 h (74.5 mg, 98%).

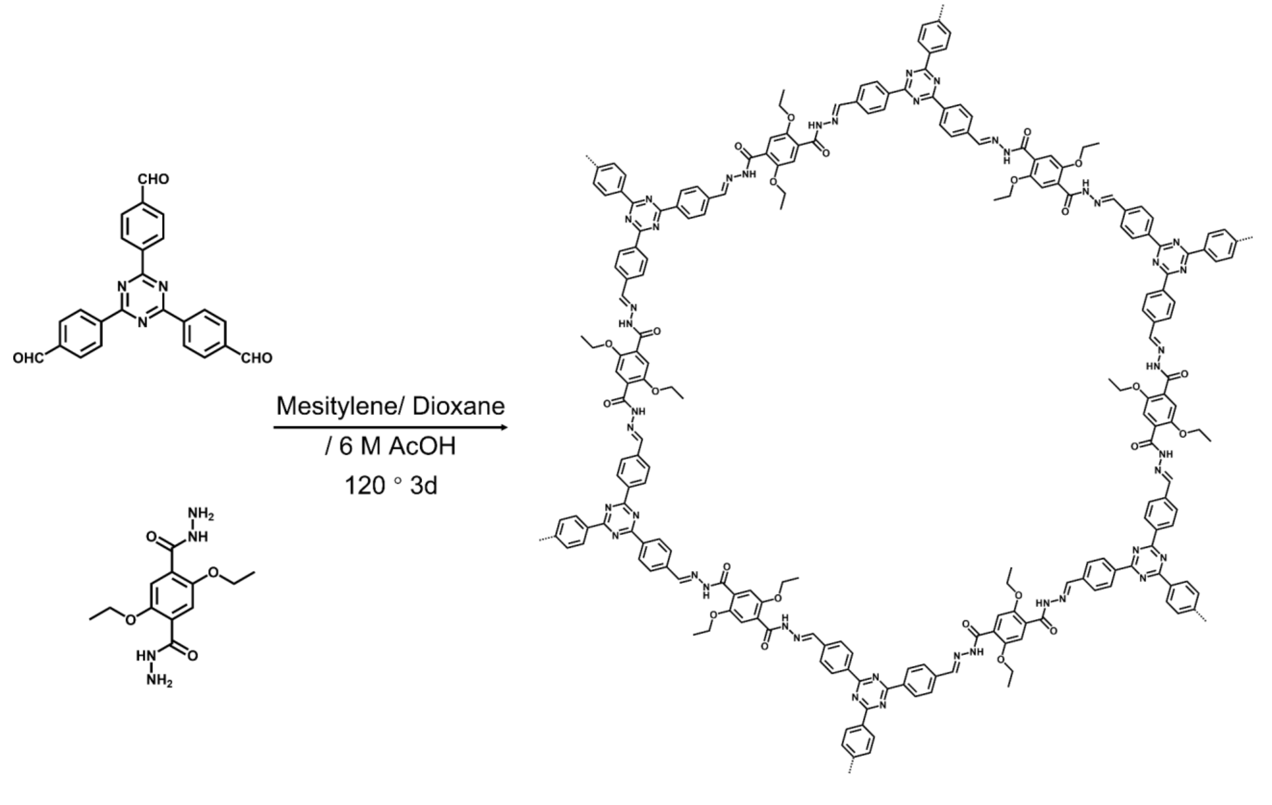

Scheme S2. Solvothermal synthesis of HZ-TTA-COF.

The synthesis of HZ-TFB-COF: The HZ-TFB-COF was synthesized according to the literature methods. 1,3,5-Triformylbenzene (TFB) (16.2 mg, 0.1 mmol) and 2,5-diethoxyterephthalohydrazide (42.5 mg, 0.15 mmol) were added to a Pyrex tube. After mesitylene (3 mL), anhydrous dioxane (1 mL), and 6 M aqueous acetic acid (0.4 mL) were added, the resultant mixture was ultrasonicated for 10 min, and then degassed through three freeze-pump-thaw cycles, sealed under vacuum. After being warmed to room temperature, the sealed tube was heated at 120°C and left undisturbed for 3 days. The solid was isolated by filtration and washed with anhydrous tetrahydrofuran and anhydrous acetone. HZ-TFB-COF was obtained after drying under

vacuum at 60 °C for 12 h (51.4mg, 96%).

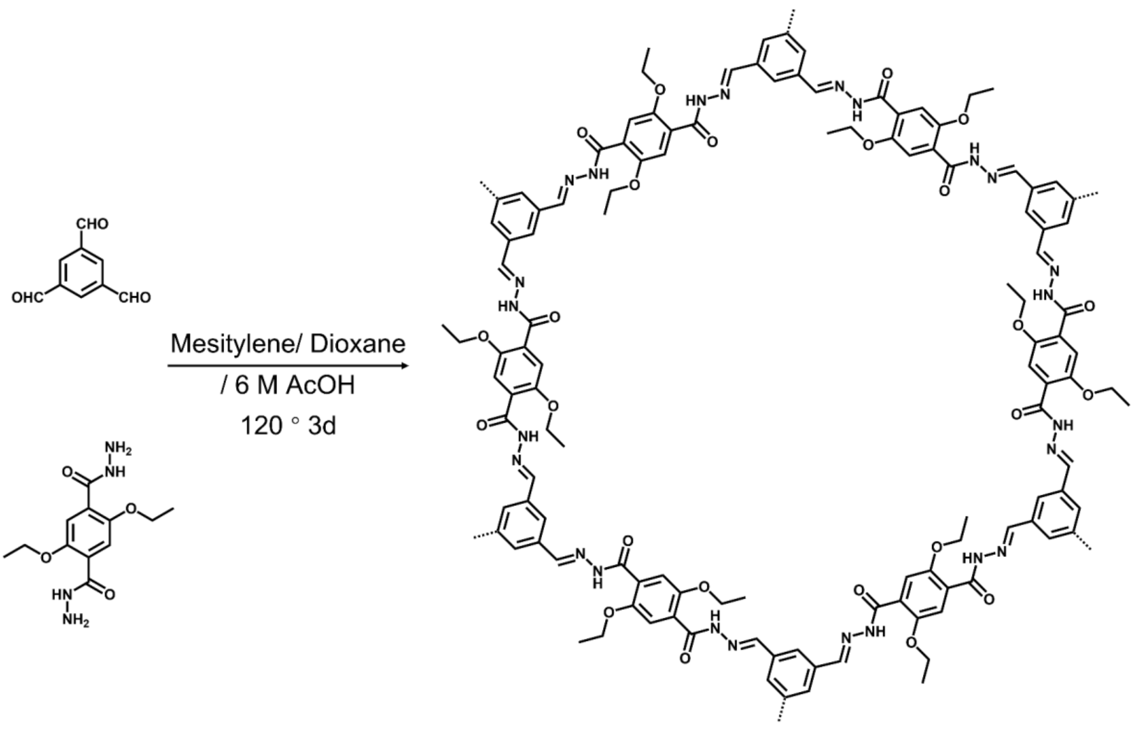

Scheme S3. Solvothermal synthesis of HZ-TTA-COF.

The synthesis of model compound: In a round-bottom flask, benzaldehyde (1.0 mmol) and benzoyl hydrazine (1.0 mmol) were added to ethanol (5.0 ml) at room temperature. The reaction mixture was refluxed for 2 h. The ethanol was evaporated in vacuo. To the resulting crude product, Lawesson's reagent (0.8 mmol, 0.8 eq) and toluene (10 ml) were added, followed by 4-(dimethylamino)pyridine (1.2 mmol, 1.2 eq). The resulting mixture was refluxed for 10 to 15 h until 2,5-diphenyl-2,3-dihydro-1,3,4-thiadiazole was not detected by TLC. After toluene evaporation, the residue was purified by flash column chromatography[1].

The synthesis of TDA-BTT-COF: The TDA-BTT-COF was synthesized according to the literature methods[2]. To the HZ-BTT-COF (40 mg), Lawesson's reagent (100 mg), and toluene (20 mL) were added, followed by 4-(dimethylamino)pyridine (44 mg). The resulting mixture was refluxed for 24 h. The resultant solid was isolated by filtration, washed with DMF and THF, and then dried under vacuum at 70 °C. Next, the solid was redispersed in 20 mL toluene, followed by the addition of the same amount of Lawesson's reagent and 4-(dimethylamino)pyridine, and the mixture was refluxed for 24 h. The resultant solid was isolated by filtration, washed with DMF and THF, and then dried under vacuum at 70 °C (38 mg, 95%).

The synthesis of TDA-TTA-COF: To the HZ-TTA-COF (40 mg), Lawesson's reagent (100 mg), and toluene (20 mL) were added, followed by 4-(dimethylamino)pyridine (44 mg). The resulting mixture was refluxed for 24 h. The resultant solid was isolated by filtration, washed with DMF and THF, and then dried under vacuum at 70 °C. The above steps are repeated twice, resulting in a total reaction time of 72 h (37 mg, 93%).

The synthesis of TDA-TFB-COF: To the HZ-TTA-COF (40 mg), Lawesson's reagent (100 mg), and toluene (20 mL) were added, followed by 4-(dimethylamino)pyridine (44 mg). The resulting mixture was refluxed for 24 h. The resultant solid was isolated by filtration, washed with DMF and THF, and then dried under vacuum at 70 °C. The above steps are repeated twice, resulting in a total reaction time of 72 h (38 mg, 95%).

The synthesis of sp^2-BTT-COF: 2,2'-(1,4-Phenylene)diacetonitrile (23.4 mg, 0.15 mmol) and benzo[1,2-b:3,4-b':5,6-b''] trithiophene-2,5,8-tricarbaldehyde (33 mg, 0.1 mmol) were placed into a 20 mL Pyrex tube, and dissolved into 1,2-dichlorobenzene (2 mL) and n-butyl alcohol (0.5 mL) mixed solution (v/v = 4:1). After the above mixture was sonicated for 10 min, DBU (0.2 mL) was added, and then the system was sonicated again for 5 min. The tube was degassed by three freeze-pump-thaw cycles, sealed, and heated to 120 °C for 3 days. The collected powder was washed with dimethylacetamide, ethyl alcohol, and tetrahydrofuran several times, and Soxhlet extraction was performed with THF. Finally, the derived precipitate was dried at 80 °C under vacuum for 24 h, which gives deep red sp^2-BTT-COF powder.

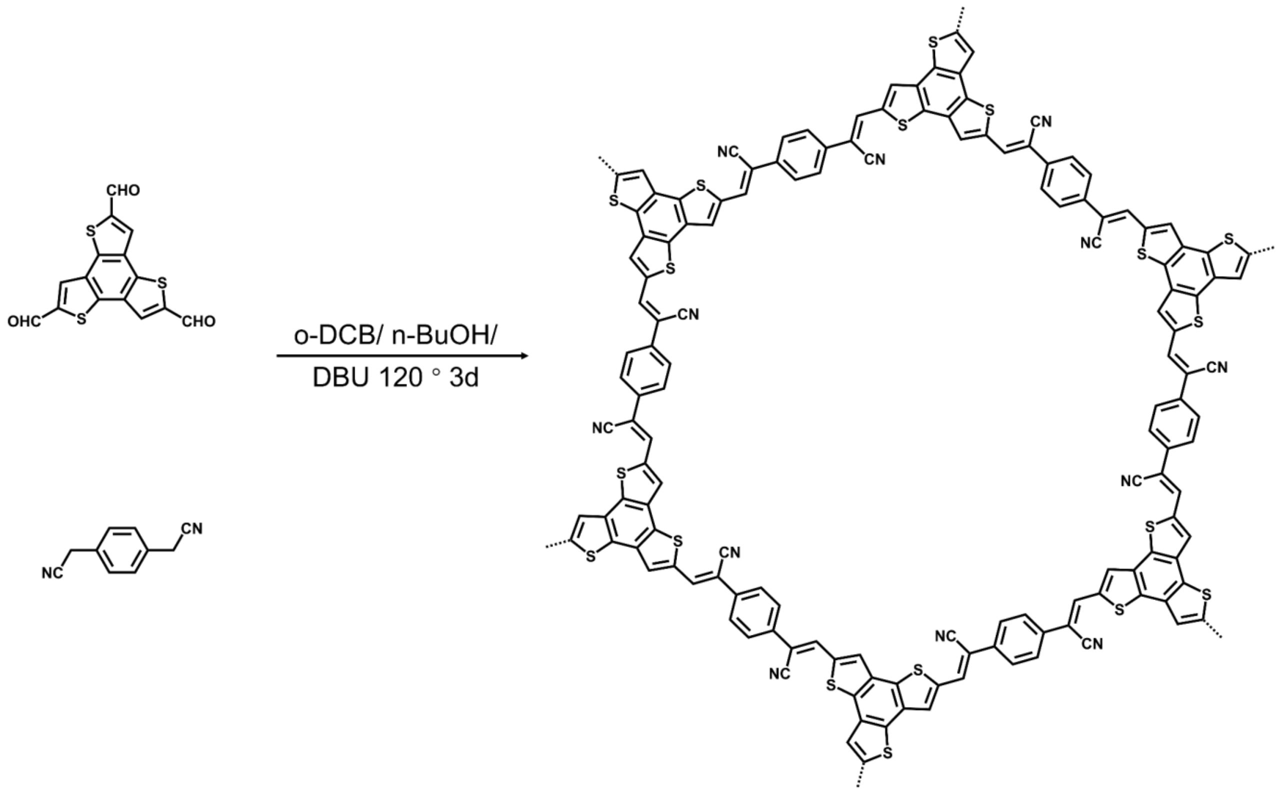

Scheme S3. Solvothermal synthesis of sp^2-BTT-COF.

The synthesis of im-BTT-COF: p-Phenylenediamine (16.2 mg, 0. 15 mmol) and benzo[1,2-b:3,4-b':5,6-b'']trithiophene-2,5,8-tricarbaldehyde (33 mg, 0.1 mmol) were placed into a 10 mL Pyrex tube, and dissolved into 1,2-dichlorobenzene (1.5 mL) and n-butyl alcohol (1.5 mL) mixed solution (v/v = 1:1). After sonication of the above mixture for 10 min, 6 M acetic acid (0.1 mL) was added and then sonicated again for 5 min. The tubes were degassed by three freeze-pump-thaw cycles, then sealed and heated at 120 °C for 3 days. The collected powder was washed with ethanol, acetone, and tetrahydrofuran several times, and Soxhlet extraction was performed with THF. Finally, the derived precipitate was dried at 80 ℃ under vacuum for 24 h, which gives orange im-BTT-COF powder.

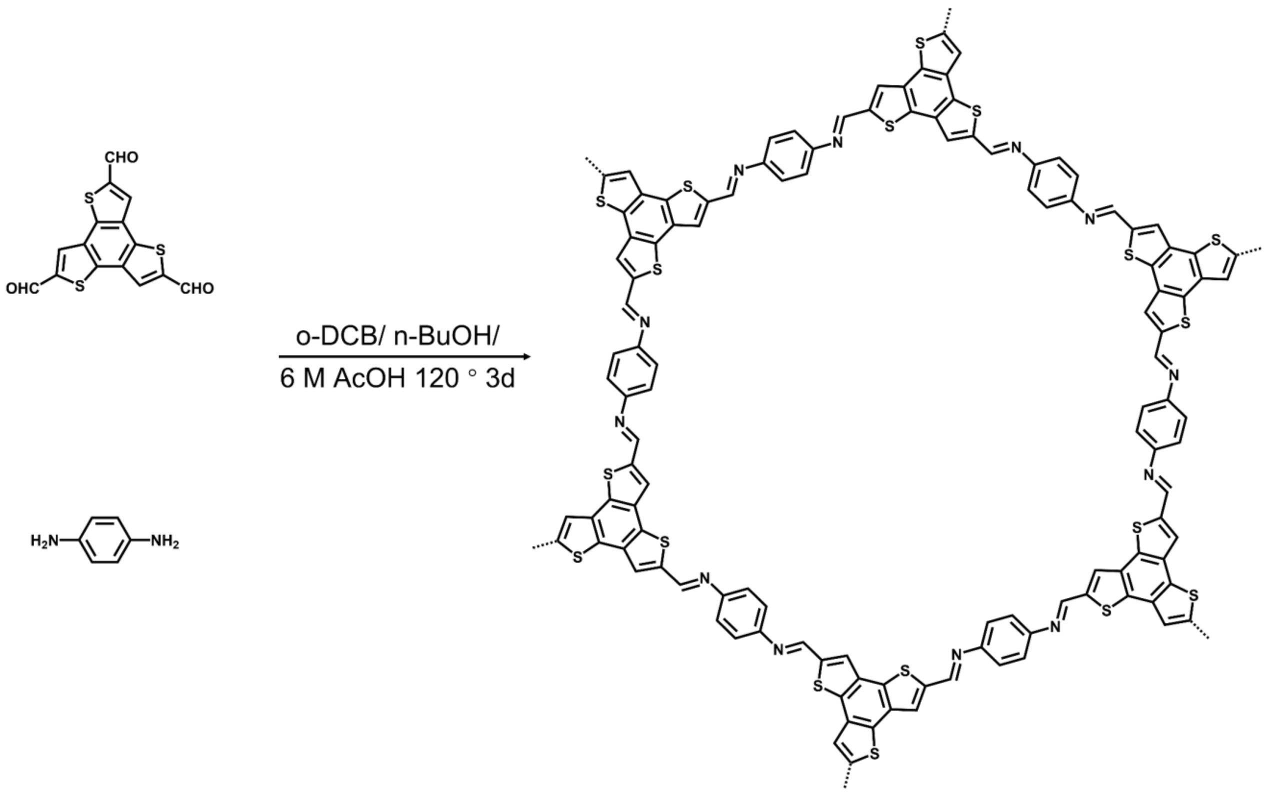

Scheme S4. Solvothermal synthesis of im-BTT-COF.

H_2O_2 detection method (Iodometry): The amount of H_2O_2 was analyzed by Iodometry. 1 mL of 0.1 mol·L^{-1} potassium hydrogen phthalate ($C_8H_5KO_4$) aqueous solution and 1 mL of 0.4 mol·L^{-1} potassium iodide (KI) aqueous solution were added to 0.2 mL solution that was taken out from the catalytic system; the mixture was then kept for 30 min. H_2O_2 molecules react with iodine ions (I^-) under acidic conditions to generate triiodide ions (I^{3-}), which have strong absorption near 350 nm. The absorbance of I^{3-} at 350 nm is measured by an ultraviolet spectrophotometer (UV-6100S), and then the amount of H_2O_2 generated by the photocatalytic reaction can be calculated.

Photocatalytic H_2O_2 production: The experiment was performed in a 50 mL vial containing COFs (5 mg) and ultrapure water (25 mL). The suspension was stirred in the dark for 10 min, followed by oxygen bubbling for 20 min. The reaction system was irradiated with a xenon lamp source (CEL-HXF300-T3, China Education Au-light, $\lambda > 420$ nm, with circulating water), and O_2 was continually bubbled into the vial. The concentration of H_2O_2 was determined by the UV-Vis spectrophotometer.

H_2O_2 decomposition: Taking into account the acceleration of H_2O_2 decomposition under irradiation, the stability of the produced H_2O_2 was assessed by measuring the degradation behavior of H_2O_2 generated during the reaction of the prepared samples. The mixed solution of H_2O_2 (1 mM, 20 mL) and photocatalyst (0.5 mg mL^{-1}) was sonicated for 2 minutes, followed by purging the system with argon. Subsequently, the light source was turned on, and the residual H_2O_2 concentration was measured every 15 minutes to assess its stability.

Photocatalytic oxidative coupling of benzylamines: The photocatalytic experiment is as follows: the photocatalyst (8 mg) was dispersed in 2 mL of CH_3CN containing 0.2 mmol benzylamine in a quartz reactor (10 mL). After that, the mixture was bubbled with O_2 for 10 minutes, and the photoreactor was sealed. A light-emitting diode (LED) light (total 5 W) was used as the light source, accompanied by an O_2 balloon. The reaction temperature was controlled at 298 K by using the cooling water circulation. After the reaction, the solution was collected, centrifuged, and filtered through a 0.22 μm syringe filter to remove catalyst particles. The products in the filtrate were identified by GC (GC-2010 Pro AF) with a flame ionization detector (FID), and the conversion and selectivity were also analyzed by GC. Other benzylamine derivatives were tested with the same concentration and conducted using a similar approach.

Photoelectrochemical measurements: Electrochemical characterizations, including Mott–Schottky analysis, photocurrent response, and Electrochemical impedance spectroscopy (EIS), were conducted using a Gamry Reference 600 workstation (USA) configured with a standard three-electrode cell. For working electrode fabrication, 10 mg of catalyst was ultrasonically dispersed in a mixture of 0.9 mL ethanol/0.1 mL Nafion to form a homogeneous ink. Subsequently, 50 μL of this slurry was uniformly coated onto ITO glass (1×1 cm^2, loading: 0.5 mg/cm^2) and infrared-dried. The three-electrode configuration employed a platinum wire counter electrode and an Ag/AgCl reference electrode. Before measurements, the Ag/AgCl electrode was RHE-calibrated in H_2-saturated electrolyte using a Pt wire, with all ORR potentials subsequently referenced to RHE. All electrolytes tested were 0.5 M Na_2SO_4, which was freshly prepared (within two weeks) and stored in a sealed container at room temperature (25 ± 2 °C). The specific configuration scheme involves dissolving 35.5 g of Na_2SO_4 in 500 mL of ultrapure water to yield 0.5 M Na_2SO_4. The solution resistance was measured at 16.9 ± 0.5 Ω. All measurements were automatically corrected for iR drop using real-time resistance compensation.

EIS analysis was performed under dark conditions (frequency: 100 kHz–0.01 Hz, bias: 0 V) to determine charge transfer resistance (Rct). Mott–Schottky measurements spanned –1 to 1 V at 500, 1000, and 1500 Hz. Photocurrent testing employed a 300 W Xenon lamp (λ > 420 nm; CEL-HXF300-T3, Beijing Zhongjiao Jinyuan Technology Co., Ltd) for electrode illumination. Photocurrent transients were recorded over five 20-

s on/off cycles (initial voltage: 0 V, sampling: 0.1 s, sensitivity: 1×10^{-5} A V^{-1}.

The apparent quantum yield (AQY) and solar chemical conversion (SCC) measurement

The photocatalytic reaction was carried out in a photocatalytic reactor using pure deionized water (25 mL) and catalyst (20 mg). After sonication and bubbling, the bottle was irradiated by an Xe lamp at 420-800 nm (China Education Au-light Co., LTD., Beijing, China).

AQY was determined under the irradiation of a 300 W Xe lamp at a certain wavelength (λ = 400 nm, 420 nm, 450 nm, 475 nm, and 500 nm), and the light intensity was measured by a CEL-NP2000-2(10)A with a photodiode sensor. The AQY was calculated using the following equation:

$$\mathrm{AQY} = \frac{(\text{Number of produces } H_2O_2 \text{ molecules}) \times 2}{\text{Number of incident photons}} \times 100 \qquad (1)$$

$$\mathrm{AQY} = \frac{(M_{H_2O_2} \times N_A \times h \times c) \times 2}{S \times P \times T \times \lambda} \times 100 \qquad (2)$$

Where M is the yield of H_2O_2 (mol), N_A is Avogadro's constant (6.022×10^{23} mol^{-1}), h is the Planck constant (6.626×10^{-34} Js), c is the speed of light (3×10^8 ms^{-1}), *S* is the irradiation area (cm^2), *P* is the intensity of irradiation light (Wcm^{-2}), *T* is the photoreaction time (s), *λ* is the wavelength of the monochromatic light (m).

The SCC efficiency was calculated by the following equation:

$$\text{SCC efficiency (\%)} = \frac{[\Delta G \text{ for } H_2O_2 \text{ generation } (\mathrm{Jmol^{-1}})] \times [H_2O_2\ formed\ (mol)]}{[\text{Total input power (W)}] \times [\text{Reaction times (s)}]} \times 100 \quad (3)$$

where ΔG= 117 kJ mol^{-1}, the irradiated area is 12.57 cm^2, the total input energy was therefore 1.257 W.

Rotating disk electrode (RDE) measurements: The RDE performance was studied on a Gamry Reference 600 workstation (USA) configured with a standard three-electrode cell. To prepare the catalyst ink, 10 mg of catalyst was ultrasonically dispersed in a 0.9 mL ethanol/0.1 mL Nafion mixture to form a homogeneous ink. 10 μL of the prepared slurry was dropped on the disk electrode and dried at room temperature (25 ± 2 °C). A glassy carbon rotating disk electrode with a diameter of 5 mm served as the substrate for the working electrode, Ag/AgCl as the reference electrode, and Pt wire as the counter electrode. The linear sweep voltammetry (LSV) electrochemical tests were conducted at room temperature in an O_2-saturated phosphate buffer solution (PBS, 0.1 M, pH=7) with a scan rate of 10 mV s^{-1} and different rotation speeds (100-900 rpm). The average number of electrons transferred (n) during RDE measurements was calculated based on the Koutecky-Levich equation:

$$\frac{1}{J}=\frac{1}{J_L}+\frac{1}{J_K}=\frac{1}{B\omega^{\frac{1}{2}}}+\frac{1}{J_K} \quad (4)$$

$$B=0.62n\mathrm{F}\mathrm{C}_0\mathrm{D}_0^{\frac{2}{3}}\mathrm{v}^{-\frac{1}{6}} \quad (5)$$

Where J is the measured current density, J_K and J_L are the kinetic and diffusion limiting current densities, ω is the angular velocity of the disk in rpm ($\omega=2\pi N$, N is the rotation speed in rad s^{-1}), n is the transferred electron number, F is the Faraday constant (96485 C mol^{-1}), C_0 is the bulk concentration of O_2 (1.2×10^{-6} mol cm^{-3}), D_0 is the diffusion coefficient of O_2 in 0.1 M PBS (2.7×10^{-5} cm^2 s^{-1}), and ν is the kinematic viscosity of the electrolyte (0.01 cm^2 s^{-1}).

Electron paramagnetic resonance (EPR) Measurements: 5,5-dimethyl-1-pyrroline N-oxide (DMPO) was used as a spin-trapping reagent to detect hydroxyl radicals (•OH) or superoxide radicals ($\bullet O_2^-$). In particular, the catalysts (2 mg) were dispersed in water or a MeOH/water mixture (9/1 v/v, 500 μL) containing DMPO (0.1 mmol) in a Pyrex glass tube sealed with a rubber septum cap. A Xe lamp (λ > 420 nm) was used as the light source. The dispersion was purged with Ar or O_2 gas for 5 min before light irradiation. 2,2,6,6-Tetramethyl-4-piperidone (TEMP) served as the spin-trapping agent for the detection of $^1O_2$. The catalysts (2 mg) were dispersed into water (500 μL) containing TEMP (0.1 mmol) with a Pyrex glass tube, which was sealed with a rubber septum cap. A Xe lamp (λ > 420 nm) was used as the light source. The dispersion was purged with O_2 gas for 5 min before light irradiation.

In the oxidative coupling of benzylamines, the EPR test is similar, replacing water with an acetonitrile/ benzylamines mixture (9/1 v/v, 500 μL).

Isotope labeling experiments: The $^{18}O_2$ isotope was measured on a GC-MS (Clarus 690, PerkinElmer). The detailed processes of isotope labeling experiments were according to the literature method[3-5].

Photocatalysts (5 mg) and pure water ($H_2{}^{16}O$, 2 mL) were added to a 10 mL glass reactor. The reaction mixture was deoxygenated 3 times and backfilled with highly pure N_2 (99.999%). 5 mL $^{18}O_2$ was injected into the reactor via syringe, and the reaction was performed for 4 h under the irradiation from a 300 W xenon lamp at ambient temperature. In a separate 5 mL reactor, 100 mg of MnO_2 was introduced, and Ar was purged to eliminate O_2. The light-induced H_2O_2 solution was then added to the reactor to generate O_2, which was subsequently quantified by mass spectrometry.

$$^{18}O_2 + 2H^+ + 2e^- = H_2{}^{18}O_2$$

$$2H_2{}^{16}O + 2h^+ = H_2{}^{16}O_2 + 2H^+$$

$$2H_2O_2 \xrightarrow{MnO_2} 2H_2O + O_2$$

In-situ diffuse reflectance infrared Fourier transform spectroscopy (DRIFTS): In-situ DRIFTS measurements were performed based on the literature method[4], using a Nicolet iS-50 Fourier-transform spectrometer equipped with a Harrick diffuse reflectance accessory. Each spectrum was recorded by averaging 256 scans at a spectral resolution of 4 cm^{-1}. The samples were held in a custom-made IR reaction chamber, which was specifically designed to examine highly scattering powder samples in the diffuse reflection mode. The chamber was sealed with two ZnSe windows.

Theoretical calculations: All theoretical calculations were performed using the DFT method, as implemented in the Vienna ab initio simulation package (VASP)[6-8]. The core electrons were described using the spin-polarized projector augmented wave (PAW) method[9], and the electron exchange and correlation energy was treated within the generalized gradient approximation in the Perdew-Burke-Ernzerhof functional (GGA-PBE)[10]. The valence states of all atoms were expanded in a plane-wave basis set with a cutoff energy of 450 eV. The convergence criteria for the electronic self-consistent iteration and force were set to 10^{-5} eV and 0.02 eV/Å with a Gamma-centered 1 × 1 × 1 K-points. Denser 3 × 3 × 1 K-points were used for electronic structure analysis. In addition, the Van der Waals interactions were included during all calculations using DFT-D3[11-12]. The slab was modeled by one layer. During the simulation, the slabs were kept fully relaxed and slab model was constructed with a vacuum layer of 15 Å in the z direction to avoid the interaction between neighboring images. The charge density differences were evaluated using the formula $\Delta\rho = \rho_{A+B} - \rho_A - \rho_B$, where ρ_X is the electron density of X. Atomic charges were computed using the atom-in-molecule (AIM) scheme proposed by Bader[13-14].

To explore the catalytic effect, the change of Gibbs free energy (ΔG) was calculated, which is defined as:

$$\Delta G = \Delta E + \Delta ZPE + \Delta H_{0\rightarrow 298K} - T\Delta S \quad (6)$$

Where ΔE is the energy change obtained from DFT calculations; ΔZPE, ΔH, and ΔS denote the difference in zero-point energy, enthalpy, and entropy due to the reaction, respectively. The enthalpy and entropy of the ideal gas molecule were taken from the standard thermodynamic tables, and some of the calculation results were analyzed by the VASPKIT package[15].

Supporting Figures

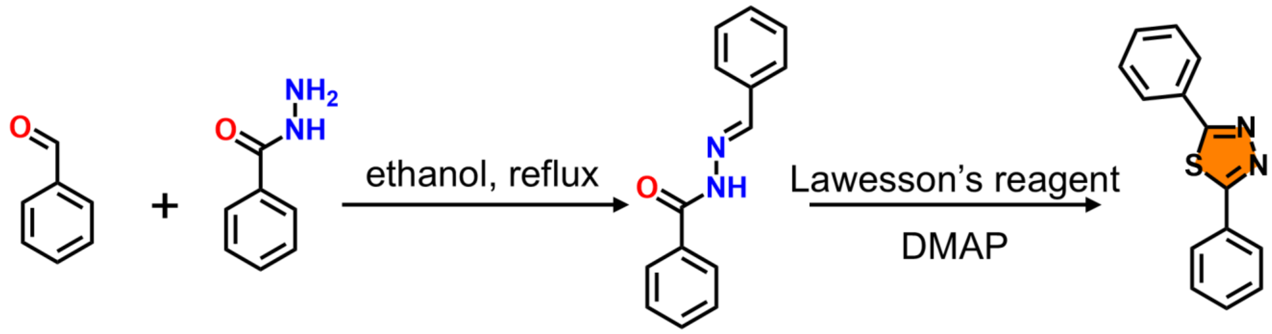

Fig. S1. Synthesis of the model compound through post-synthetic modification.

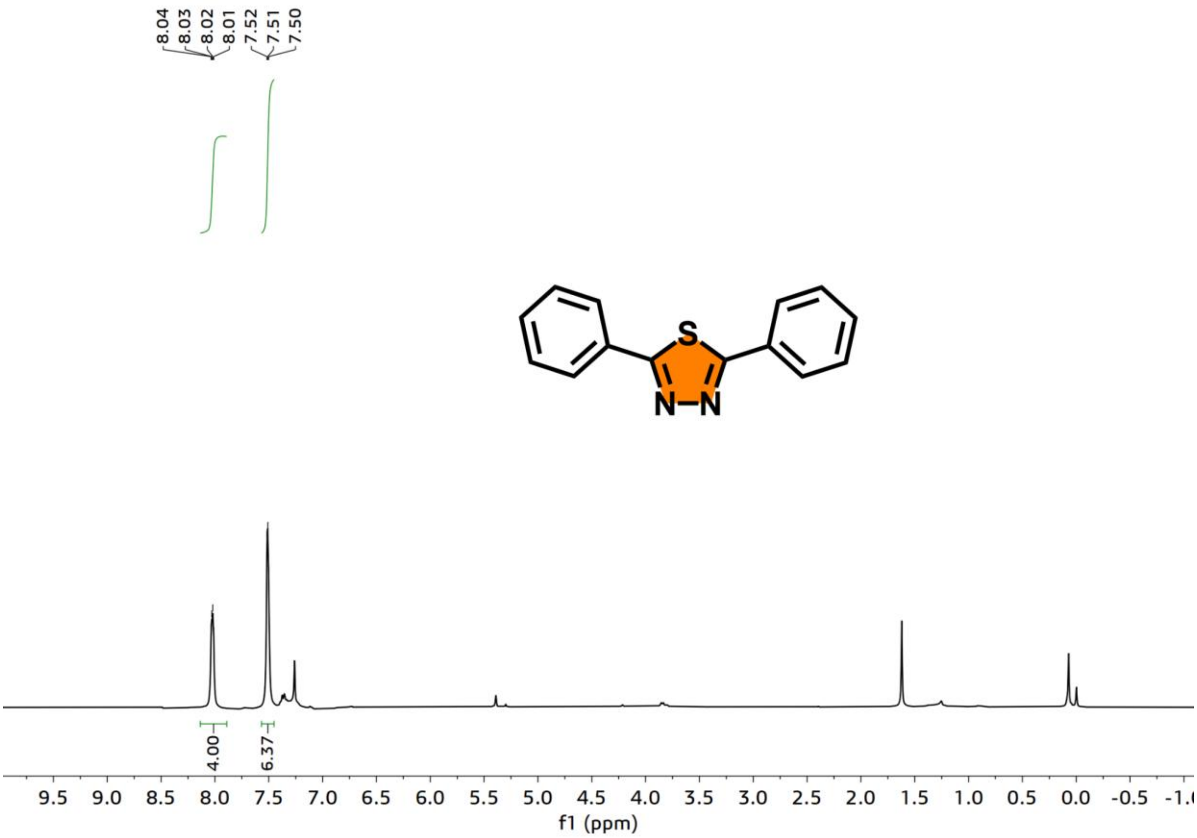

Fig. S2. ^{1}H-NMR spectra of the model compound.

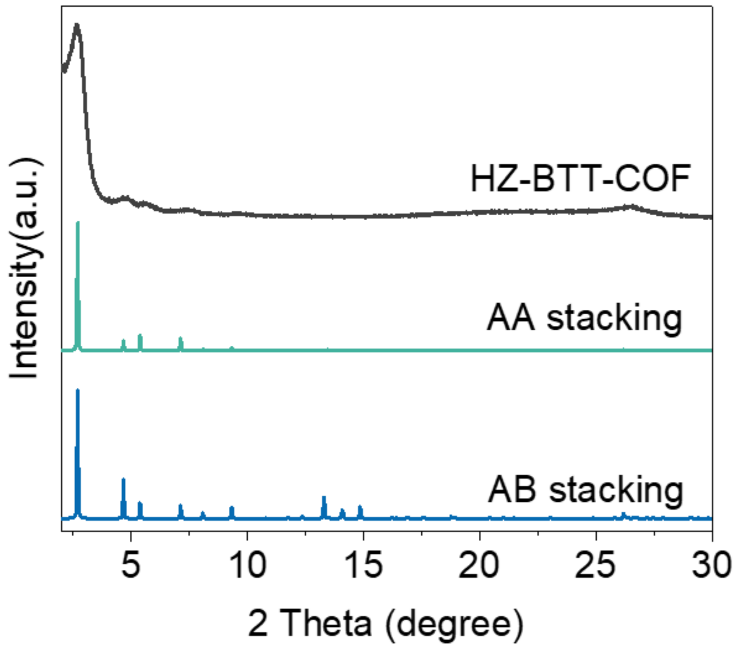

Fig. S3. Simulated X-ray diffraction patterns for generated **hcb** hexagonal layered structures adopting fully eclipsed (green) and staggered (blue) stacking arrangements compared to the experimental pattern of HZ-BTT-COF (black), a.u. indicates the arbitrary units.

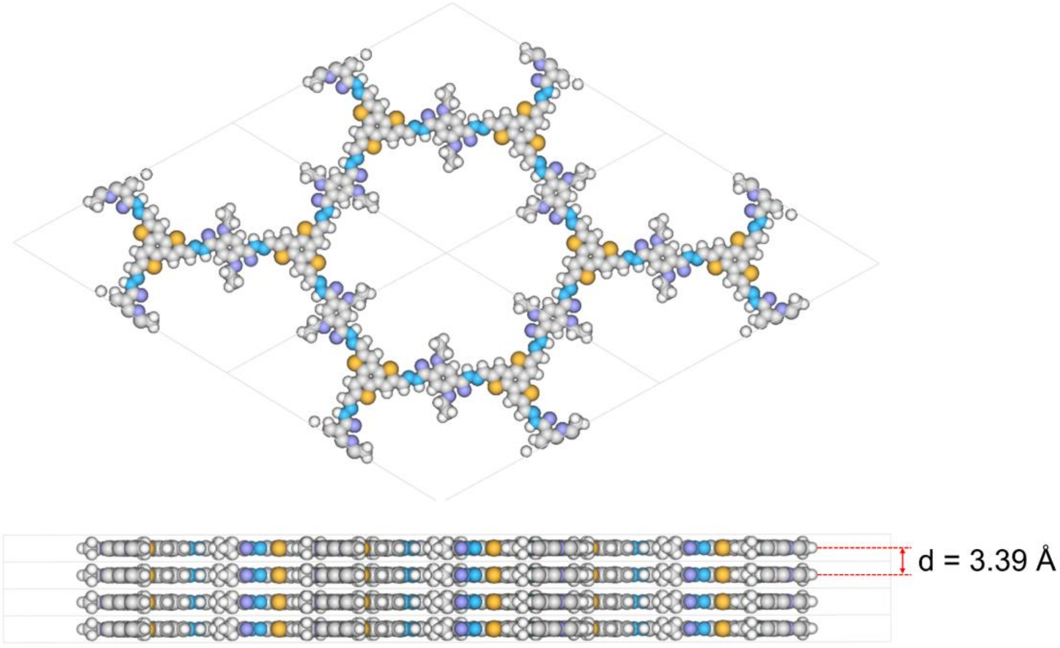

Fig. S4. Simulated **hcb** 2D hexagonal layered model with eclipsed (AA) stacking arrangement of HZ-BTT-COF.

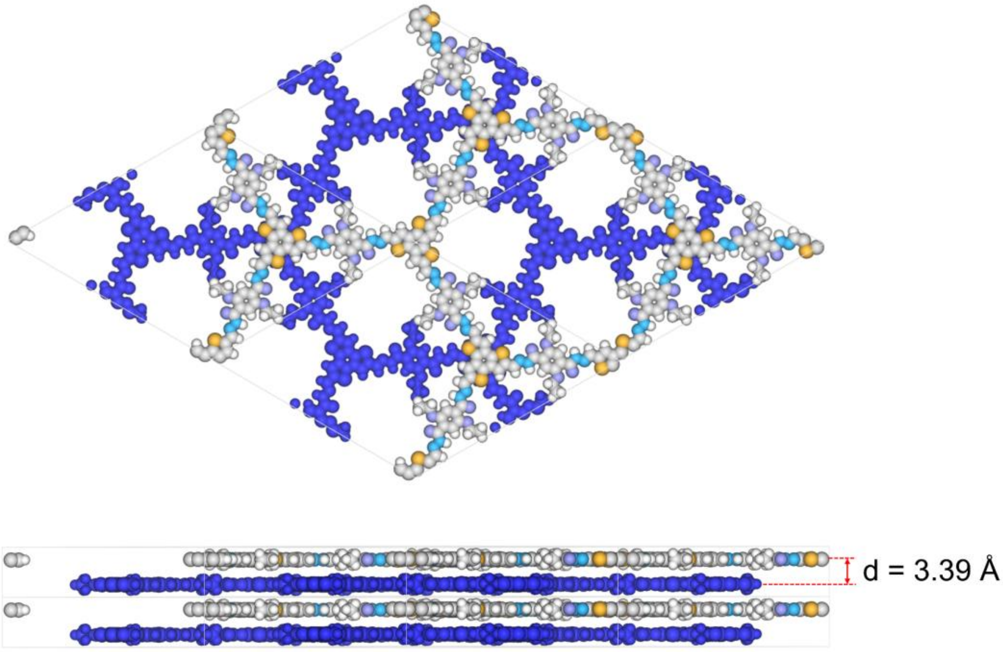

Fig. S5. Simulated **hcb** 2D hexagonal layered model with staggered (AB) stacking arrangement of HZ-BTT-COF.

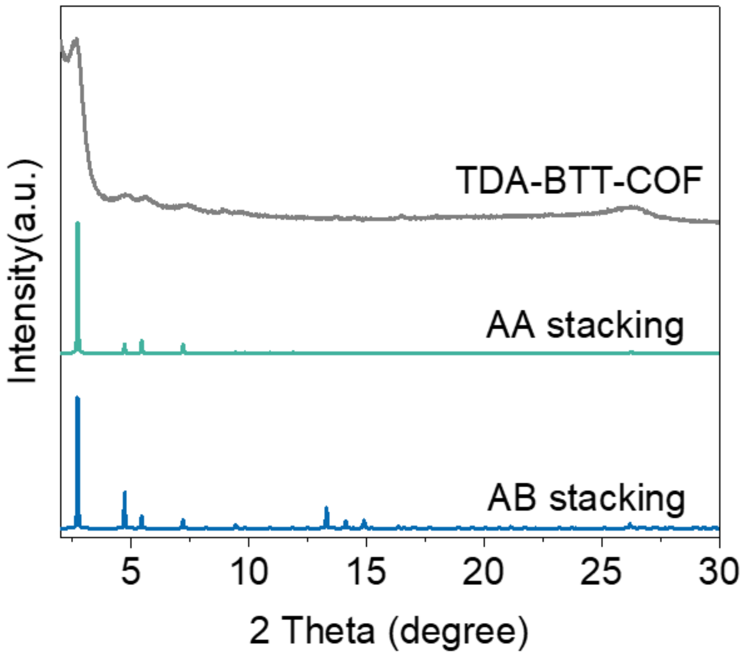

Fig. S6. Simulated X-ray diffraction patterns for generated **hcb** hexagonal layered structures adopting fully eclipsed (green) and staggered (blue) stacking arrangements compared to the experimental pattern of TDA-BTT-COF (black), a.u. indicates the arbitrary units.

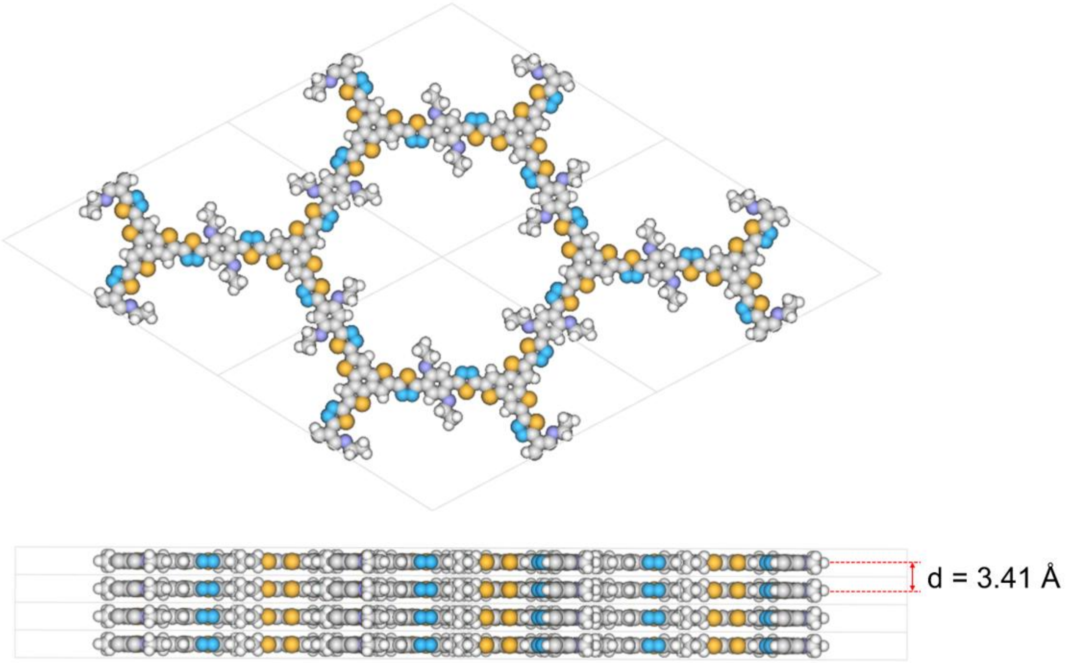

Fig. S7. Simulated **hcb** 2D hexagonal layered model with eclipsed (AA) stacking arrangement of TDA-BTT-COF.

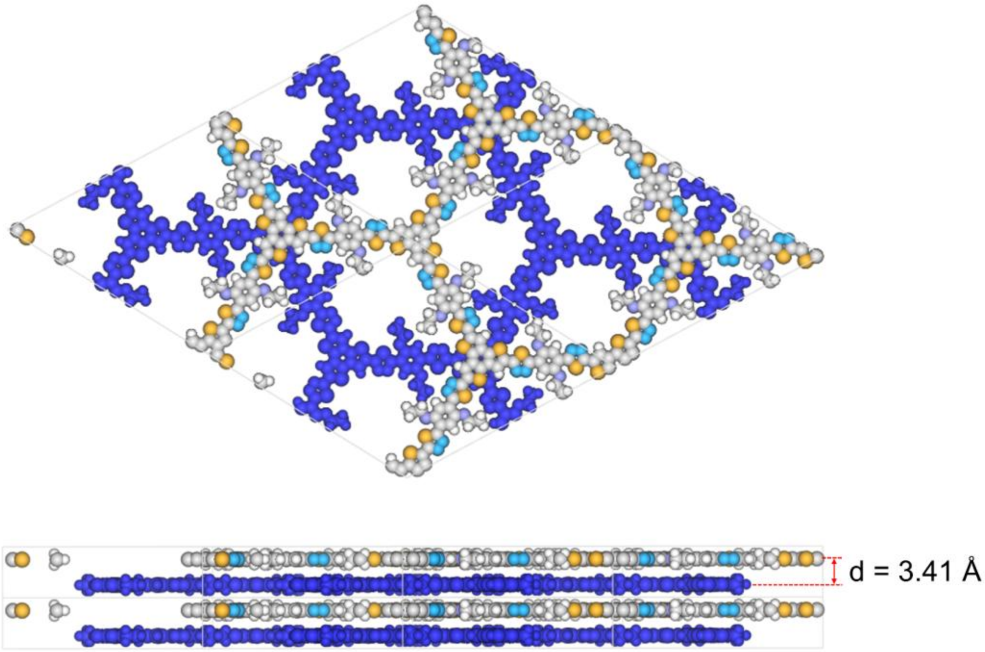

Fig. S8. Simulated **hcb** 2D hexagonal layered model with staggered (AB) stacking arrangement of TDA-BTT-COF.

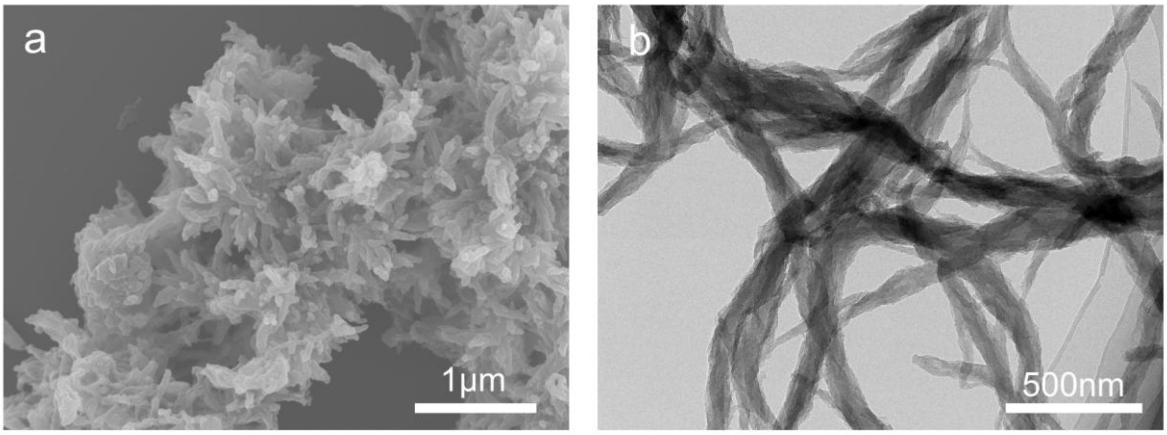

Fig. S9. The morphology characterization of HZ-BTT-COF. a) SEM image and b) TEM image.

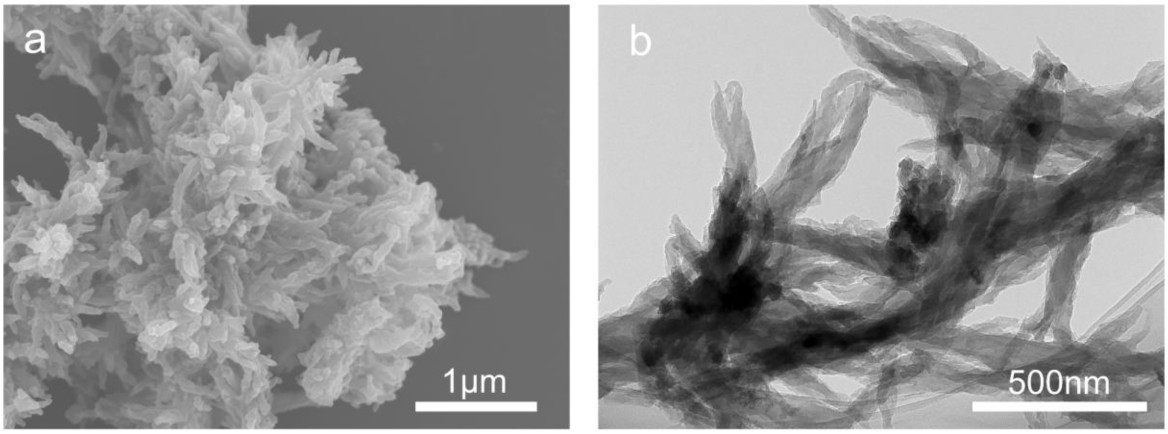

Fig. S10. The morphology characterization of TDA-BTT-COF. a) SEM image and b)TEM image.

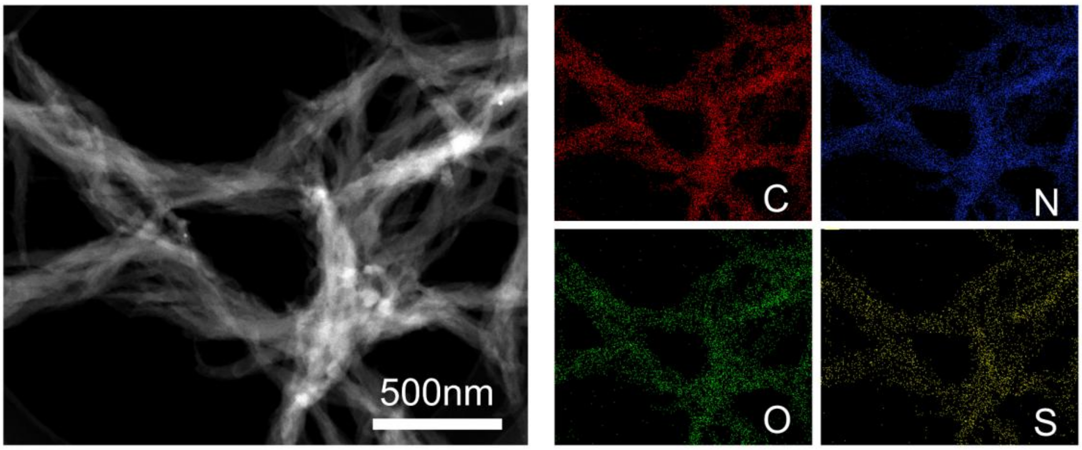

Fig. S11. High-angle annular dark-field (HAADF)-STEM image and the corresponding elemental mapping images of HZ-BTT-COF.

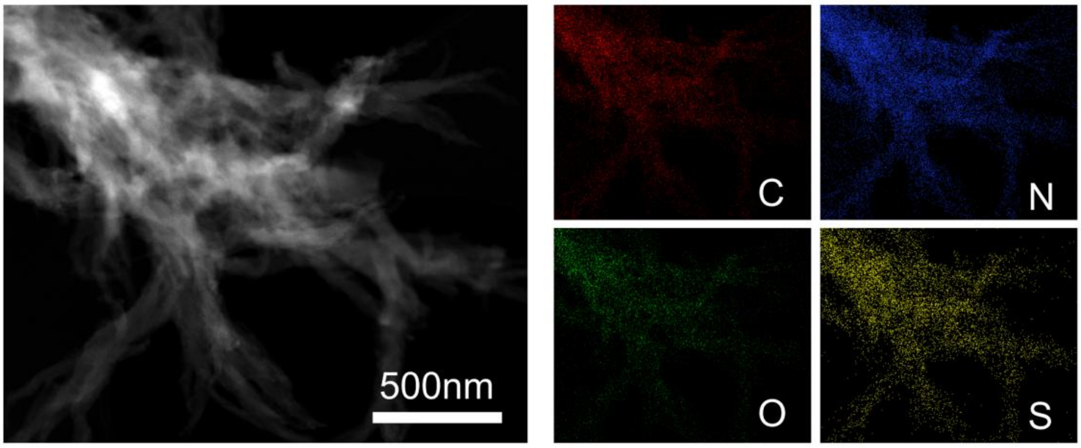

Fig. S12. HAADF-STEM image and the corresponding elemental mapping images of TDA-BTT-COF.

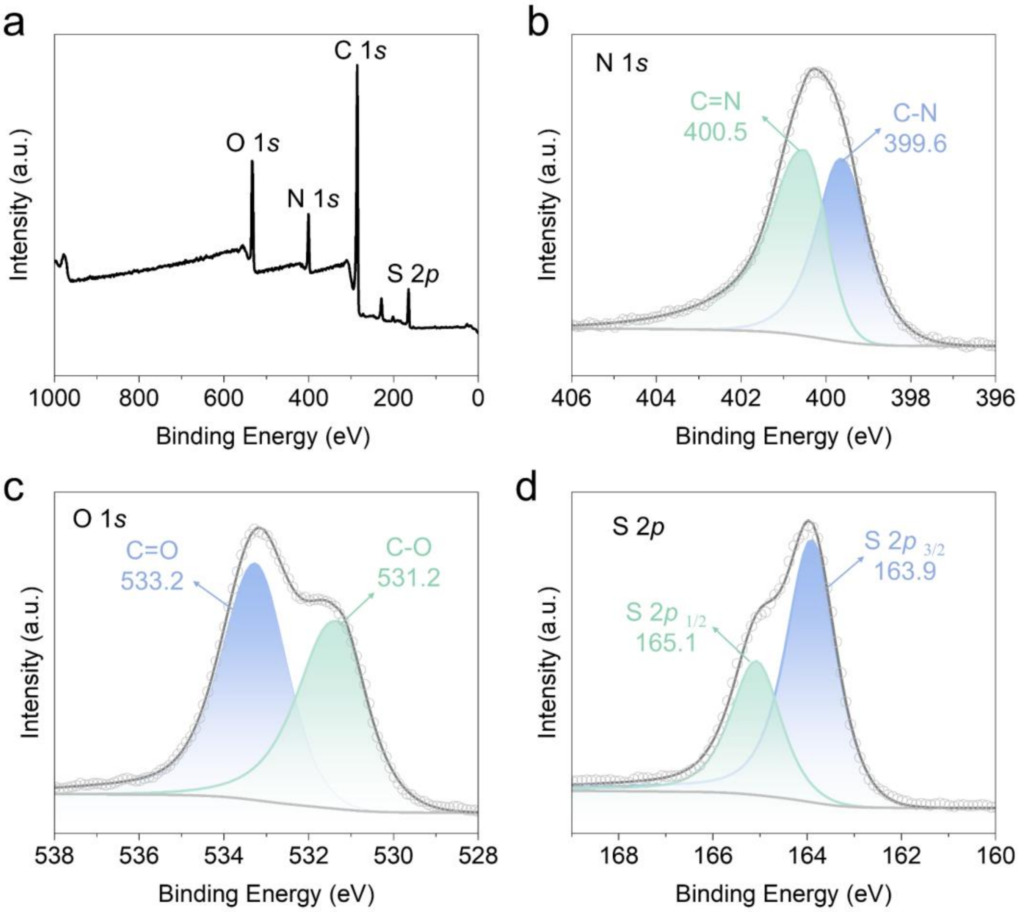

Fig. S13. XPS spectra of HZ-BTT-COF. a) XPS survey spectra, b) N 1*s*, c) O 1*s*, and d) S 2*p*, a.u. indicates the arbitrary units.

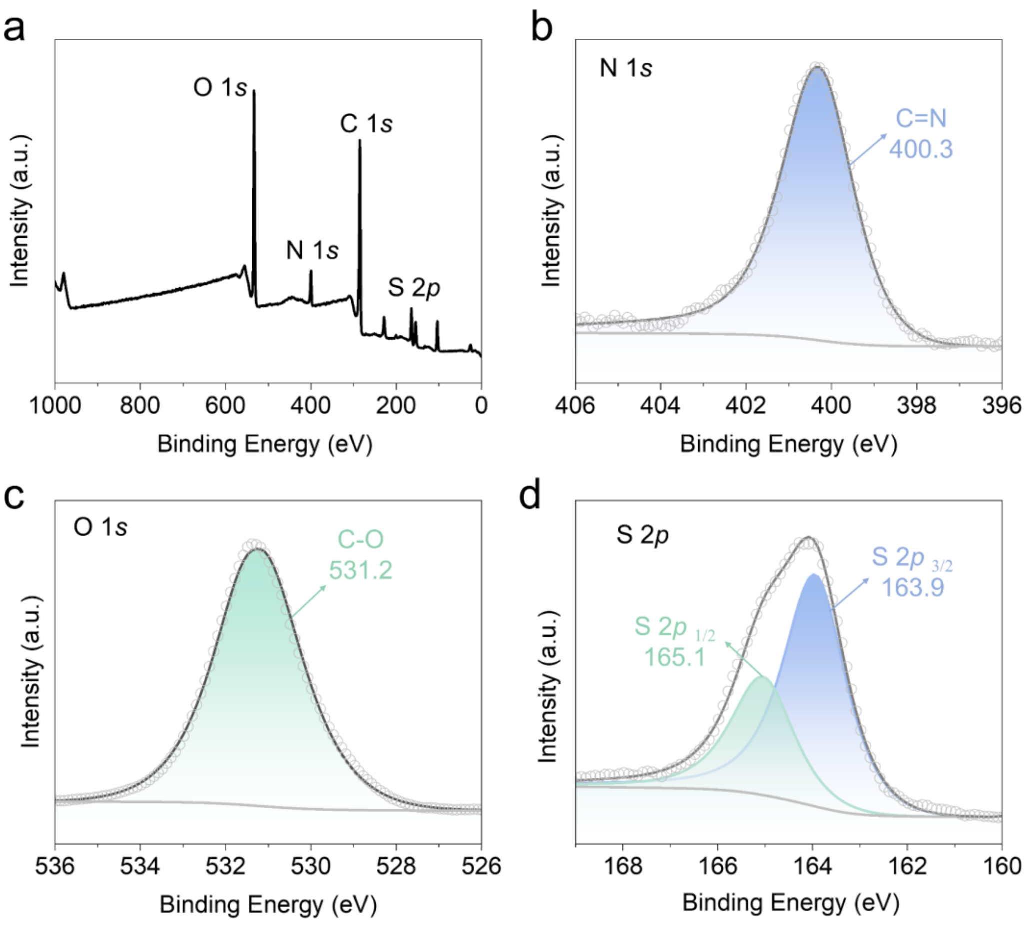

Fig. S14. XPS spectra of TDA-BTT--COF. a) XPS survey spectra, b) N 1*s*, c) O 1*s*, and d) S 2*p*. The N 1*s* XPS spectrum of HZ-BTT-COF exhibits two distinct deconvoluted peaks at 399.6 eV and 400.5 eV, corresponding to imine (-C=N-) and amide (-N-C=O) nitrogen species, respectively. In contrast, TDA-BTT-COF exhibits a single nitrogen environment, characterized by a peak at 400.3 eV, which is attributable to nitrogen atoms incorporated into thiadiazole. Similarly, the O 1*s* spectrum of HZ-BTT-COF reveals two resolved components at 533.2 eV (C=O) and 531.2 eV (C-O), whereas TDA-BTT-COF displays a singular oxygen species at 531.2 eV associated with ethoxy groups ($-OCH_2CH_3$). These comparative spectroscopic analyses collectively confirm the successful structural transformation of HZ-BTT-COF into TDA-BTT-COF, a.u. indicates the arbitrary units.

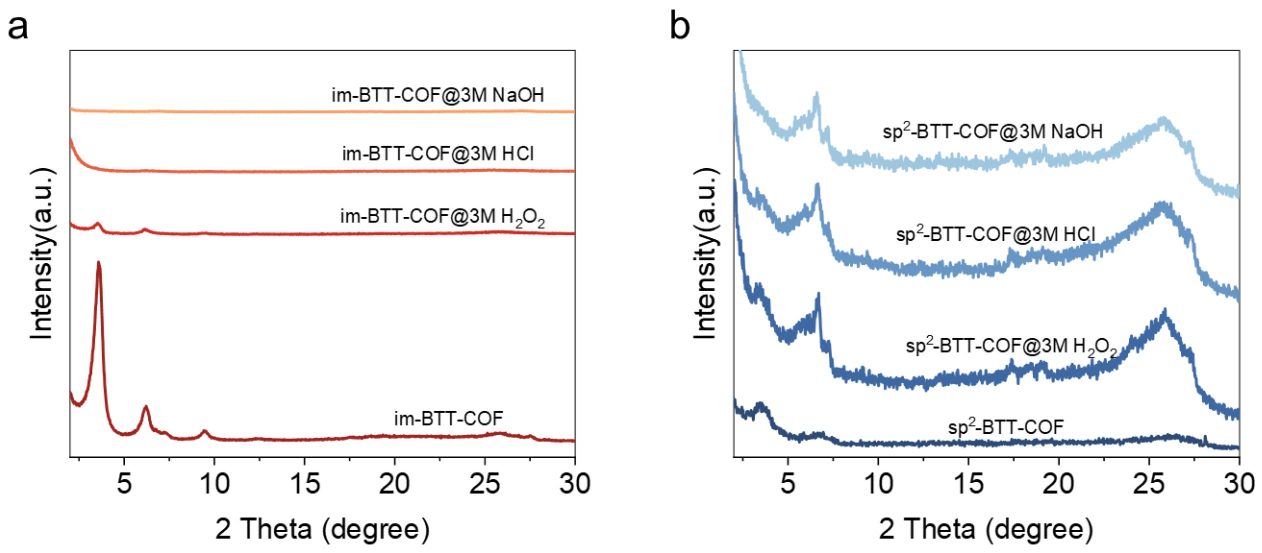

Fig. S15. Chemical stability test of im-BTT-COF and sp^2-BTT-COF after 12 h of treatment with 3 M HCl, 3 M NaOH, and 3 M H_2O_2.

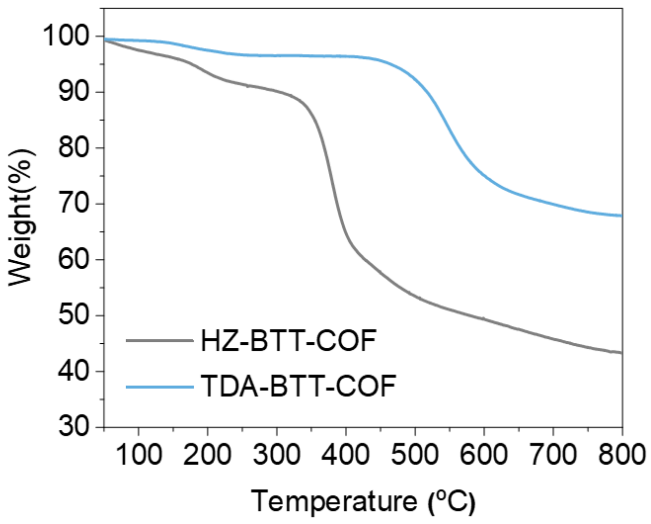

Fig. S16. Thermogravimetric analysis (TGA) of HZ-BTT-COF and TDA-BTT-COF under N_2 atmosphere.

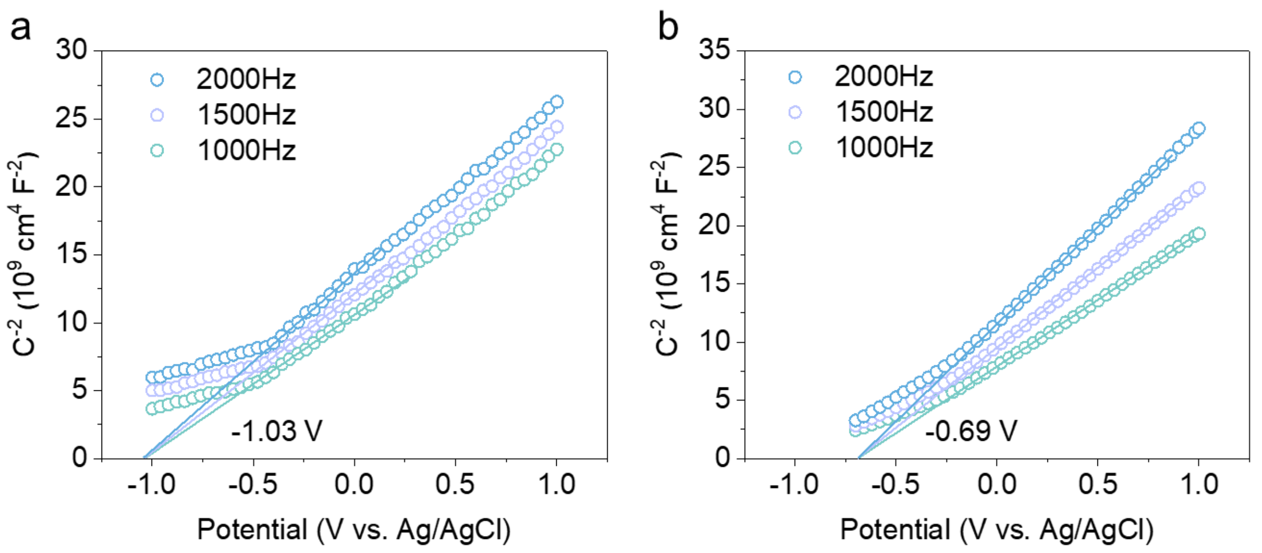

Fig. S17. Mott-Schottky plots of a) HZ-BTT-COF and b) TDA-BTT-COF. E_{CB} (V vs. NHE) was calculated according to the formula: E(NHE) = E(Ag/AgCl) + 0.197.

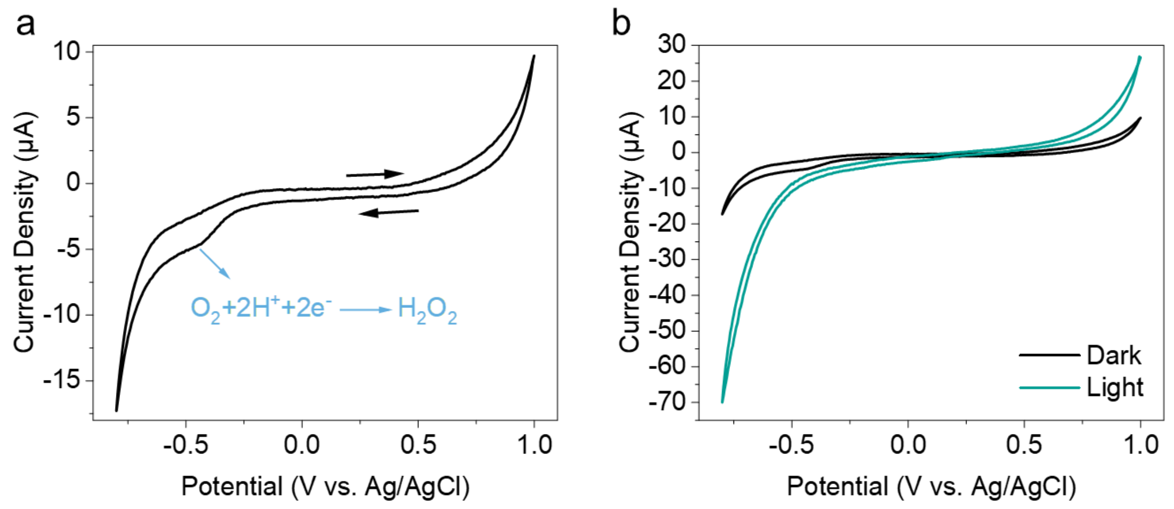

Fig. S18. The CV curves of the TDA-BTT-COF photoelectrode under a) dark (black) and b) illuminated condition (green).

CV measurement: The CV curves of the TDA-BTT-COF electrode were recorded using a three-electrode setup. 0.5 M Na_2SO_4 was used as the electrolyte, and the scan was performed with a scanning rate of 50 mV/s. The oxygen reduction reaction (ORR) for H_2O_2 production is clearly evident in the CV curves, and we found that the current increased under visible-light illumination, leading to blurring of the ORR characteristic peak.

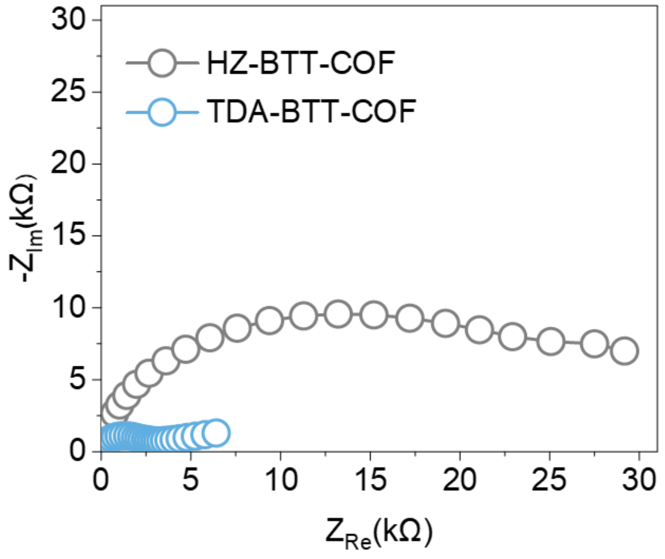

Fig. S19. Nyquist plots of HZ-BTT-COF and TDA-BTT-COF.

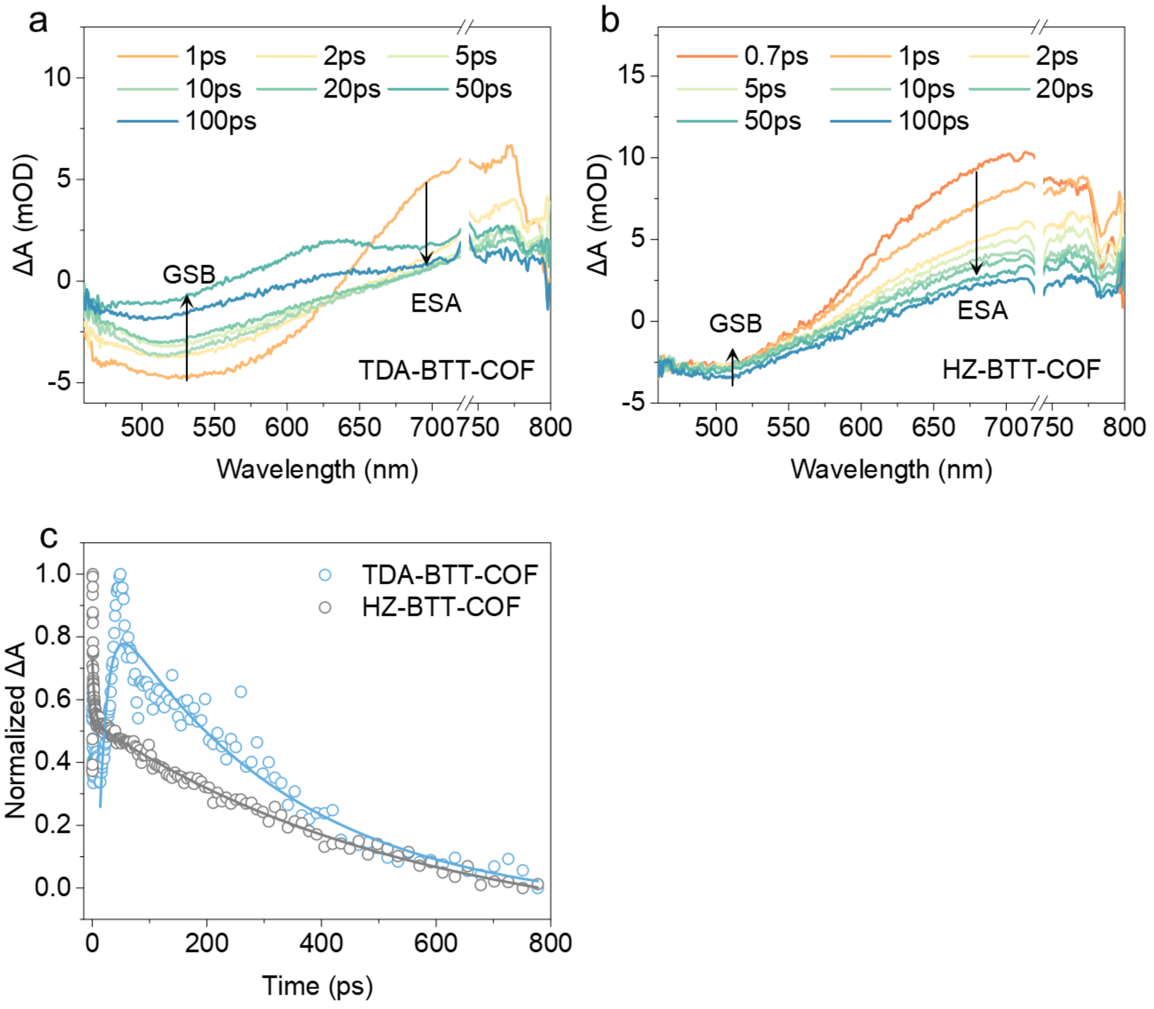

Fig. S20. The femtosecond-transient absorption (fs-TA) signals of a) TDA-BTT-COF and b) HZ-BTT-COF

on the fs-ns timescales (370 nm excited). c) fs-TA decay kinetics profiles of TDA-BTT-COF and HZ-BTT-COF monitored at the wavelength of 630 nm.

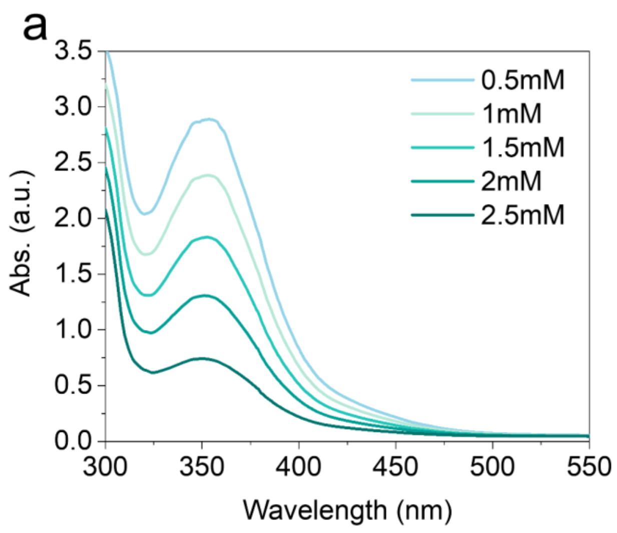

Fig. S21. The standard curve of H_2O_2 concentration-absorbance by Iodometry. a) A known concentration of H_2O_2 solution was added to the KI solution, and the change of absorption intensity at 350 nm was measured by a UV-vis spectrometer. b) The H_2O_2 concentration - absorbance standard curve, a.u. indicates the arbitrary units.

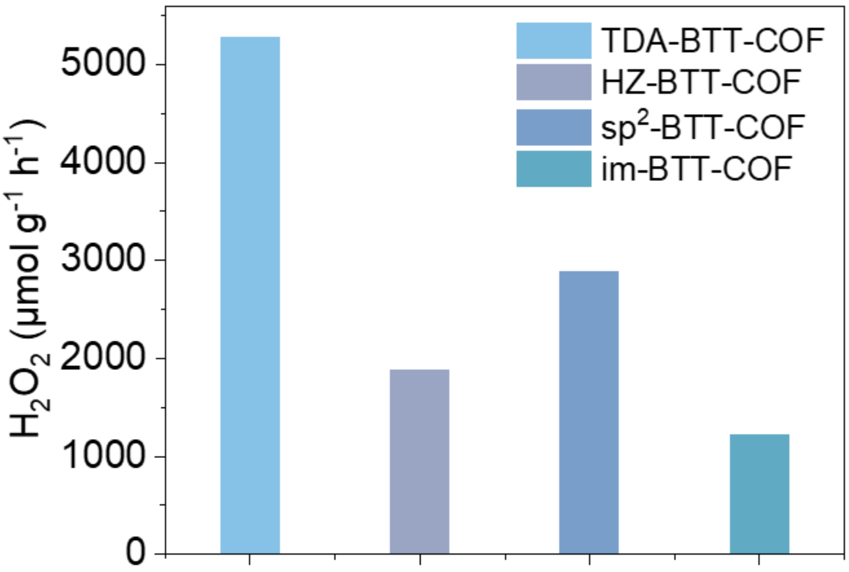

Fig. S22. Photocatalytic H_2O_2 production of different COFs (5 mg of COFs in 25 mL of deionized water at 25 °C and 300 W Xe lamp, λ > 420 nm).

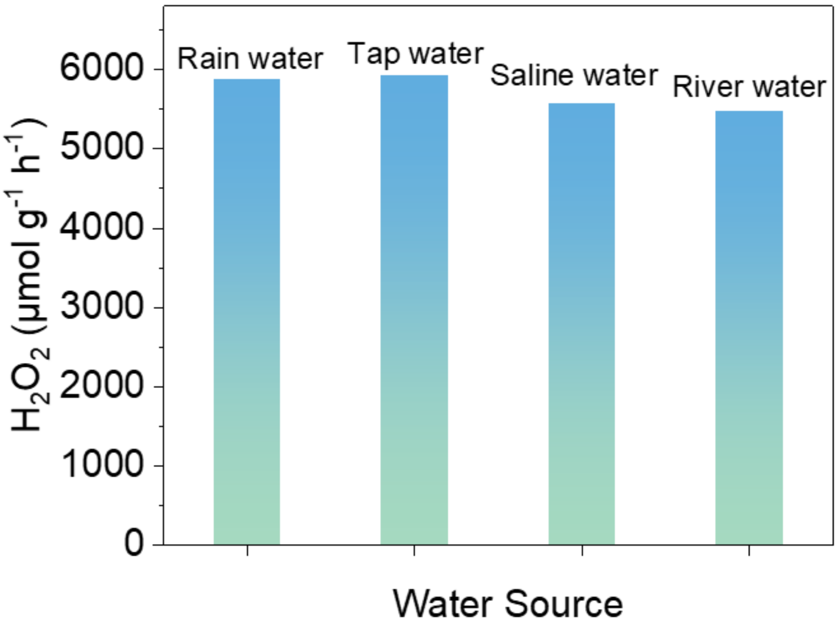

Fig. S23. Photocatalytic H_2O_2 production in rainwater, tap water, saline water, and river water (5 mg of COFs in 25 mL of various water sources at 25 °C and 300 W Xe lamp, λ > 420 nm).

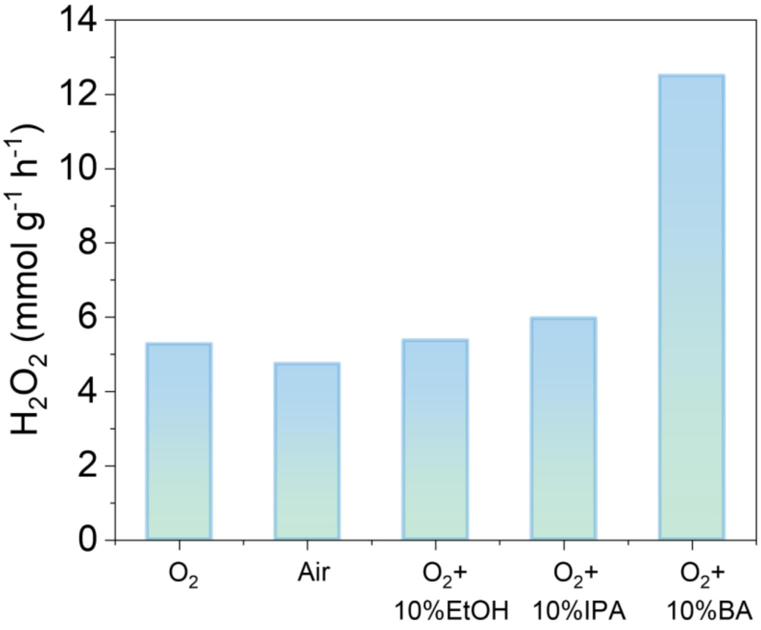

Fig. S24. Photocatalytic H_2O_2 production under different gas atmospheres and sacrificial agents.

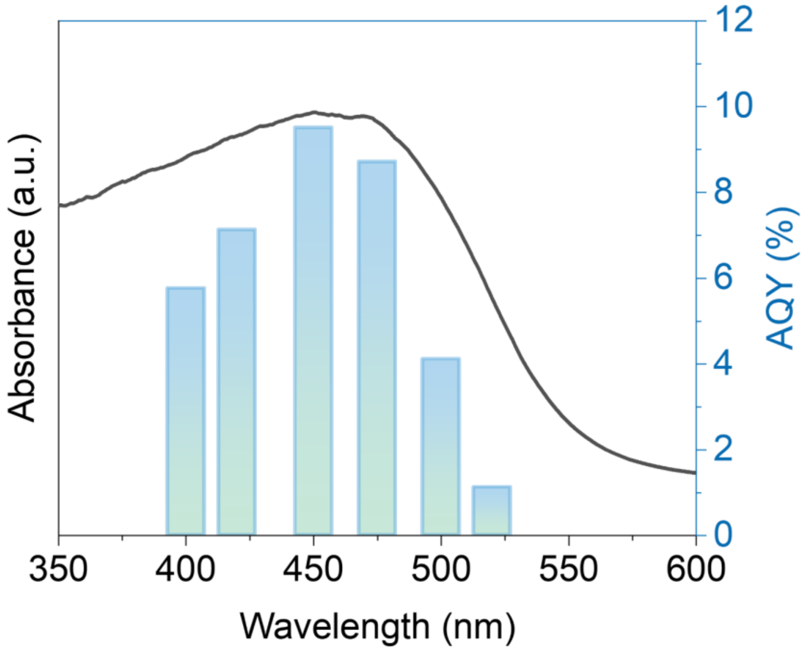

Fig. S25. Wavelength-dependent AQY measurement for TDA-BTT-COF, a.u. indicates the arbitrary units.

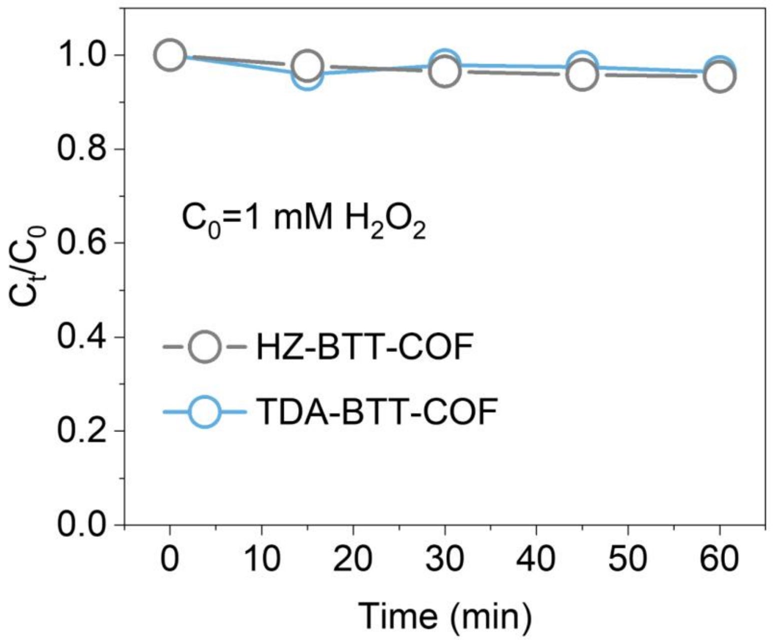

Fig. S26. Photocatalytic decomposition of H_2O_2 (C_0 = 1 mM) in deionized water under an Ar atmosphere over HZ-BTT-COF and TDA-BTT-COF.

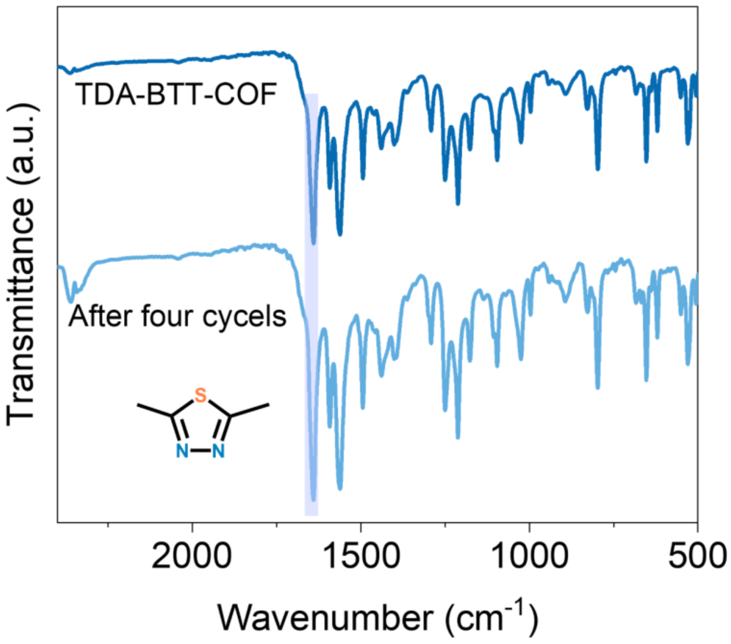

Fig. S27. FTIR spectra for TDA-BTT-COF before and after four runs of photocatalytic H_2O_2 production in deionized water (COFs were regenerated by washing with acetone and MeOH), a.u. indicates the arbitrary units.

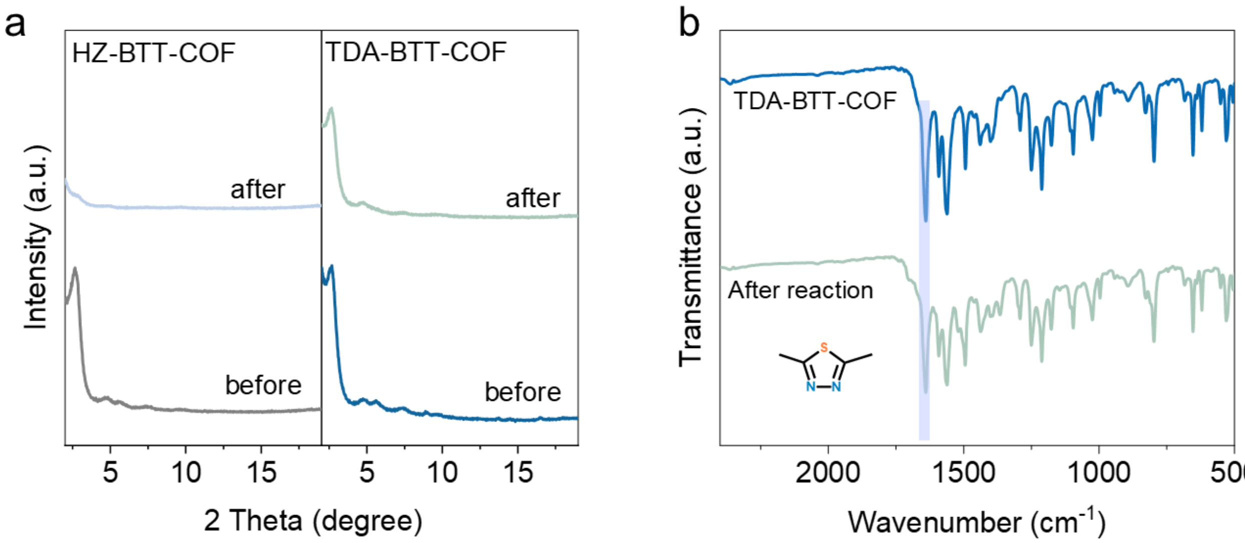

Fig. S28. a) PXRD patterns and b) FTIR spectra for TDA-BTT-COF before and after photocatalytic H_2O_2 production in seawater (COFs were regenerated by washing with acetone and MeOH), a.u. indicates the arbitrary units.

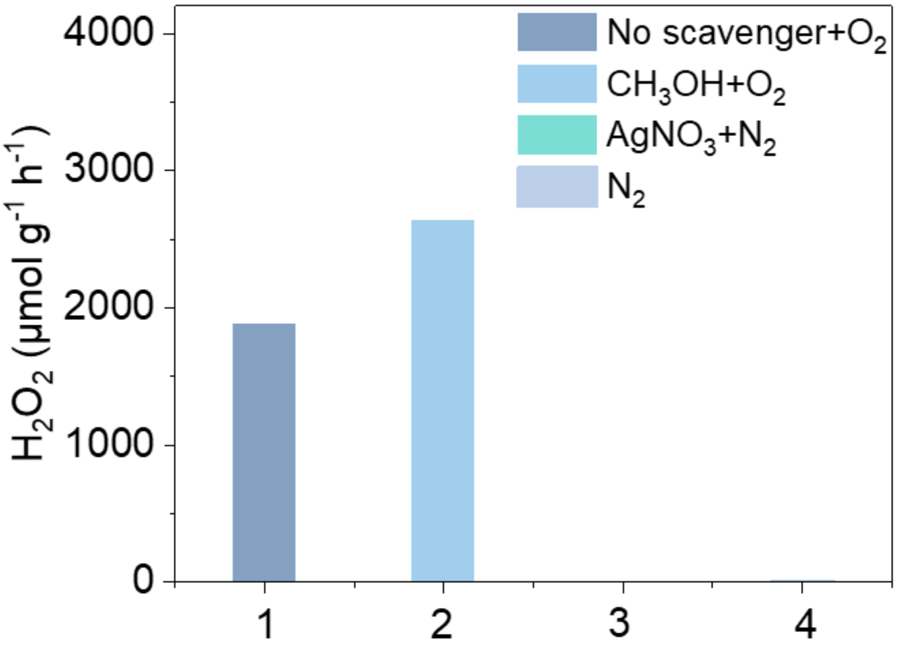

Fig. S29. Amount of H_2O_2 generated on HZ-BTT-COF in CH_3OH (10% v/v, as the hole trapping agent), H_2O_2 produced in Ar and $AgNO_3$ (0.01 M). Conditions: water (25 mL), catalyst (5 mg), 300 W Xe lamp, λ > 420 nm.

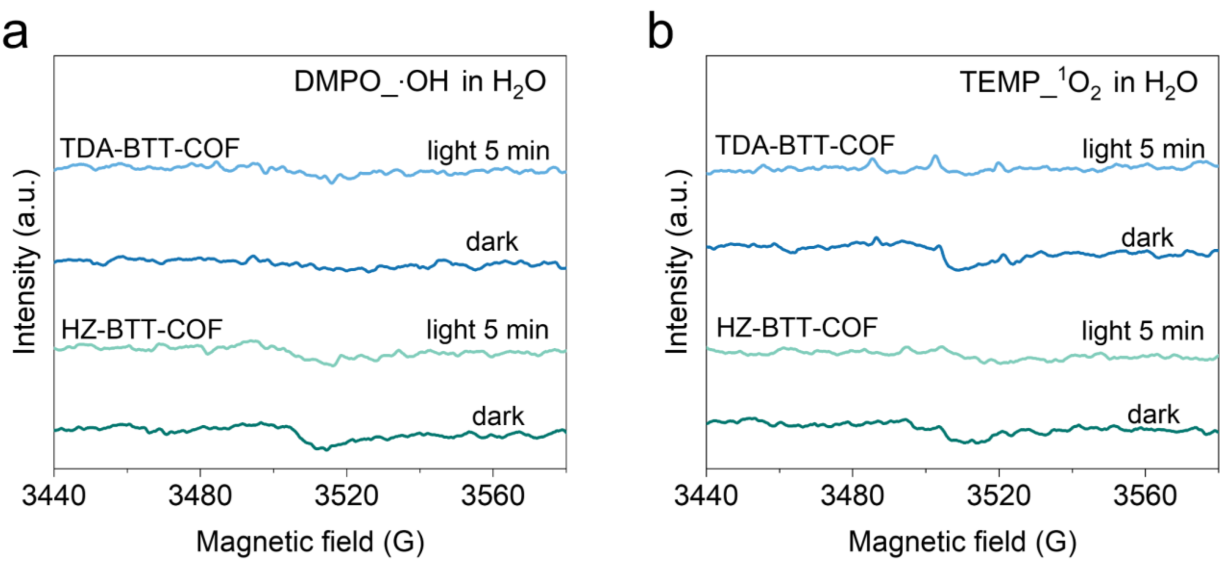

Fig. S30. EPR signals of the reaction solution under dark and light irradiation in the presence of a) DMPO and b) TEMP as the spin-trapping reagents. (300 W Xenon lamp, 5 min irradiation; O_2 saturated; H_2O), a.u. indicates the arbitrary units.

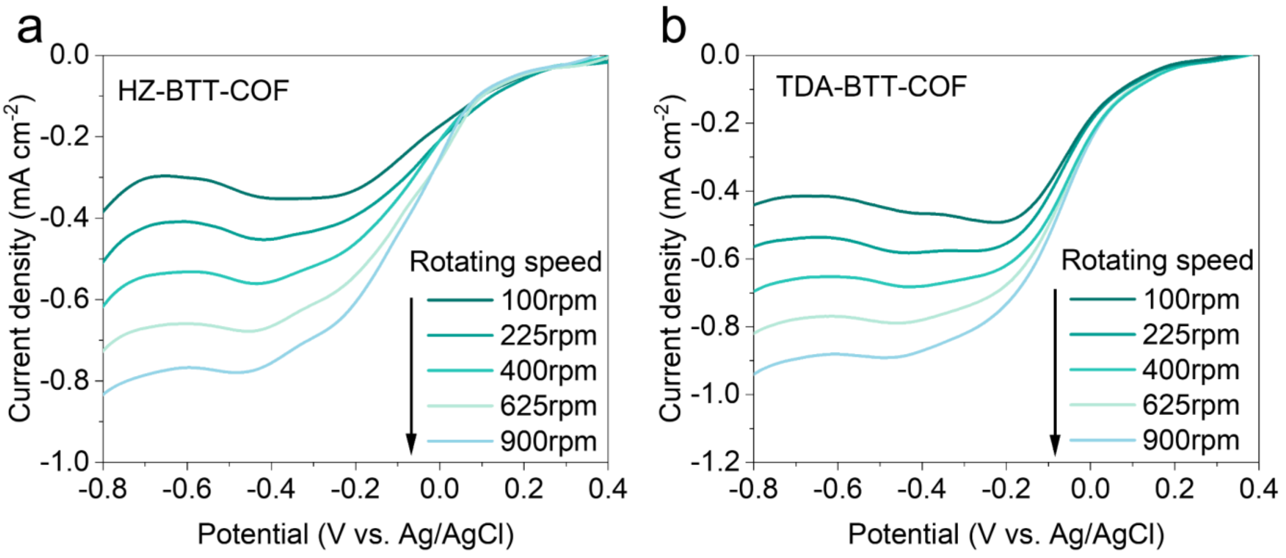

Fig. S31. LSV curves of a) HZ-BTT-COF and b) TDA-BTT-COF measured on RDE at different rotating speeds.

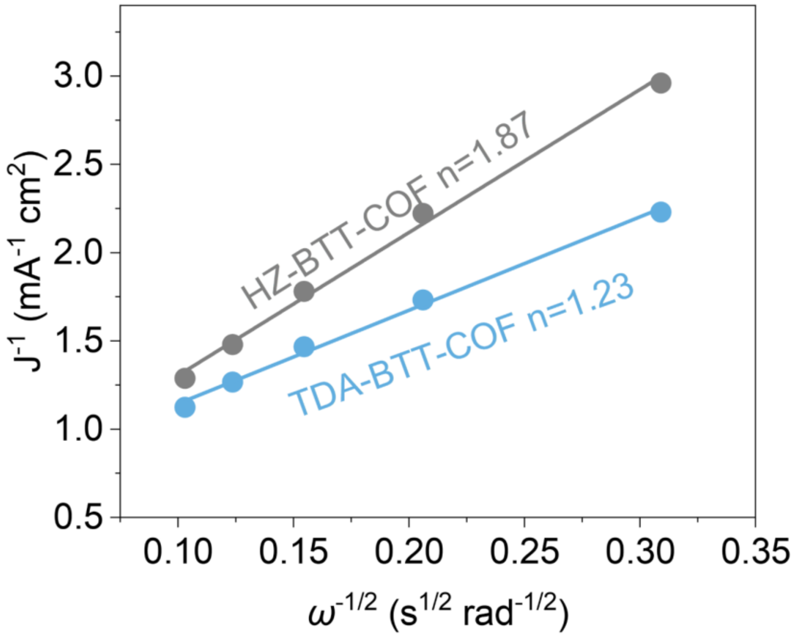

Fig. S32. Koutecky–Levich plots for TDA-BTT-COF obtained by RDE tests versus Ag/AgCl (The data were fitted from -0.45 V).

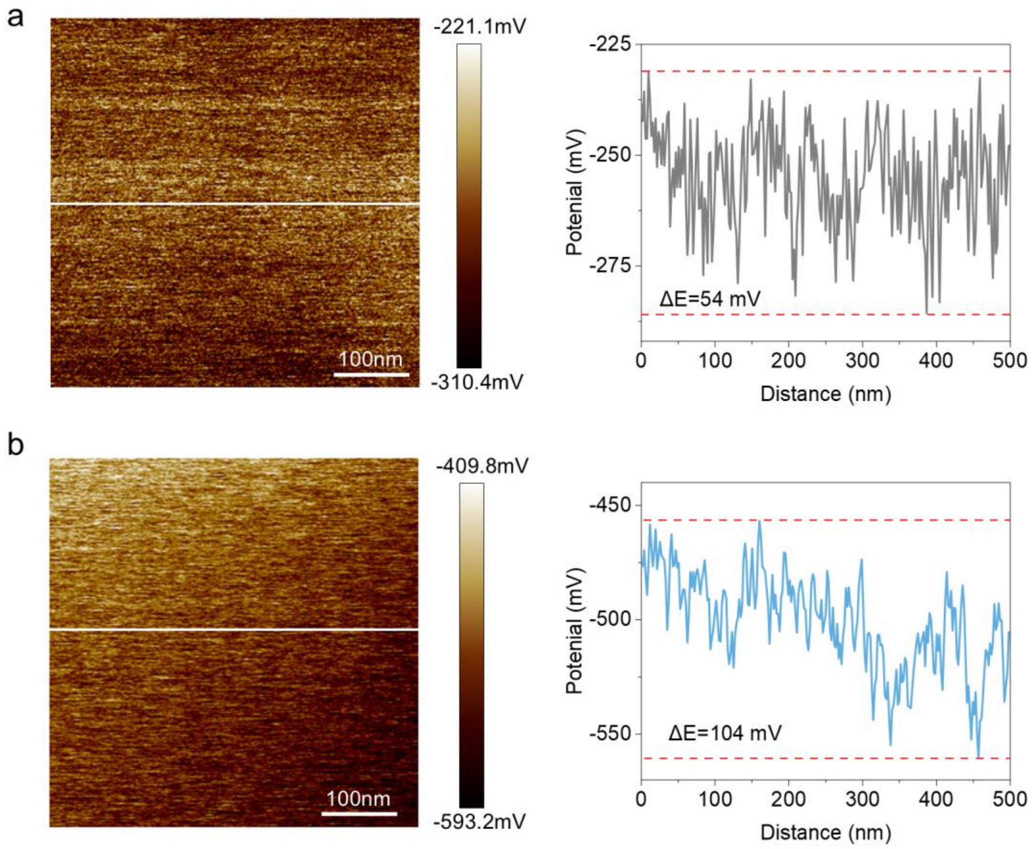

Fig. S33. Surface potential of a) HZ-BTT-COF and b) TDA-BTT-COF, as determined by Kelvin probe force microscopy.

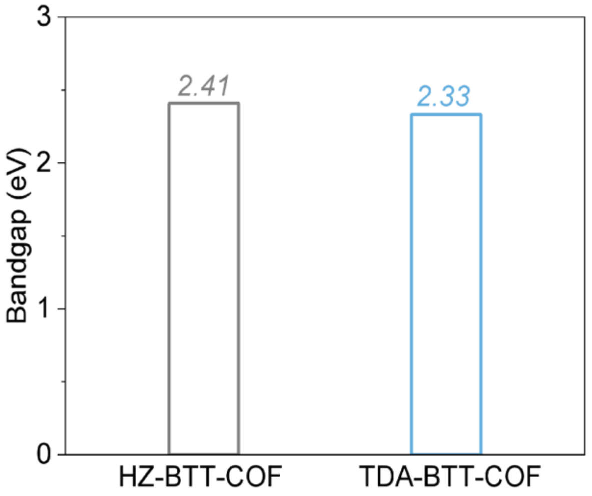

Fig. S34. Band gaps of HZ-BTT-COF and TDA-BTT-COF by the Heyd-Scuseria-Ernzerhof (HSE06) hybrid functional.

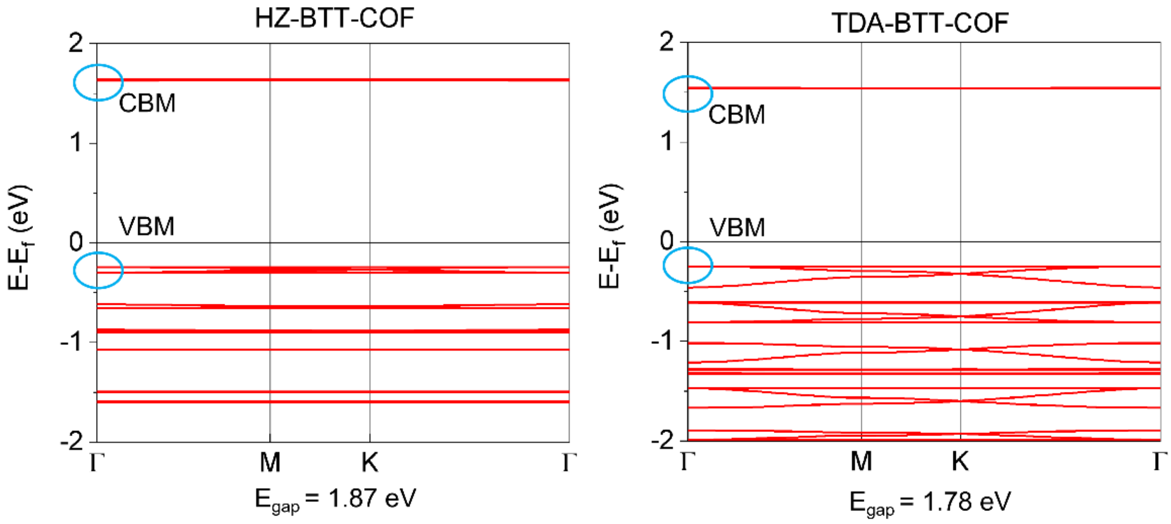

Fig. S35. The band structure of HZ-BTT-COF and TDA-BTT-COF by Perdew-Burke-Ernzerhof (PBE) functional.

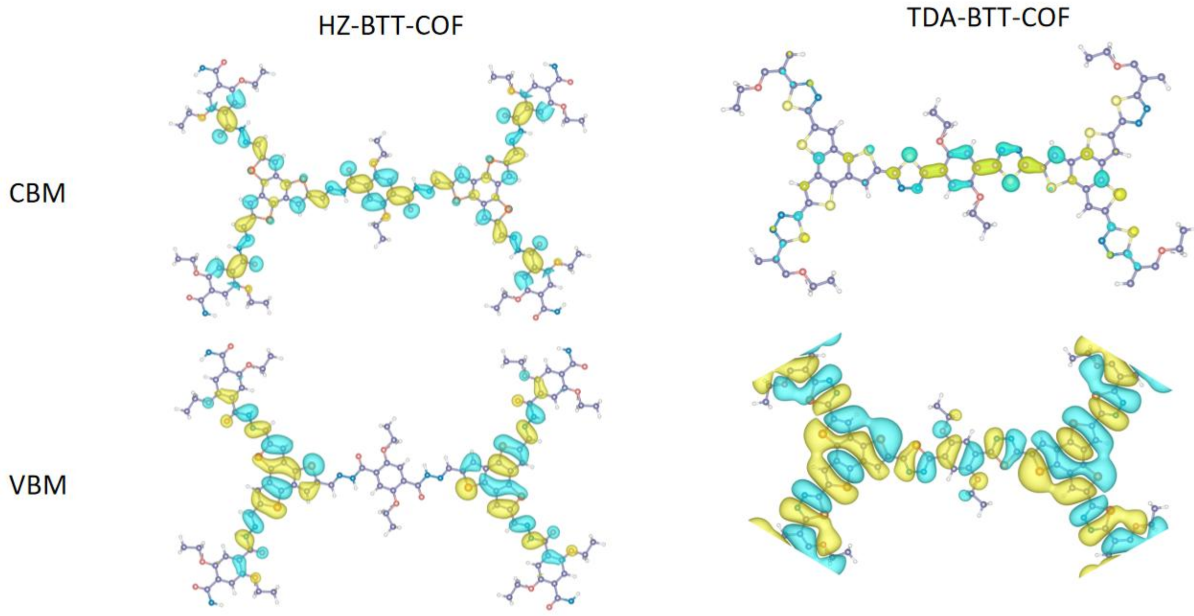

Fig. S36. The calculated conduction band minimum (CBM) and valence band maximum (VBM) diagrams of HZ-BTT-COF and TDA-BTT-COF.

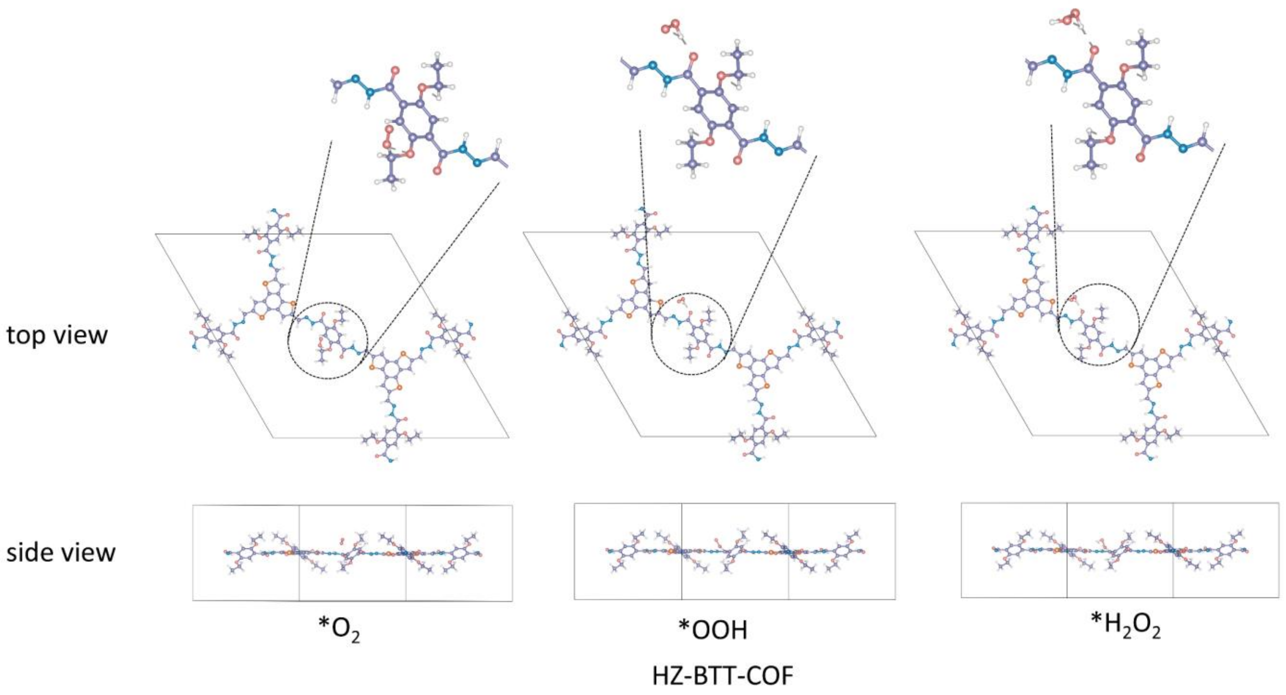

Fig. S37. The structures of $*O_2$, *OOH, and $*H_2O_2$ on HZ-BTT-COF during the oxygen reduction reaction (ORR) process.

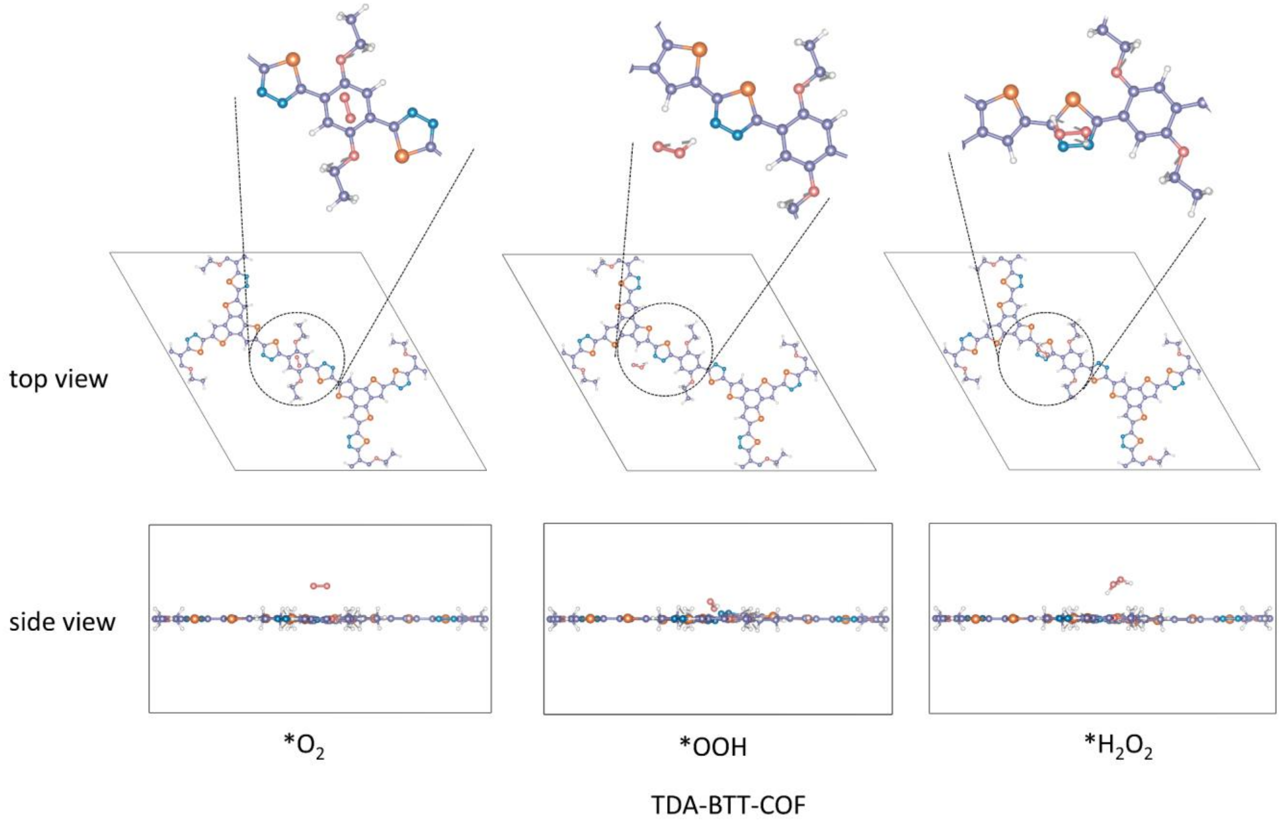

Fig. S38. The structures of $*O_2$, *OOH, and $*H_2O_2$ on TDA-BTT-COF during the ORR process.

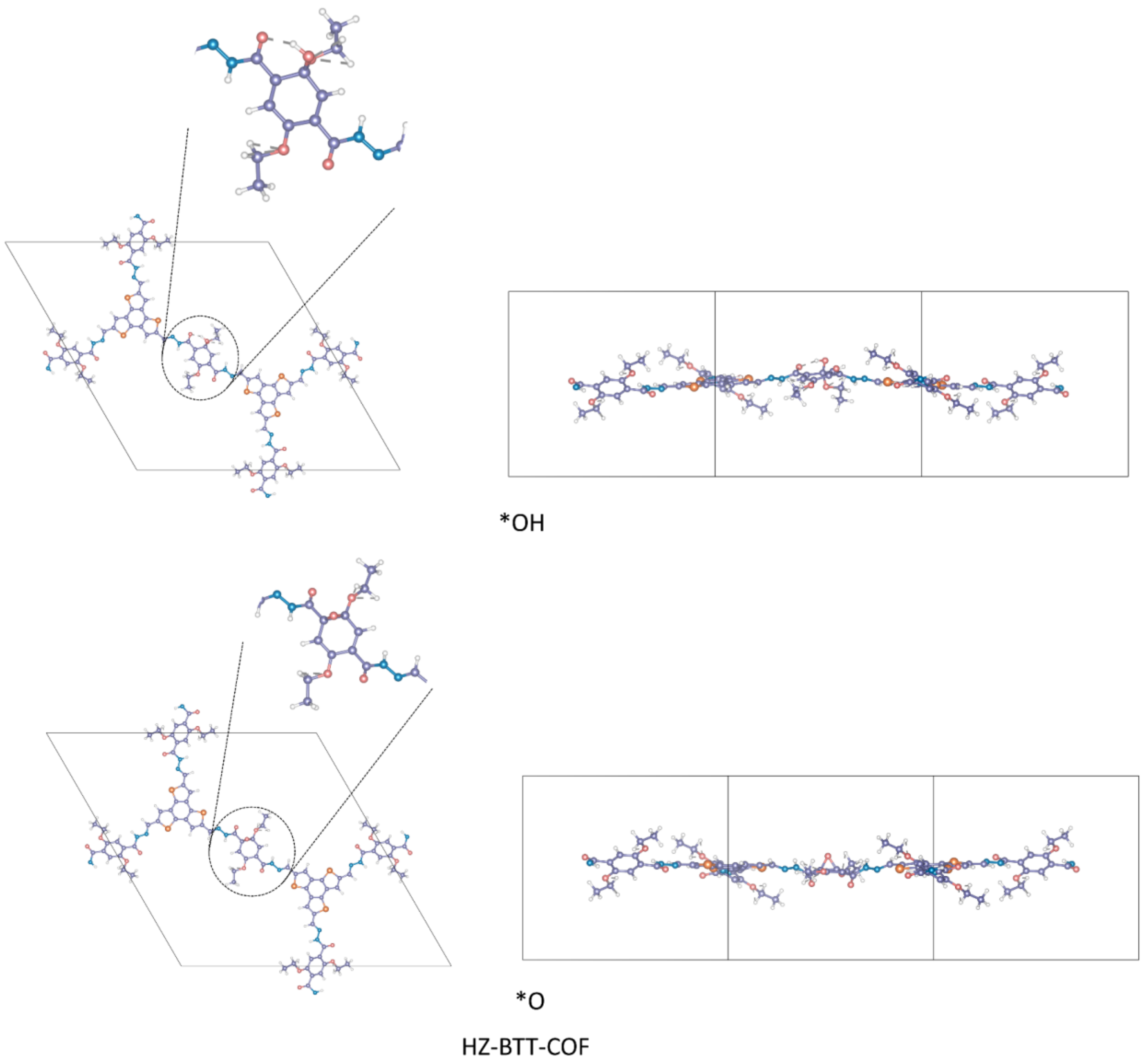

Fig. S39. The structure of *OH and *O on HZ-BTT-COF during the water oxidation reaction (WOR) process.

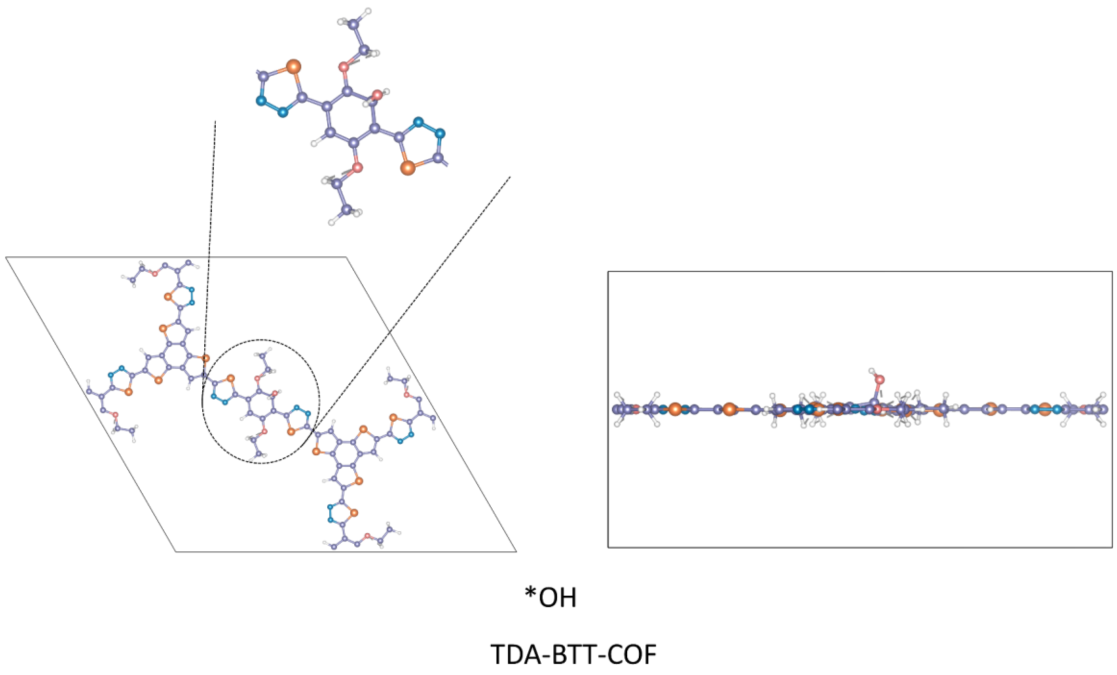

Fig. S40. The structure of *OH on TDA-BTT-COF during the WOR process.

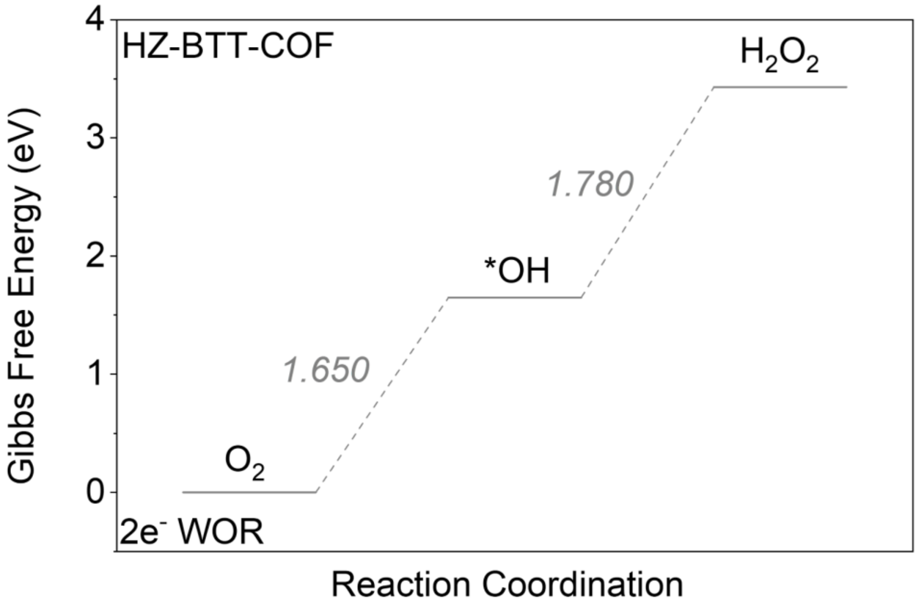

Fig. S41. Gibbs free energy diagrams of HZ-BTT-COF for direct H_2O_2 photogeneration by two-electron WOR pathway.

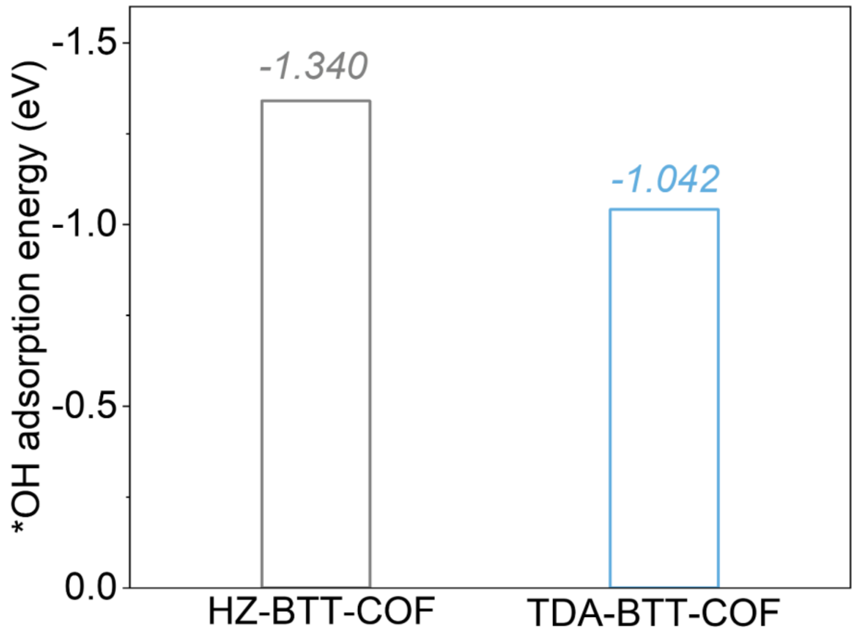

Fig. S42. *OH adsorption energy of HZ-BTT-COF and TDA-BTT-COF.

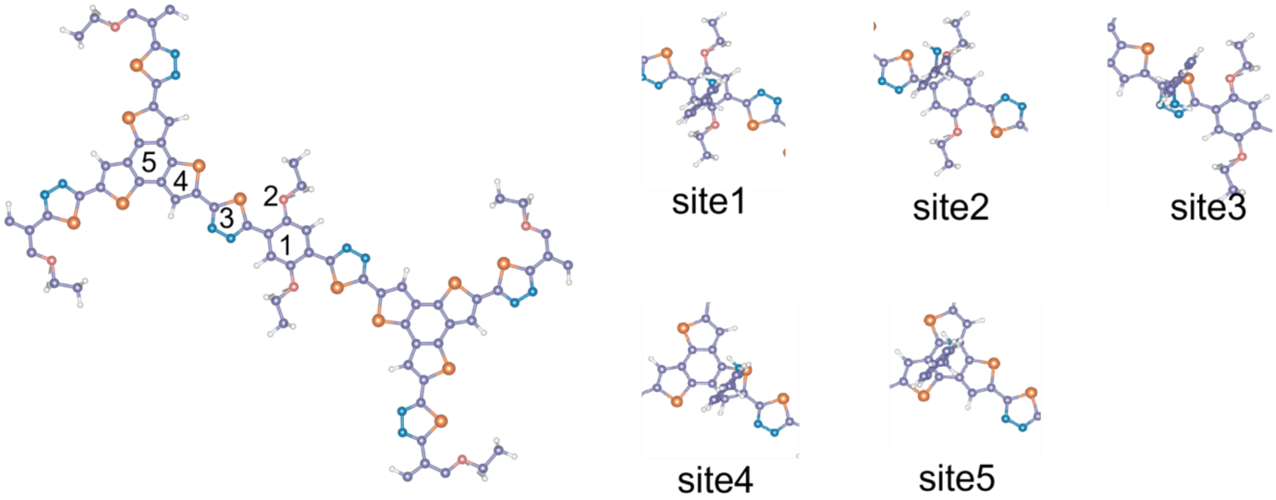

Fig. S43. Adsorption structure of benzylamine on TDA-BTT-COF at five different sites.

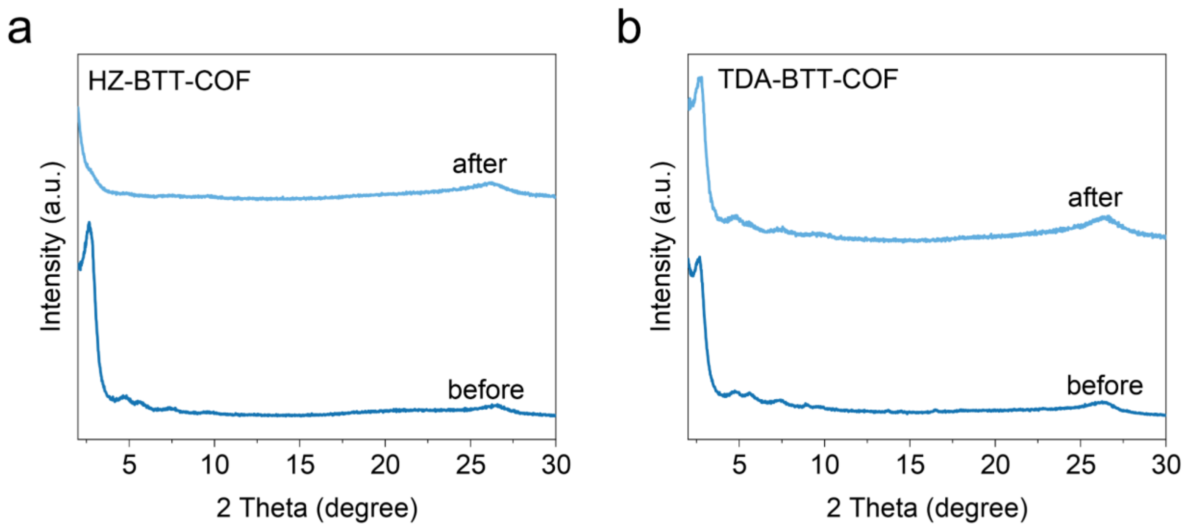

Fig. S44. PXRD pattern for a) HZ-BTT-COF and b) TDA-BTT-COF before and after four runs of benzylamine coupling (COFs were regenerated by washing with acetone and MeOH), a.u. indicates the arbitrary units.

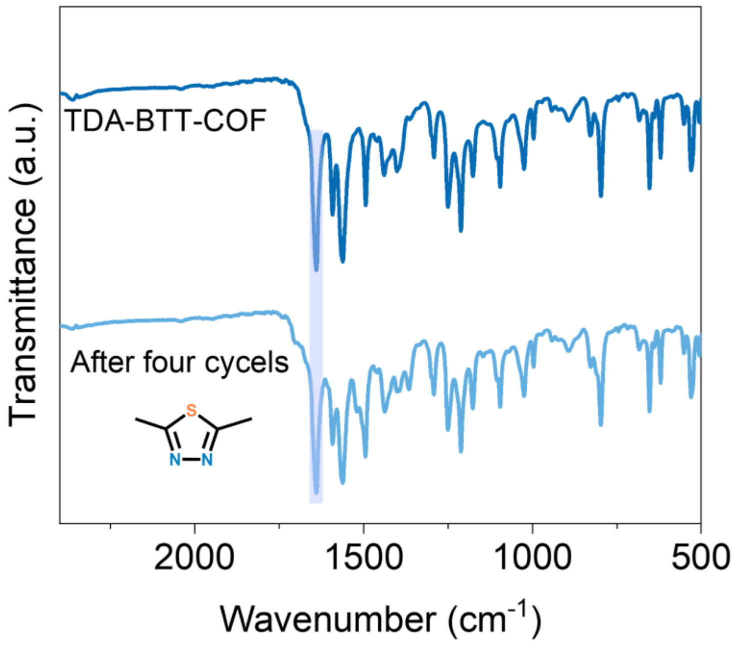

Fig. S45. FTIR spectra for TDA-BTT-COF before and after four runs of benzylamine coupling (COFs were regenerated by washing with acetone and MeOH), a.u. indicates the arbitrary units.

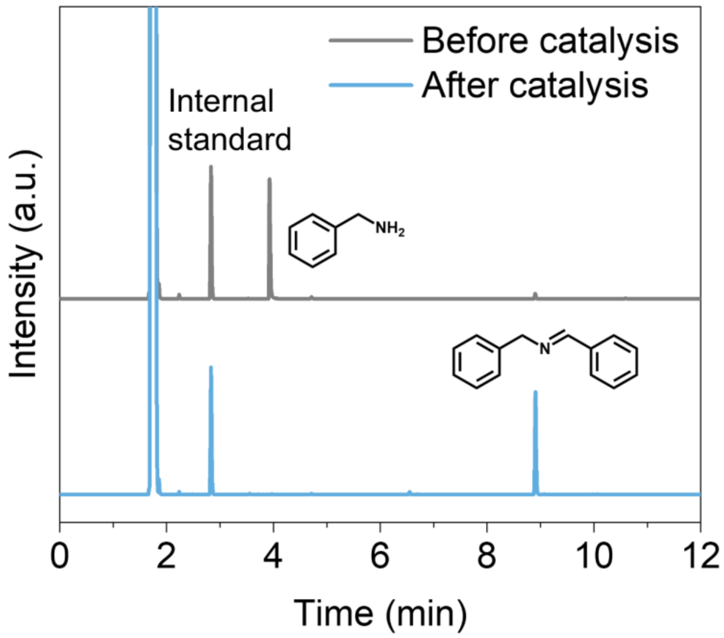

Fig. S46. GC of the reaction product using benzylamine as substrate, a.u. indicates the arbitrary units.

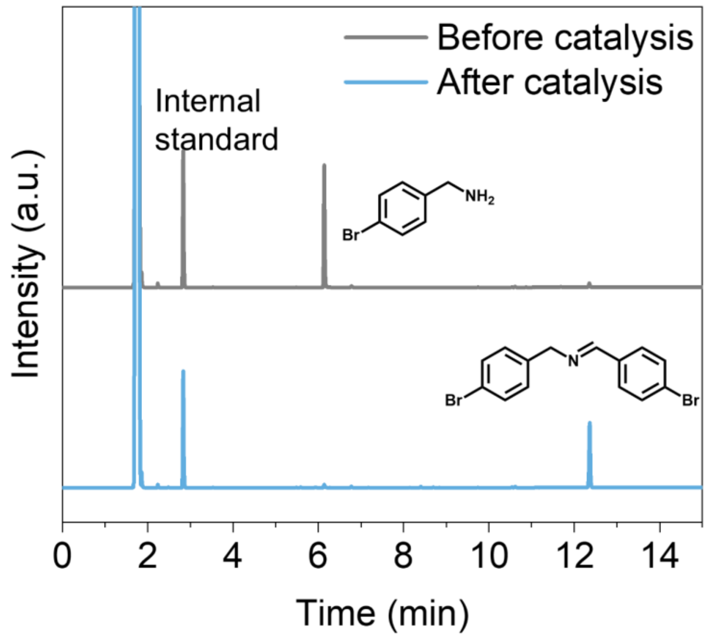

Fig. S47. GC of the reaction product using 4-bromobenzylamine as substrate, a.u. indicates the arbitrary units.

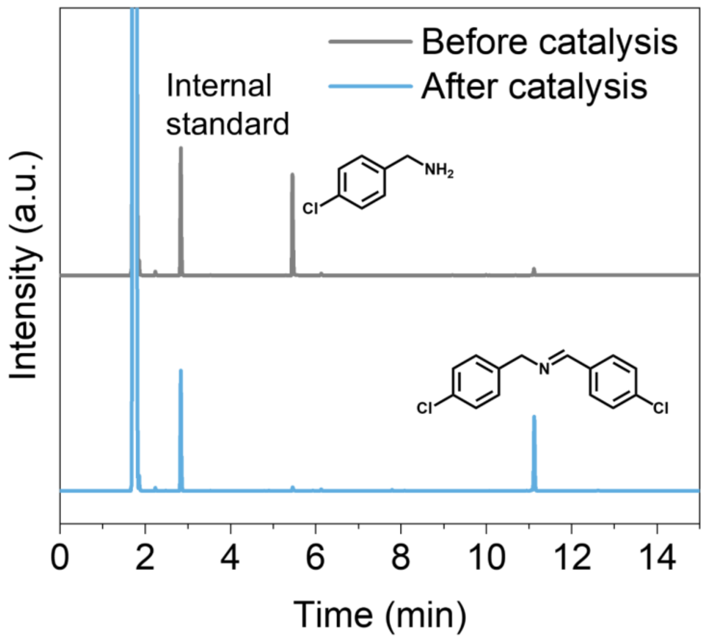

Fig. S48. GC of the reaction product using 4-chlorbenzylamine as substrate, a.u. indicates the arbitrary units.

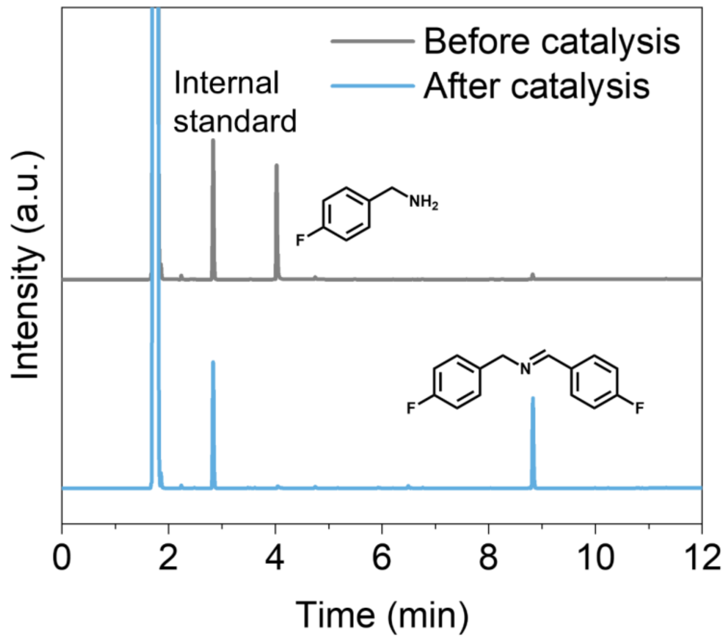

Fig. S49. GC of the reaction product using 4-fluorobenzylamine as substrate, a.u. indicates the arbitrary units.

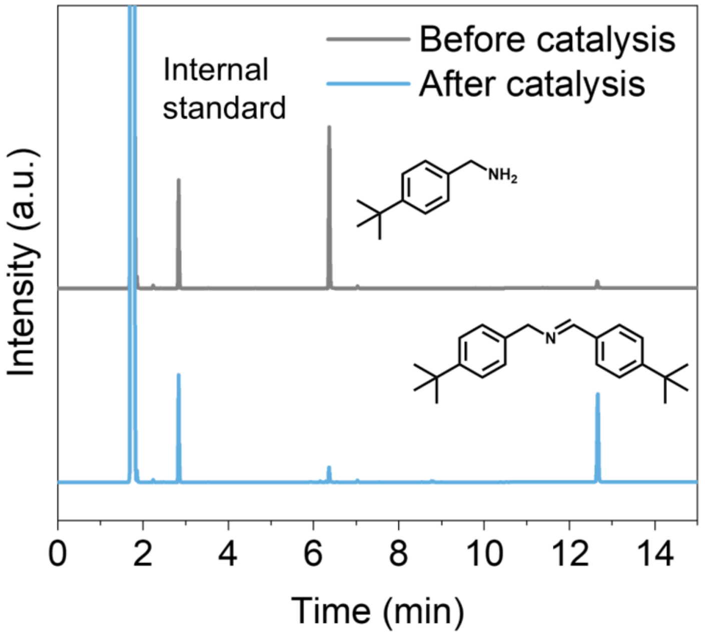

Fig. S50. GC of the reaction product using 4-tert-butylbenzylamine as substrate, a.u. indicates the arbitrary units.

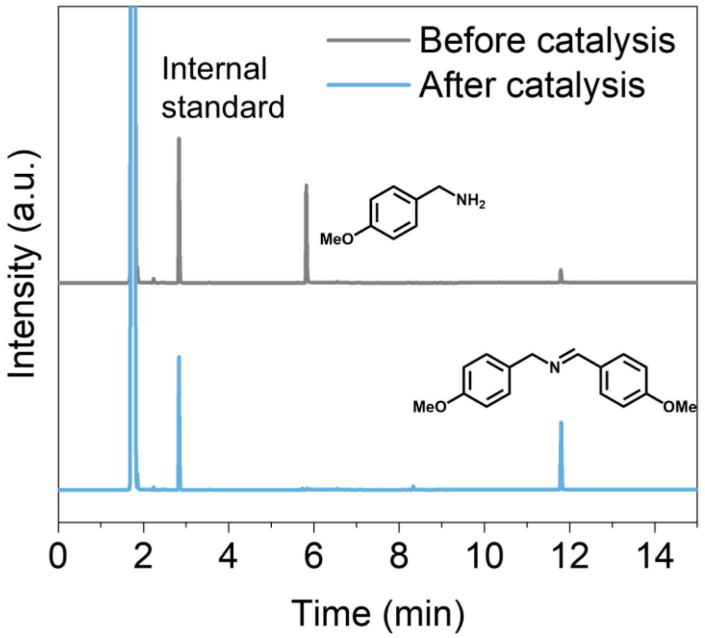

Fig. S51. GC of the reaction product using 4-methoxybenzylamine as substrate, a.u. indicates the arbitrary units.

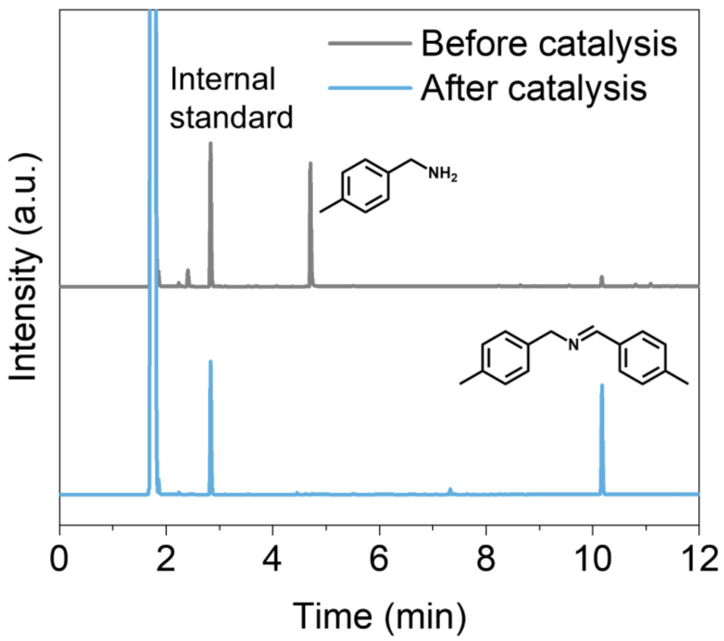

Fig. S52. GC of the reaction product using 4-methylbenzylamine as substrate, a.u. indicates the arbitrary units.

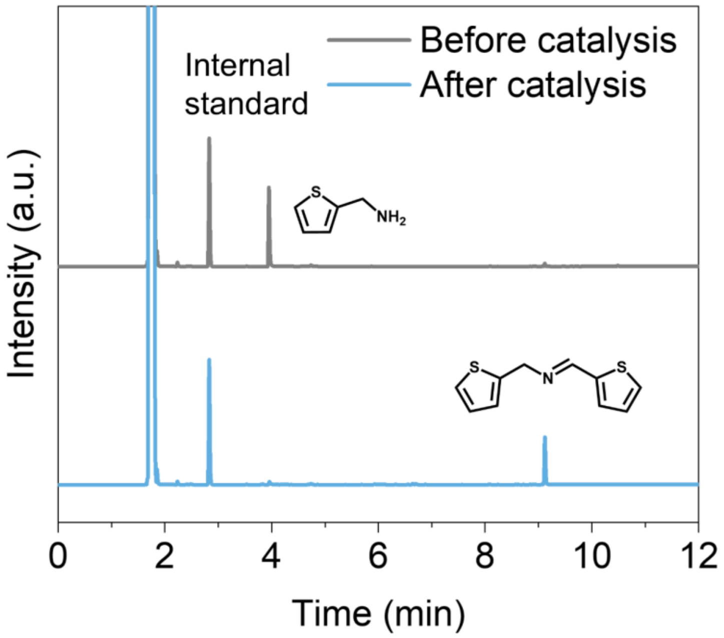

Fig. S53. GC of the reaction product using 2-thiophenemethylamine as substrate, a.u. indicates the arbitrary units.

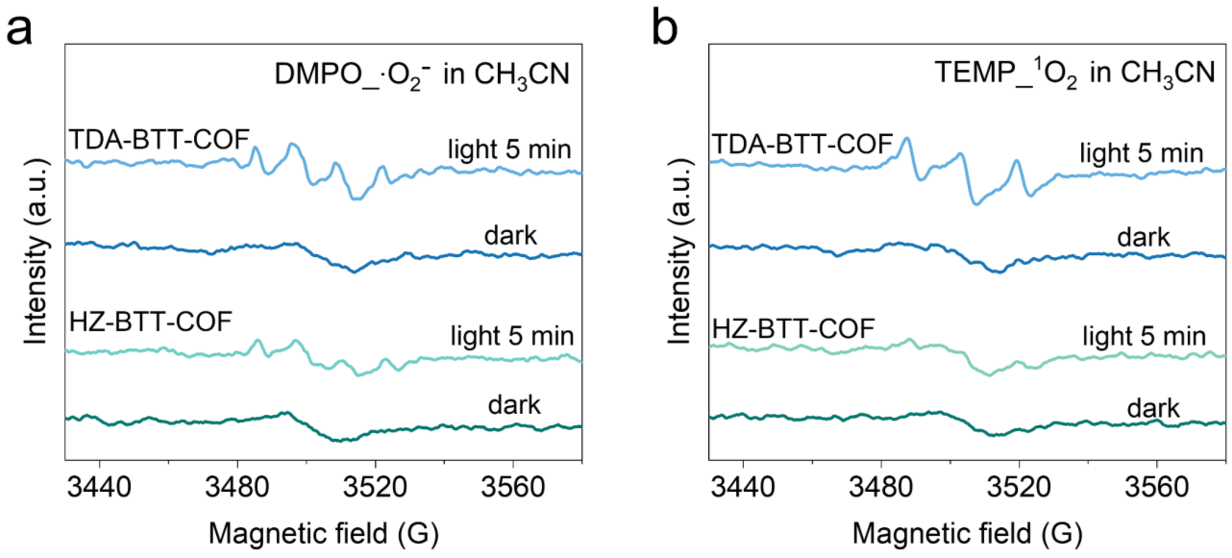

Fig. S54. EPR signals of the reaction solution under dark and light irradiation in the presence of a) DMPO and b) TEMP as the spin-trapping reagents. (300 W Xenon lamp, 5 min irradiation; O_2 saturated; CH_3CN; benzylamine), a.u. indicates the arbitrary units.

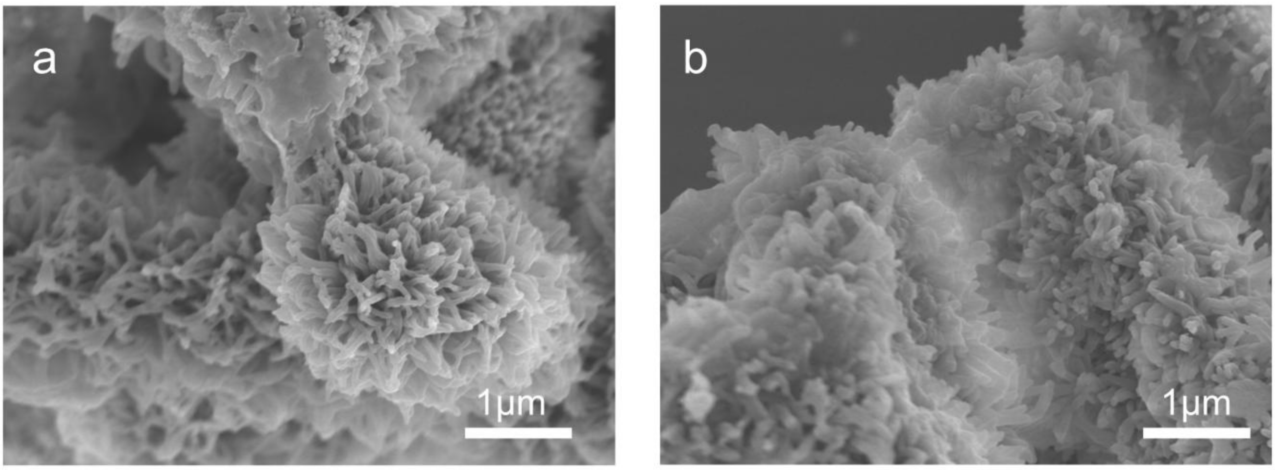

Fig. S55. SEM images of a) HZ-TTA-COF and b) TDA-TTA-COF.

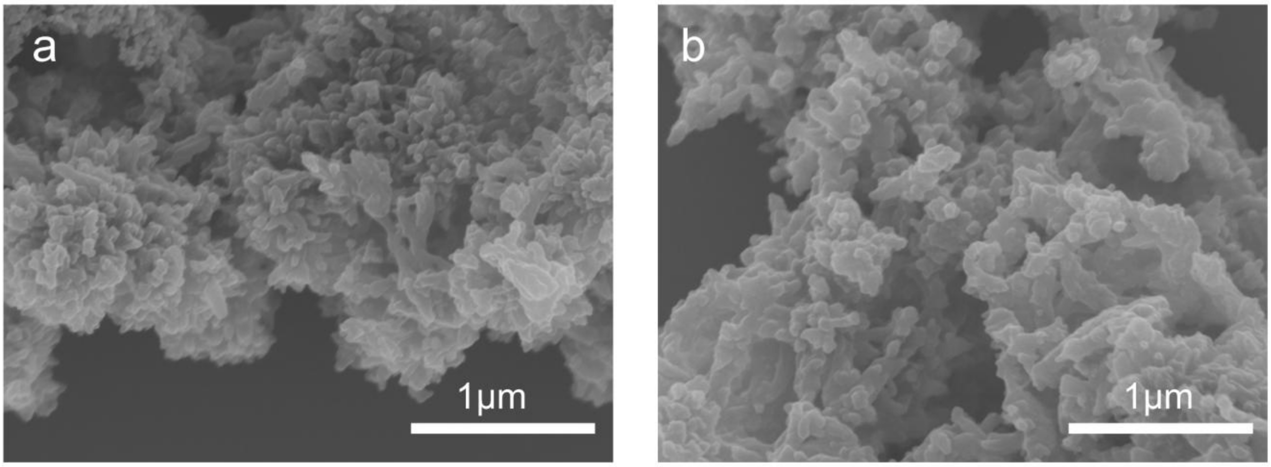

Fig. S56. SEM images of a) HZ-TFB-COF and b) TDA-TFB-COF.

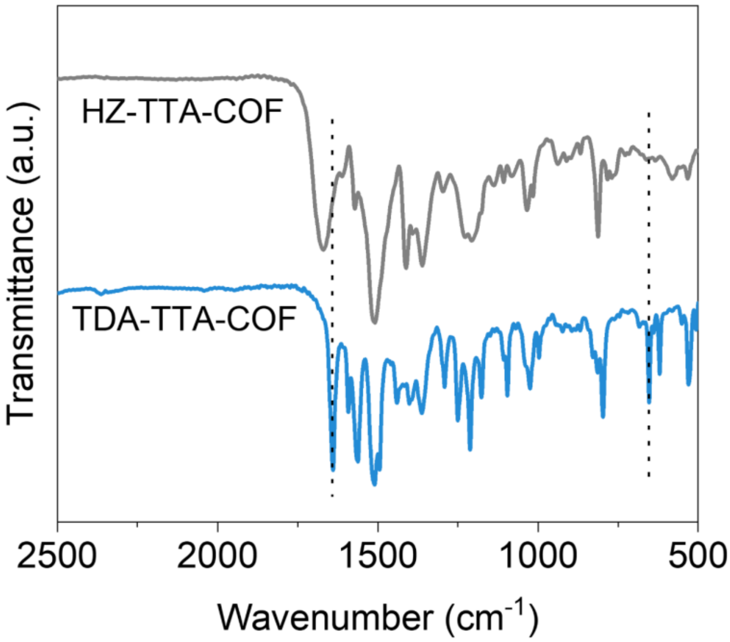

Fig. S57. FTIR spectra of HZ-TTA-COF and TDA-TTA-COF, a.u. indicates the arbitrary units.

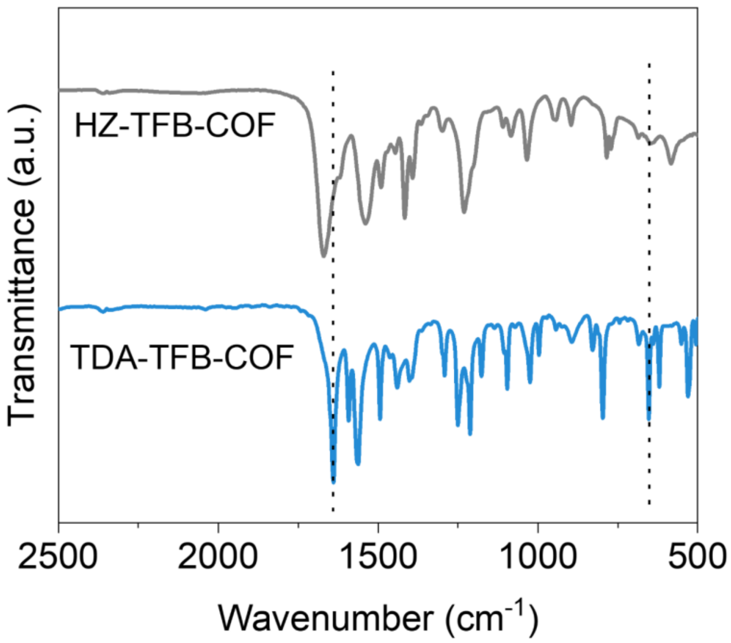

Fig. S58. FTIR spectra of HZ-TFB-COF and TDA-TFB-COF, a.u. indicates the arbitrary units.

Supplementary Tables

Table S1. Fractional atomic coordinates for TDA-BTT-COF.

TDA-BTT-COF				
Space Group: P6/M<br><br>a = 37.0460 Å, b = 37.0460 Å, c =3.4105 Å<br><br>α= 90 °, β= 90 °, γ= 120 °				
H1	H	0.61481	0.01626	0.23178
H2	H	0.67503	0.09493	0.76574
C25	C	0.29324	0.66576	0.5

C26	C	0.29381	0.62656	0.5
S27	S	0.33764	0.75708	0.5
C28	C	0.39392	0.77938	0.5
C29	C	0.40777	0.7487	0.5
C30	C	0.40246	0.57534	0.5
C31	C	0.4727	0.51927	0.5
C32	C	0.45455	0.47335	0.5
C33	C	0.481	0.45354	0.5
N34	N	0.37584	0.53153	0.5
N35	N	0.39787	0.51021	0.5
C36	C	0.44184	0.53712	0.5
S37	S	0.45722	0.59193	0.5
O38	O	0.59255	0.05536	0.5
C39	C	0.62072	0.03689	0.5
C40	C	0.66835	0.0738	0.5
H41	H	0.44182	0.75672	0.5
H42	H	0.41922	0.45296	0.5
H43	H	0.6907	0.05997	0.5

Table S2. Fractional atomic coordinates for HZ-BTT-COF.

HZ-BTT-COF				
Space Group: P6/M<br><br>a = 37.8298 Å, b = 37.8298 Å, c = 3.3955 Å<br><br>α= 90 °, β= 90 °, γ= 120 °				
H1	H	0.39204	0.41122	0.32061
H2	H	0.38649	0.34268	0.84603
H3	H	0.60796	0.58878	0.84848
H4	H	0.61351	0.65732	0.32305
C5	C	0.29869	0.67053	0.58454
C6	C	0.29454	0.63204	0.58454
S7	S	0.34775	0.75477	0.58454
C8	C	0.39881	0.76983	0.58454
C9	C	0.40718	0.73824	0.58454
C10	C	0.38164	0.5689	0.58454
N11	N	0.41946	0.57803	0.58454
N12	N	0.43079	0.54794	0.58454
C13	C	0.47133	0.55924	0.58454
C14	C	0.48613	0.52887	0.58454
C15	C	0.45855	0.48661	0.58454
C16	C	0.4716	0.45764	0.58454
O17	O	0.49624	0.59558	0.58454
O18	O	0.44376	0.41522	0.58454

C19	C	0.40092	0.40047	0.58454
C20	C	0.37812	0.35419	0.58454
H21	H	0.43752	0.74221	0.58454
H22	H	0.35753	0.53731	0.58454
H23	H	0.40772	0.51761	0.58454
H24	H	0.42652	0.47566	0.58454
H25	H	0.34476	0.34231	0.58454

Table S3. Performance and AQY comparison of TDA-BTT-COF with other photocatalysts reported in the literature for H_2O_2 production.

Photocatalysts	solution	Irradiation conditions /nm	H_2O_2 yields/ mmol g^{-1} h^{-1}	AQY (%)	Ref.
TDA-BTT-COF	Pure water	λ>420 nm	5.270	9.5% at 450 nm	This work
COF-2CN	Pure water	λ>420 nm	1.601	6.8% at 459 nm	[16]
TTF-BT-COF	Pure water	visible light	2.760	11.19% at 420 nm	[17]
COF-O	Pure water	30 W LED lamp (420 nm)	1.500	10.3% at 420 nm	[5]
H-COF	pH=2 water	λ>420 nm	2.114	2.75% at 600nm	[18]
FS-OHOMe-COF	pure water	λ>420 nm	1.480	9.6 % at 420 nm	[19]
FS-COF	pure water	λ>420 nm	3.904	6.21 % at 420 nm	[20]
TaptBtt	Pure water	AM 1.5G	1.407	4.6 % at 450 nm,	[4]
COF-N32	Pure water	λ>420 nm	0.605	6.2% at 459 nm	[21]

MeO-COF	Pure water	λ>420 nm	847.9	/	[22]
TDB-COF	Pure water	λ>420 nm	723.5	1.4% at 365 nm	[23]
T1H1.8-COF	Pure water	λ>420 nm	2567	9.5% at 420 nm	[24]
TMT-TT-COF	Pure water	λ>420 nm	1952	/	[25]
COF–BPDA–DTP	Pure water	λ>420 nm	1164	/	[26]
TAPT-DHA	Pure water	λ>420 nm	1629	7.79% at 450 nm	[27]
Tz–THBZ	Pure water	λ>420 nm	2580	5.5% at 420 nm	[28]
TTA-TF-COF	Pure water	λ>420 nm	3343	4.1% at 420 nm	[29]
TZ-40	Pure water	λ>420 nm	3816	/	[30]
TTB-TTA-Ph-3F	Pure water	λ>420 nm	3496.9	13.0% at 420 nm	[31]
BT-COF	Pure water	λ>420 nm	4524	5.2% at 365 nm	[32]
COF-OME	Pure water	λ>420 nm	4138	6.5 % at 400 nm	[33]
2-SO_3H-COF	Pure water	λ>420 nm	2915	5.3% at 420 nm	[34]
PDCOF-2 ph	Pure water	λ>420 nm	4668	3.8 % at 400 nm	[35]
Mph-2CN-COF	Pure water	λ>420 nm	2887	5 % at 400 nm	[36]
Py-OH-Sa COF	Pure water	300 W, full spectrum	4780	6.52 % at 380 nm	[37]
Tp-TTz CO	Pure water	300 W, full spectrum	2300	16.5% at 420 nm	[38]

NT: Not Tested

Table S4. Photocatalyzed aerobic oxidation of primary amines to imines by HZ-BTT-COF and TDA-BTT-COF.

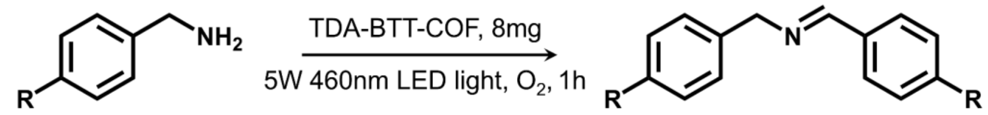

Entry	Substrate	Product	TDA-BTT-COF Conv. (%)	TDA-BTT-COF Sel. (%)	HZ-BTT-COF Conv. (%)	HZ-BTT-COF Sel. (%)
1	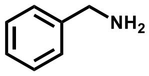	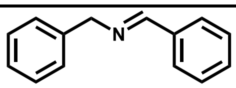	100%	100%	72%	100%
2	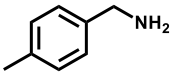	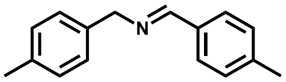	98%	99%	65%	95%
3	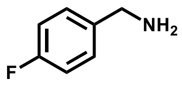	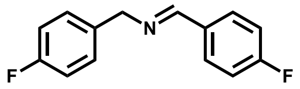	97%	99%	54%	99%
4	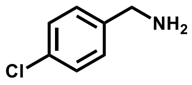	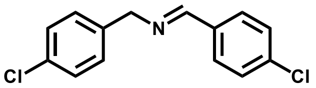	95%	98%	62%	94%
5	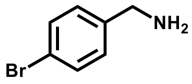	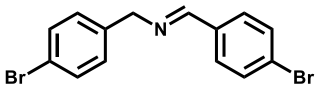	96%	99%	66%	96%
6	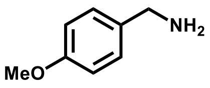	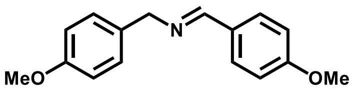	98%	94%	58%	99%
7	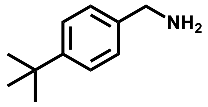	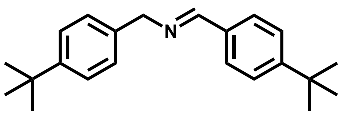	93%	99%	62%	99%
8	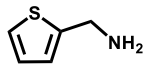	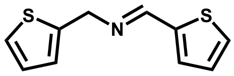	95%	90%	61%	90%

Table S5. Control experiments for the photocatalytic oxidative coupling of benzylamine by TDA-BTT-COF.

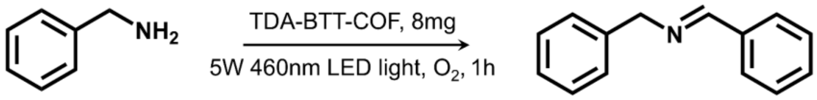

Entry	Reaction condition Variations	Light	Time(h)	Conversion (%)
1	-	Off	1	-
2	no photocatalyst	On	1	-
3	N_2	On	1	25%

References:

(1) Ko, I.; Park, S.; Lee, G.; Kim, H., An efficient one-pot synthesis of 2,5-disubstituted-1,3,4-thiadiazoles from aldehydes and hydrazides using Lawesson's reagent. *ARKIVOC* **2019,** *2019* (3), 67-78.

(2) Yang, S.; Chen, Z.; Zou, L.; Cao, R., Construction of Thiadiazole-Linked Covalent Organic Frameworks via Facile Linkage Conversion with Superior Photocatalytic Properties. *Adv. Sci.* **2023,** *10* (31), 2304697.

(3) Zhang, L.; Wang, C.; Jiang, Q.; Lyu, P.; Xu, Y., Structurally Locked High-Crystalline Covalent Triazine Frameworks Enable Remarkable Overall Photosynthesis of Hydrogen Peroxide. *J. Am. Chem. Soc.* **2024,** *146* (43), 29943-29954.

(4) Qin, C.; Wu, X.; Tang, L.; Chen, X.; Li, M.; Mou, Y.; Su, B.; Wang, S.; Feng, C.; Liu, J.; Yuan, X.; Zhao, Y.; Wang, H., Dual donor-acceptor covalent organic frameworks for hydrogen peroxide photosynthesis. *Nat. Commun.* **2023,** *14* (1), 5238.

(5) Xie, Z.; Chen, X.; Wang, W.; Ke, X.; Zhang, X.; Wang, S.; Wu, X.; Yu, J. C.; Wang, X., Variation of Chemical Microenvironment of Pores in Hydrazone-Linked Covalent Organic Frameworks for Photosynthesis of H_2O_2. *Angew. Chem. Int. Ed.* **2024,** *63* (39), e202410179.

(6) Kresse, G.; Hafner, J., Ab initio molecular-dynamics simulation of the liquid-metal--amorphous-semiconductor transition in germanium. *Phys. Rev. B* **1994,** *49* (20), 14251-14269.

(7) Kresse, G.; Furthmüller, J., Efficiency of ab-initio total energy calculations for metals and semiconductors using a plane-wave basis set. *Comput. Mater. Sci.* **1996,** *6* (1), 15-50.

(8) Kresse, G.; Furthmüller, J., Efficient iterative schemes for ab initio total-energy calculations using a plane-wave basis set. *Phys. Rev. B* **1996,** *54* (16), 11169-11186.

(9) Blöchl, P. E., Projector augmented-wave method. *Phys. Rev. B* **1994,** *50* (24), 17953-17979.

(10) Perdew, J. P.; Chevary, J. A.; Vosko, S. H.; Jackson, K. A.; Pederson, M. R.; Singh, D. J.; Fiolhais, C., Atoms, molecules, solids, and surfaces: Applications of the generalized gradient approximation for exchange and correlation. *Phys. Rev. B* **1992,** *46* (11), 6671-6687.

(11) Klimeš, J.; Bowler, D. R.; Michaelides, A., Chemical accuracy for the van der Waals density functional. *J. Phys.: Condens. Matter* **2010,** *22* (2), 022201.

(12) Klimeš, J.; Bowler, D. R.; Michaelides, A., Van der Waals density functionals applied to solids. *Phys. Rev. B* **2011,** *83* (19), 195131.

(13) Bader, R. F. W., A quantum theory of molecular structure and its applications. *Chem. Rev.* **1991,** *91* (5), 893-928.

(14) Tang, W.; Sanville, E.; Henkelman, G., A grid-based Bader analysis algorithm without lattice bias. *J. Phys.: Condens. Matter* **2009,** *21* (8), 084204.

(15) Wang, V.; Xu, N.; Liu, J.-C.; Tang, G.; Geng, W.-T., VASPKIT: A user-friendly interface facilitating high-throughput computing and analysis using VASP code. *Comput. Phys. Commun.* **2021,** *267*, 108033.

(16) Hou, Y.; Zhou, P.; Liu, F.; Lu, Y.; Tan, H.; Li, Z.; Tong, M.; Ni, J., Efficient Photosynthesis of Hydrogen Peroxide by Cyano-Containing Covalent Organic Frameworks from Water, Air and Sunlight. *Angew. Chem. Int. Ed.* **2024,** *63* (6), e202318562.

(17) Chang, J.-N.; Li, Q.; Shi, J.-W.; Zhang, M.; Zhang, L.; Li, S.; Chen, Y.; Li, S.-L.; Lan, Y.-Q., Oxidation-Reduction Molecular Junction Covalent Organic Frameworks for Full Reaction Photosynthesis of H_2O_2. *Angew. Chem. Int. Ed.* **2023,** *62* (9), e202218868.

(18) Zhu, Q.; Shi, L.; Li, Z.; Li, G.; Xu, X., Protonation of an Imine-linked Covalent Organic Framework for Efficient H_2O_2 Photosynthesis under Visible Light up to 700 nm. *Angew. Chem. Int. Ed.* **2024,** *63* (32), e202408041.

(19) Shu, C.; Yang, X.; Liu, L.; Hu, X.; Sun, R.; Yang, X.; Cooper, A. I.; Tan, B.; Wang, X., Mixed-Linker Strategy for the Construction of Sulfone-Containing D–A–A Covalent Organic Frameworks for Efficient Photocatalytic Hydrogen Peroxide Production. *Angew. Chem. Int. Ed.* **2024,** *63* (22), e202403926.

(20) Luo, Y.; Zhang, B.; Liu, C.; Xia, D.; Ou, X.; Cai, Y.; Zhou, Y.; Jiang, J.; Han, B., Sulfone-Modified Covalent Organic Frameworks Enabling Efficient Photocatalytic Hydrogen Peroxide Generation via One-Step Two-Electron O_2 Reduction. *Angew. Chem. Int. Ed.* **2023,** *62* (26), e202305355.

(21) Liu, F.; Zhou, P.; Hou, Y.; Tan, H.; Liang, Y.; Liang, J.; Zhang, Q.; Guo, S.; Tong, M.; Ni, J., Covalent organic frameworks for direct photosynthesis of hydrogen peroxide from water, air and sunlight. *Nat. Commun.* **2023,** *14* (1), 4344.

(22) Li, C.; Xie, H.; Zhou, S.; Hu, H.; Chen, G.; Wei, Z.; Jiang, J.; Qin, J.; Zhang, Z.; Kong, Y., The structure-activity relationship for the electron-donating functional groups in hydrazone-linked COFs and their photocatalytic H_2O_2 production. *Mater. Res. Bull.* **2024,** *173*, 112697.

(23) Zhou, Z.; Sun, M.; Zhu, Y.; Li, P.; Zhang, Y.; Wang, M.; Shen, Y., A thioether-decorated triazine-based covalent organic framework towards overall H_2O_2 photosynthesis without sacrificial agents. *Appl. Catal., B* **2023,** *334*, 122862.

(24) Zhou, S.; Shi, Y.; Chen, G.; Kong, W.; Hu, H.; Xie, H.; Li, C.; Qin, J.; Zhang, Z.; Peng, L.; Ke, X.; Kong, Y., Fragmentation engineering on the edge of hydroxy-functional COFs for the enhanced photocatalytic production of H_2O_2 and direct photo-oxidation of benzene to phenol in aqueous systems. *Chem. Eng. J.* **2023,** *477*, 146946.

(25) Deng, M.; Wang, L.; Wen, Z.; Chakraborty, J.; Sun, J.; Wang, G.; Van Der Voort, P., Donor–acceptor sp^2 covalent organic frameworks for photocatalytic H_2O_2 production and tandem bisphenol-A degradation. *Green Chem.* **2024,** *26* (6), 3239-3248.

(26) Yang, T.; Wang, Y.; Chen, Y.; Peng, X.; Zhang, H.; Kong, A., Linker length-dependent hydrogen peroxide

photosynthesis performance over crystalline covalent organic frameworks. *CrystEngComm* **2023,** *25* (32), 4511-4520.
(27) Bai, X.; Guo, L.; Jia, T.; Hu, Z., Superhydrophilic covalent organic frameworks accelerate photocatalytic production of hydrogen peroxide through proton channels. *J. Mater. Chem. A* **2024,** *12* (22), 13116-13126.
(28) Shu, C.; Xie, P.; Yang, X.; Yang, X.; Gao, H.; Tan, B.; Wang, X., Integrating β-ketoenamine linkages into covalent organic frameworks toward efficient overall photocatalytic hydrogen peroxide production. *J. Mater. Chem. A* **2024,** *12* (38), 25927-25933.
(29) Yang, X.; Pan, Z.-X.; Yue, J.-Y.; Li, X.; Liu, G.; Xu, Q.; Zeng, G., Nitrogen-Site Engineering in Covalent Organic Frameworks for H_2O_2 Photogeneration via Dual Channels of Indirect Two-Electron O_2 Reduction. *Small* **2024,** *20* (47), 2405907.
(30) Ma, X.; Li, S.; Gao, Y.; Li, N.; Han, Y.; Pan, H.; Bian, Y.; Jiang, J., S-Scheme Heterojunction Fabricated from Covalent Organic Framework and Quantum Dot for Enhanced Photosynthesis of Hydrogen Peroxide from Water and Air. *Adv. Funct. Mater.* **2024,** *34* (51), 2409913.
(31) Rong, Q.; Chen, X.; Cheng, Q.; Huang, Z.; He, S., Modulating Quinoline-Linked Covalent Organic Frameworks via Fluorination for Boosting the Photocatalytic Air Reductive H_2O_2 Production. *ACS Sustainable Chem. Eng.* **2024,** *12* (35), 13306-13315.
(32) Xiong, K.; Jia, X.; Kong, Y.; Yang, J.; Guo, J.; Li, S.; Adeli, M.; Wang, Y.; Luo, X.; Han, X.; Cheng, C., Customized Covalent Organic Frameworks with in-Plane and Intramolecular Asymmetric Polarization for Superior Hydrogen Peroxide Photosynthesis and Aerobic Oxidation of Alcohol. *Adv. Funct. Mater.* **2025,** *35* (46), 2510257.
(33) Guo, M.; He, C.; Wu, Z.; Tian, Y.; Yang, J.; Wang, Y.; Wu, H.; Yang, J.; Xu, M.; Xue, W.; Cheng, C.; Li, S.; Zhao, C., Micro-Environment Programmable Quinoline COFs for High-Performance Photocatalytic H_2O_2 Generation and Benzylamine Coupling. *Adv. Sci.* **2025,** *12* (33), e05794.
(34) Yang, K.; Chen, L.; Xiong, K.; Yang, J.; Adeli, M.; Li, S.; Wang, M.; Cheng, C.; Zhao, C., Bisite Sulfonate-Functionalized Covalent Organic Frameworks with Efficient and Dynamic Proton Transportation for Accelerating H_2O_2 Photosynthesis. *Adv. Funct. Mater.* **2025,** *n/a* (n/a), e20900.
(35) Du, Y.; Wu, Z.; Wang, Y.; Yang, J.; You, H.; Kong, Y.; Wu, H.; Wang, Y.; Cheng, C.; Li, S.; Zhao, C., Unlocking Electronic Confinement in Phenanthroline-π-Bridged Covalent Organic Frameworks Enables Efficient Photocatalytic H_2O_2 Generation and Oxidation of Sulfides. *Small* **2026,** *22* (6), e12201.
(36) Zhou, L.; Jia, X.; Wang, Y.; Xiong, K.; Yang, J.; Kong, Y.; Guo, M.; Xu, X.; Li, S.; Ren, X.; Cheng, C., Tailoring cyano-functionalized covalent organic frameworks with highly ordered charge network structure for efficient hydrogen peroxide photosynthesis. *Mater. Sci. Eng.: R: Rep.* **2026,** *167*, 101117.
(37) Huang, P.; Peng, Y.-Y.; Wang, X.-H.; Li, R.-H.; Qin, M.-H.; Zhang, M.; Wang, S.-M.; Lu, M.; Li, S.-L.; Lan, Y.-Q., Charge-Distribution and Microenvironment Dual Regulation of Covalent Organic Frameworks for Enhancing Photocatalytic H_2O_2 and H_2 Production. *Adv. Mater.* **2026,** *38* (3), e07849.
(38) Xue, Z.; Zhang, B.; Guo, Q.; Wang, Y.; Li, Q.; Yang, K.; Qiao, S., Sacrificial-Agent-Triggered Mass Transfer Gating in Covalent Organic Framework for Hydrogen Peroxide Photocatalysis. *Adv. Mater.* **2025,** *37* (42), e10201.
